# Molecular Drivers of Electron-Donating Capacity in Dissolved Black Carbon from Nitrogen-Rich Pyrogenic Carbon

**DOI:** 10.1021/acs.est.5c09050

**Published:** 2025-12-10

**Authors:** Xiaoxiao Zhang, Weijian Xu, Wenjing Tian, Eakalak Khan, Daniel C.W. Tsang

**Affiliations:** a Department of Civil and Environmental Engineering, 58207 The Hong Kong University of Science and Technology, Clear Water Bay, Hong Kong,China; b Department of Civil and Environmental Engineering, 26680 The Hong Kong Polytechnic University, Hung Hom, Kowloon, Hong Kong, China; c Department of Civil and Environmental Engineering and Construction, University of Nevada, Las Vegas, Nevada 89154-4015, United States

**Keywords:** dissolved pyrogenic carbon, molecular signatures, surface functional groups, heterogeneous correlations, dynamic leaching conditions, black carbon structures

## Abstract

Dissolved black carbon (DBC) from nitrogen-rich feedstock-derived pyrogenic carbon may influence aquatic photochemistry and byproduct formation due to its electron-donating capacity (EDC). Yet, the molecular drivers of EDC remain unclear. Here, we developed an integrated analytical framework to characterize DBC leached from nitrogen-rich biochar pyrolyzed at 350, 450, and 550 °C (DBC350, DBC450, and DBC550) under simulated intermittent rainfall over 30 days. Through two-dimensional correlation spectroscopy (2D-COS) and Fourier transform ion cyclotron resonance mass spectrometry, we analyzed sequential responses and synergistic relationships of thousands of individual DBC molecules with various functional groups. The EDC increased with leaching time, particularly in DBC350, coinciding with a shift toward lower *m*/*z*, more unsaturated, and aromatic compounds. Spearman’s analysis showed that EDC-related molecules were predominantly nitrogen-bearing (61–76%), highly unsaturated, and low-oxygen. Our 2D-COS analysis on EDC-related molecules and functional groups identified (hetero)­aromatic structures as key EDC contributors. Tandem mass spectrometry and X-ray photoelectron spectroscopy further confirmed the prevalence of carboxylic, pyrrolic, and/or amide groups. Extended (hetero)­aromatic structures contributed to the higher EDC in DBC350 than in DBC450 and DBC550. Our study offers the first molecular and functional group-level insight into EDC-related DBC compositions, with implications for biochar-related and postwildfire water quality management.

## Graphical abstract

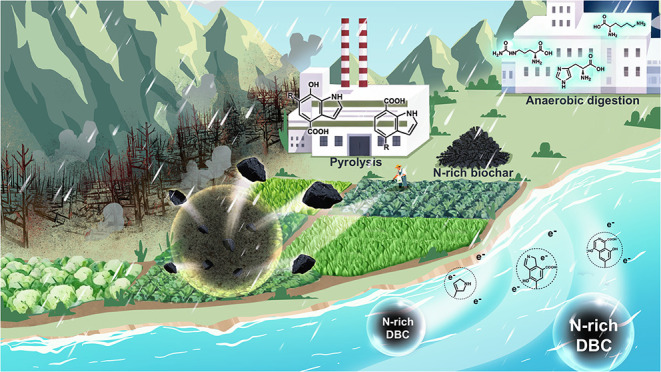

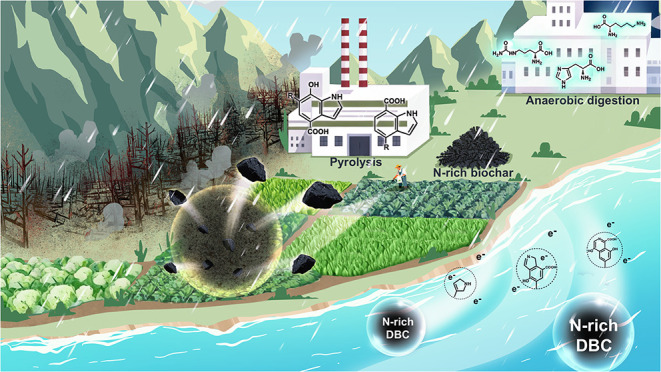

## 1 Introduction

The conversion of biomass waste into biochar enhances soil carbon sequestration and agricultural productivity.^1–3^ Biochar derived from nitrogen-rich feedstocks (e.g., food waste digestate) has emerged as a promising soil amendment.^4–6^ Yet, its application may release dissolved black carbon (DBC) and nitrogen-bearing fractions into aquatic systems.^7–9^ Beyond anthropogenic sources, natural events such as wildfires and volcanic eruptions also contribute substantial amounts of pyrogenic DBC to surface waters.^10–12^ Pyrogenic DBC is highly resistant to biotransformation, leading to its accumulation in aquatic systems.^13,14^ This persistence promoted the formation of photoreactive species under sunlight irradiation,^15,16^ and toxic disinfection byproducts during drinking water treatment.^14,17^ The enrichment of nitrogen in DBC is arousing concerns,^11,18^ given the greater reactivity of heteroaromatic nitrogen structures,^8^ and the higher toxicity of nitrogenous disinfection byproducts compared to their carbonaceous counterparts.^14,17,19^ These risks underscore the need to understand the molecular and structural compositions of DBC, especially its nitrogen-rich fractions, to evaluate the ecological and water quality impacts of biochar applications and natural wildfires.

Electron-donating capacity (EDC) serves as a proxy for predicting photoreactive intermediates^20^ and disinfection byproduct precursor content.^21,22^ DBC typically exhibits higher EDC than humic substances,^23^ corresponding to elevated photoactivity and byproduct formation.^14,24^ Pyrolysis temperature plays a key role in determining the EDC of DBC.^25^ For instance, grass char-derived DBC exhibits peak EDC at 300 °C, with higher or lower temperatures reducing the EDC.^25^ The chemical stability of pyrogenic carbon enables continuous and repeated leaching of DBC during intermittent rainfall events and/or irrigation activities, leading to prolonged environmental impacts.^5,26,27^ For example, leaching over time altered the phytotoxicity of DBC and byproduct formation.^28,29^ In addition to pyrogenic temperatures, repeated leaching cycles may significantly alter EDC, warranting further attention. Despite increasing attention on EDC of DBC derived from biochar,^25,30^ limited knowledge is available for DBC from nitrogen-rich feedstock derived biochar.^9,11^ Moreover, most of the existing studies assess EDC based on a single extraction,^20^ overlooking the significance of temporal changes during intermittent rainfall events.

Shifts in molecular compositions, such as size, largely drive the changes in EDC.^31,32^ Understanding the molecular features that influence EDC is essential for predicting photoreactive intermediates and unknown byproducts.^31,33,34^ Recent studies have highlighted the compositional differences among EDC-contributing molecules in dissolved organic matter (DOM) from natural waters and effluents.^35^ However, little is known about how repeated leaching cycles would influence the molecular characteristics of DBC that govern the EDC. Although broad compositional variations have been reported,^35,36^ information regarding specific molecular contributors to EDC remains limited. Linking molecular features to EDC or EDC-related properties may help identify these contributors.^37–39^ The neutral loss-based functionality analysis offers a promising strategy for inferring key structural features.^40^ To the best of our knowledge, no studies have linked EDC to individual DBC molecules derived from nitrogen-rich feedstock derived biochar, nor have they combined neutral loss-based methods to identify the dominant structural drivers of EDC.

Functional groups of DBC are key structural determinants of the EDC variability, significantly influencing its photoactivity and the toxicity of halogenated byproducts.^8,41^ While phenolic groups have traditionally been considered the primary contributors to EDC due to their strong linear correlation with EDC levels,^23,32^ this assumption warrants reconsideration, especially regarding DBC.^23^ For instance, the high intercept in the linear correlation between EDC and phenolic (Ar–OH) content of DBC from biomass-derived biochar (y = 0.112Ar–OH + 2.23, *r* = 0.98) suggests a substantial baseline EDC contribution from nonphenolic groups, which can be equivalent to the EDC of 20 mmol Ar–OH, comparable to the measured phenolic content (6.0–44 mmol Ar–OH).^23^ Recent studies have identified nonphenolic sulfur-containing and nitrogen-containing moieties as important contributors to the EDC of DOM.^35,42,43^ Yet, the characteristics of DOM and DBC differ considerably. Nitrogen species in DOM are primarily associated with microbially derived amino acids and peptides, whereas those in DBC are typically present in more condensed forms, such as pyrrolic structures.^11,44,45^ Currently, both molecular and functional group-level insights into these nonphenolic EDC contributors in DBC remain limited due to challenges in integrating heterogeneous data sets, including functional group profiles alongside detailed molecular features.

This study examined EDC, molecular characteristics, and functional groups of DBC from nitrogen-rich biochar across sequential leaching cycles. By integrating two-dimensional correlation spectroscopy (2D-COS), Fourier-transform infrared spectroscopy (FTIR), Fourier-transform ion cyclotron resonance mass spectrometry (FTICR-MS), tandem mass spectrometry (MS/MS), and Spearman correlation analysis, we aim to (1) track the temporal changes in EDC, optical properties, molecular features, and functional groups of DBC during leaching process; (2) elucidate the dynamic evolution of DBC molecules, functional groups, and EDC-related compounds throughout sequential leaching cycles; and (3) identify key molecular features and functional groups governing the EDC changes of different DBC components. These findings can enhance our understanding of the long-term environmental impacts of pyrogenic carbon.

## 2 Materials and Methods

### 2.1 Dissolved Black Carbon Collection

Food waste digestate from O•PARK1 (Hong Kong) was selected as a nitrogen-rich feedstock due to its high protein content.^9,46^ Wildfire events similarly generated nitrogen-enriched DBC through heating soil rich in proteins under oxygen-limited conditions.^11^ Thus, using protein-rich food waste digestate as a precursor for nitrogen-rich pyrogenic carbon provides a relevant model to investigate the molecular composition and electron-donating capacity of DBC from nitrogen-rich biochar applications and natural wildfires. The food waste digestate was oven-dried, crushed to ∼ 1 mm particle size, and pyrolyzed in a tube furnace under N_2_ gas. Pyrolysis was conducted at 350, 450, and 550 °C, respectively, with a heating rate of 10 °C · min^–1^ and 2 h holding time for biochar production. These temperatures were common for both biochar production and wildfire-induced pyrogenic carbon formation.^11,47,48^ To simulate acidic precipitation, 10 g of biochar was leached in 1 L of pH 5 solution (adjusted with 60:40 H_2_SO_4_/HNO_3_) under stirring (110–130 rpm, 25 ± 2 °C, dark), following the U.S. EPA Synthetic Precipitation Leaching Procedure.^49^ Preliminary tests indicated rapid initial DBC release that gradually diminished over 30 days (Table S1), consistent with previous studies.^6,28^ Accordingly, a 30-day, five-cycle leaching design, with an initial 2-day extraction followed by four 7-day intervals, was adopted to represent typical soil leaching processes after irrigation or intermittent rainfall. After the initial 2-day leaching, the solution was filtered through a 0.45 μm pore-size membrane prerinsed with ultrapure water.^50^ Residual biochar was rinsed and subjected to four additional 7-day leaching cycles to simulate intermittent rainfall. Leachates were labeled by pyrolysis temperature and leaching duration (e.g., DBC350–2d, DBC350–9d, etc.) and stored at 4 °C in the dark until characterized. Ultrapure water treated under the same conditions served as blank controls.

### 2.2 Electron Donating Capacity and Spectroscopic Characterization

EDC quantifies electron transfer from DOM to an oxidant under controlled pH and reduction potential.^51^ In this study, EDC of DBC was measured according to an established method using the radical cation of 2,2’-azino-bis­(3-ethylbenzthiazoline-6-sulfonate) (ABTS^•+^) as the oxidant.^52^ The ABTS^•+^ solution was prepared by reacting 0.26 mM sodium hypochlorite with 1 mM ABTS, achieving 52% oxidation of ABTS to ABTS^•+^. EDC (mmol_e–_ · g_C_^–1^) was calculated from the reduction of ABTS^•+^ after 15 min of reaction with DBC samples:^52^

$$EDC=\frac{A_{blank}-A_{sample}}{l\cdot \varepsilon_{{\mathrm{ABTS}}^{•+}}}\cdot \frac{1}{c_{\mathrm{DOC}}}$$

where *A*_*blank*_ and *A*_*sample*_ are absorbances of ABTS^•+^ solutions without and with DBC at 728 nm, respectively; *l* is the optical path length (cm); *ε*_ABTS^•+^_ is the molar absorption coefficient of ABTS^•+^ (14,000 M^–1^cm^–1^), *c*_DOC_ (mg·L^–1^) is the DOC concentration of each DBC sample. All DBC samples showed no UV absorbance at 728 nm and did not interfere with the EDC tests.

Specific ultraviolet absorbance at 254 nm (SUVA_254_, L·mg^–1^·m^–1^) was calculated to assess DBC aromaticity,^53^ which was reported to well correlate with EDC of DOM.^20^ The humification index (HIX) was calculated to quantify DBC’s humification level.^37^ Three-dimensional excitation–emission matrix (EEM) fluorescence spectroscopy, combined with Parallel Factor Analysis (PARAFAC), identified DBC components across leaching cycles and pyrolysis temperatures.^54^ FTIR spectra of freeze-dried DBC samples (4000–400 cm^–1^, 4 cm^–1^ resolution) were collected to identify functional groups. X-ray photoelectron spectroscopy (XPS; Thermo Fisher Scientific, USA) with Al Kα radiation was used to determine the nitrogen and carbon chemical states of freeze-dried DBC samples. Solid-state ^13^C nuclear magnetic resonance (NMR) analysis was performed via the Bruker Ascend 600 M spectrometer to identify the chemical structures of the freeze-dried DBC samples following previous studies.^45,55,56^ Details for SUVA_254_, EEM, FTIR, XPS, and NMR measurements, along with data preprocessing procedures, are provided in Note S1.1.

### 2.3 Extraction and FTICR-MS Measurement

DBC was extracted and analyzed via FTICR-MS following our previous studies to identify molecular features.^9,37^ Molecular formulas were assigned to ion peaks with a signal-to-noise ratio > 4 and mass error < 1 ppm, using the constraints ^12^C_1–60_, ^1^H_1–120_, ^16^O_1–50_, ^14^N_0–3_, ^32^S_0–2_. Refinement of molecular assignments followed the established method.^9^ Intensity-weighted molecular parameters, including molecular weight (MW_w_), double-bond equivalents (DBE_w_), elemental ratios (H/C_w_, O/C_w_ H/N_w_ and O/N_w_), modified aromaticity index (AI_mod,w_), and normal oxidation state of carbon (NOSC) were calculated to reflect the molecular variations of DBC.^57,58^ Van Krevelen diagrams classified the DBC formulas into compositional categories: 1. aliphatics (1.5 ≤ H/C ≤ 2, O/C < 0.9, N = 0) and peptides (1.5 ≤ H/C ≤ 2, O/C < 0.9, N > 0), 2. highly unsaturated structures with low-oxygen (HUSLO, AI_mod_ < 0.5, H/C < 1.5, O/C < 0.5), 3. highly unsaturated structures with high-oxygen (HUSHO, AI_mod_ < 0.5, H/C < 1.5, 0.5 ≤ O/C ≤ 0.9), 4. aromatic structures (AS, 0.5 < AI_mod_ ≤ 0.67), 5. condensed aromatic structures (CAS, AI_mod_ > 0.67), 6. sugars (O/C > 0.9).^59^ Detailed DBC extraction procedures, FTICR-MS instrument settings and molecular parameter calculation methods are provided in Note S1.2.

### 2.4 Generalized and Hetero 2D–COS Analyses

To investigate the sequential changes of DBC molecules and functional groups during leaching, generalized 2D-FT-ICR MS-COS and 2D-FTIR-COS analyses were conducted following the previous studies.^60–63^ For the 2D-FT-ICR MS-COS analysis, molecular formulas in DBC samples from different leaching cycles were combined into a unique list after removing duplicates and excluding molecular peaks exceeding the mean peak intensity by more than two standard deviations. The formulas were visualized via 2D-COS maps drawing using H/C, O/C, H/N, and O/N ratios (*x*-axis), with the normalized molecular intensity for each ratio as *y*-axis and leaching time as the external perturbation.^60,61^ For the 2D-FTIR-COS analysis, the maps were drawn by selecting FTIR wavenumbers as *x*-axis and FTIR absorbance for each wavenumber as *y*-axis, with leaching time as the external perturbation.^62,63^ To see which functional groups were correlated with specific DBC molecules, hetero 2D-COS analysis was conducted between normalized FTICR-MS molecular intensities and FTIR absorbances.^61,64^

### 2.5 Spearman’s Rank Correlation Analysis

Spearman’s correlation analysis was conducted to identify molecular features potentially influencing EDC, by correlating normalized FTICR-MS peak intensities to EDC values of DBC samples.^37,38^ DBC molecular formulas related to EDC were identified based on Spearman’s *r* ≥ 0.90 and *p* < 0.05 (Student’s *t*-test).^37^ Dynamic changes of the EDC-related molecules during leaching were further analyzed via the generalized 2D-COS analysis on their H/C, O/C, H/N, and O/N ratios. To see associations of EDC-related molecules with functional groups, hetero 2D-COS analysis was conducted between their normalized FTICR-MS molecular intensities and FTIR absorbances.

### 2.6 Tandem Mass Spectrometry Analysis

Tandem mass spectrometry (MS/MS) analysis was performed using an Orbitrap IQ-X Tribrid MS coupled with a Dionex UltiMate 3000 UHPLC system (Thermo Fisher Scientific, Waltham, MA, USA) to characterize potential DBC molecular structures associated with EDC. To minimize interferences during MS analysis, chromatographic separation was performed using an ACQUITY UPLC BEH C18 column (2.1 × 100 mm, 1.7 μm; Waters) with 0.1% formic acid in water (A) and 100% acetonitrile (B) as mobile phases. The LC gradient started at 5% B (0–1 min), increased linearly to 95% B (1–5 min), maintained until 7 min, then re-equilibrated to 5% B (7.1–10 min). Parent ions were fragmented via higher-energy collisional dissociation, and the resulting neutral losses were used to inform structural analysis. Mass parameters were selected based on previously established guidelines.^65^ Comprehensive procedures and instrument parameter settings for MS/MS analysis are described in Note S1.3.

## 3 Results and Discussion

### 3.1 Electron Donating Capacity and Optical Characteristics

The EDC values in the first leaching cycle (2 d) ranged from 0.26 to 1.63 mmol_e–_ g_C_^–1^ (Figure 1a and Table S1), consistent with reported values for DOM and DBC from biomass-derived charcoal using the ABST^·+^ as the oxidant.^20,25^ Over 30 d, EDC increased by factors of 12 (DBC350), 6 (DBC450), and 5 (DBC550), respectively. By day 16, the EDC value of DBC350 exceeded that of Suwannee River II Standard Fulvic Acid (5.14 mmol_e–_·g_C_^–1^)^52^ and those reported for natural and wastewater-derived DOM.^20^ The values of SUVA_254_ increased in parallel with EDC over successive leaching cycles (Figure 1a), indicating progressive enrichment of aromatic and/or heteroaromatic structures. In contrast, the HIX increased for DBC350 and DBC450 but decreased for DBC550 between days 2 and 9. This divergence between HIX reflected its limited sensitivity in capturing DBC humification, possibly due to its narrow fluorescence detection range. To address this limitation, a three-component EEM-PARAFAC model identified terrestrial humic-like/N-heterocyclic (C1), soluble microbial byproduct-like (C2), and microbial humic-like (C3) fluorophores in all DBC samples (Figure S1). The relative abundance of C1 and C3 increased, whereas that of C2 decreased with successive leaching cycles (Figure 1c), indicating enhanced humification levels over time. These increasing aromaticity and humification degrees over leaching time indicated growing hydrophobicity,^66^ which may account for their persistence compared to hydrophilic counterparts during wash-off from the biochar surface.^6^ These aromatic DBC components imply a long-term impact on aquatic ecosystems,^67^ requiring further attention.

**Figure 1:**
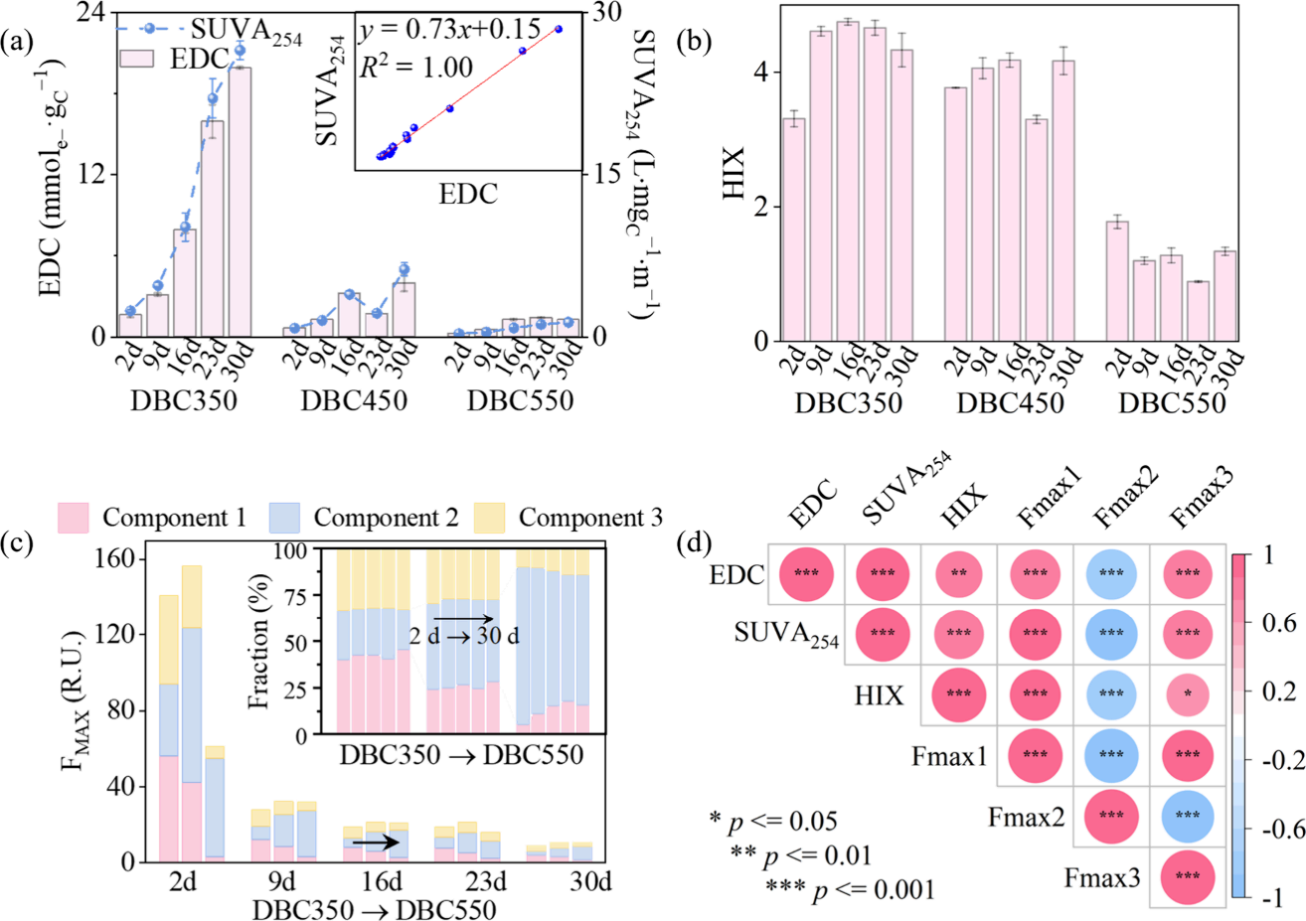
(a) EDC and SUVA_254_ values with Pearson’s correlations displayed in the illustration; (b) HIX of DBC samples; (c) intensities and intensity percentages of three EEM-PARAFAC components; (d) Spearman’s correlation matrix for DBC parameters across pyrolysis temperatures and leaching times (*n* = 15, Fmax1–Fmax3 represent the relative abundance of C1–C3), with Spearman’s *r* and *p* values in Table S2.

Across all leaching cycles, DBC350 consistently showed higher EDC and SUVA_254_ values than DBC450 and DBC550 (Figure 1a, DBC350 > DBC450 > DBC550). A strong positive correlation between SUVA_254_ and EDC (Pearson’s *R* ^2^ = 1.00) was observed, aligning with prior observations in humic substances, DOM, and DBC from pinecone-derived biochar.^20,30,68^ This suggests that EDC variation is closely linked to aromaticity across pyrolysis temperatures and over leaching time. The slope of the EDC–SUVA_254_ regression (*k* = 0.73) was consistent among all DBC types (Figure 1a), implying a uniform EDC increase per unit aromaticity.^53^ This slope lies between those for natural DOM (*k* = 0.34) and wastewater DOM (*k* = 2.50),^20^ indicating an intermediate density of electron-donating functional groups per unit aromaticity. Furthermore, DBC350 exhibited higher levels of humic fractions (C1 + C3) and HIX, potentially contributing to its higher EDC. Spearman’s rank correlation analysis confirmed that EDC was positively associated with SUVA_254_, HIX, and the relative abundance of C1 (Fmax1) and C3 (Fmax3), but negatively with that of C2 (Fmax2) (*p* < 0.05, *n* = 15; Figure 1d), reinforcing the pivotal role of aromatic humic-like/N-heterocyclic components in driving the EDC changes.

### 3.2 Molecular Feature Changes and Correlation with Electron Donating Capacity

Van Krevelen diagrams revealed a wide molecular diversity in the DBC samples (2084–7,370 detected formulas; Figure S2a), indicating complex leaching behavior. With increasing leaching cycles, the CAS number percentages and AI_mod,w_ generally increased in DBC350 and DBC450 (Figure S2b and Table S3), aligning with the elevated aromaticity and EDC. In contrast, there was an irregularity in DBC550 that may be attributed to a higher abundance of aliphatic compounds (Figure S2b). The (DBE-O)/C vs NOSC plots (Figure S2c,d) showed that DBC molecules across all temperatures were mainly unsaturated-reduced (37.9%–65.2%), similar to water-soluble organic carbon from biomass pyrolysis smoke,^61^ showing a high potential to be oxidized. To assess molecular turnover during leaching, we compared unique and shared molecules across leaching cycles (Figure 2a,b). Unique compounds, particularly on day 2, were widely distributed across chemical space (Figure 2a and Figure S3a–e). In contrast, shared molecules clustered within the HUSLO, HUSHO, and AS regions (Figure 2b) and were dominated by unsaturated, reduced structures (Figure S3f), potentially due to selective retention of hydrophobic species by biochar. Notably, distinct patterns emerged for nitrogen-bearing and sulfur-bearing species (Figure S4). In DBC350 and DBC450, the molecules uniquely released at different leaching cycles contained higher proportions ofsulfur-containing CHOS and CHONS species than the shared molecules, consistent with their lower aromaticity and greater mobility (Table S4). In contrast, the shared fractions across all leaching cycles for both DBC350 and DBC450 were dominated by nitrogen-containing CHON species that were likely more aromatic and less oxidized (Table S5). In DBC550, CHO and CHON were mainly shared, while CHONS appeared only among unique molecules and declined from 24.1% to 3.4% over 30-day leaching. These patterns suggest that sulfur-bearing compounds may be preferentially mobilized, whereas more aromatic CHO and CHON species were retained through π–π interactions with biochar and released more slowly, potentially exerting longer-term effects in aquatic systems.

**Figure 2:**
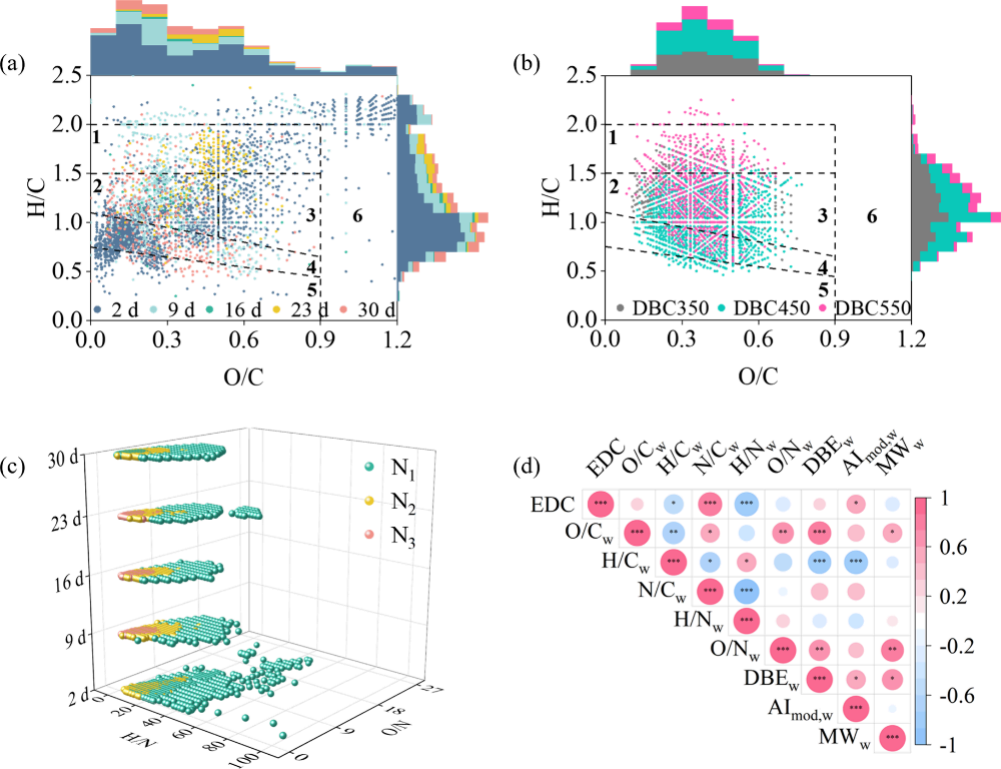
Van Krevelen diagrams and corresponding molecular feature histograms for (a) DBC350 molecules unique to each leaching cycle and (b) shared molecules across leaching cycles in DBC350, DBC450, and DBC550; (c) H/N and O/N ratios of N-bearing molecules in DBC350; (d) Spearman’s correlation matrix of EDC and DBC molecular parameters (*n* = 15; *r* and *p* values are provided in Table S6). Regions in the van Krevelen diagram were divided into: 1. aliphatics and peptides, 2. HUSLO, 3. HUSHO, 4. AS, 5. CAS, 6. sugars.

To assess nitrogen speciation, O/N and H/N ratios were used in place of traditional O/C and H/C ratios (Figure 2c). These metrics better reflect nitrogen oxidation and saturation.^48^ Nitrogen-rich molecules (N2 and N3, with suffixes showing nitrogen element numbers) exhibited lower O/N and H/N ratios than N1 species (Figure 2c and Figure S5a,b), which may reflect a higher nitrogen unsaturated degree related to N-heterocyclic compounds.^48,69^ Higher nitrogen-rich molecules (i.e., N2 and N3) percentages in DBC350 across different leaching cycles (Figure S5c,d) potentially implied more unsaturated N-heterocyclic compounds. Over time, average O/N, H/N, O/C, and H/C ratios all declined (Figure S6a), indicating synchronized decreases in nitrogen and carbon oxidation and saturation. The synchronized declines in average H/N and H/C ratios over leaching cycles suggested increasing unsaturated nitrogen structures, while the declines in average O/N and O/C ratios indicated a loss of oxygen-rich groups (e.g., carboxyls). Positive correlations between H/N and H/C (*R* ^2^ = 0.031–0.293, *p* < 0.05) and between O/N and O/C (*R* ^2^ = 0.271–0.368, *p* < 0.05; Figures S7 and S8) in each DBC sample explained the synchronization. Although the relationships between H/C and H/N, and between O/C and O/N, were statistically significant (*p* < 0.05), their low *R* ^2^ values suggested that H/C and O/C explained only a small portion (3.1%–36.8%) of the variability in H/N and O/N. The differences in the unsaturation levels of carbon and nitrogen warrant further investigation. Despite similar H/N ratios, DBC450 exhibited higher O/N ratios than DBC350 (Figure S6a). Venn analysis of day-2 leachates revealed a greater abundance of oxygen-rich compounds (e.g., N1O8–N1O14, N2O11–N2O13) in DBC450 than DBC350 (Figure S6b), reflecting a higher content of oxygenated groups in DBC450 and potentially reducing its EDC.^70^

Spearman’s correlation analysis revealed a strong association between EDC and molecular parameters, particularly H/N_w_, AI_mod,w_, and N/C_w_ (Figure 2d). The EDC correlated positively with N/C_w_ (*p* < 0.001, *r* = 0.83; Table S6), similar to an earlier study that a strong negative correlation (*p* < 0.1, *r* ≥ – 0.84) existed between C/N and antioxidant capacity in humic-like substances.^42^ This indicated that nitrogenous moieties potentially influenced EDC. Among parameters, H/N_w_ exhibited the strongest negative correlation with EDC (*p* < 0.001, *r* = – 0.85), implying that unsaturated N-heterocyclic structures (e.g., pyrrole derivatives^70–72^ ) may drive EDC via conjugation or induction.

### 3.3 Leaching Sequences of DBC Molecules

A substantial portion of DBC molecules (32%–73%) was shared across leaching cycles (Figure 2b), complicating the determination of their leaching orders. Hence, 2D-FTICR-MS-COS analysis was employed to elucidate sequential leaching patterns based on the changes in relative abundance of DBC molecules (Figure 3, Figures S9 and S10, and Table S7). Among the samples, DBC550 showed stronger synchronous signals than DBC350 and DBC450. This was consistent with its lower aromaticity (Table S3), which reduced π-π interactions, particularly π–π electron donor–acceptor coupling interactions, with biochar. For DBC350, synchronous maps revealed positive autopeaks in the high H/C (1.80–2.30) and O/C (0.55–1.20) regions, while asynchronous maps indicated negative cross-signals in the *v*1*/v*2 of 1.80–2.10/2.10–2.30 for H/C and 0.55–1.05/1.05–1.20 for O/C (Figure 3a). According to Noda’s rules,^60–62^ the leaching sequence followed: 2.10–2.30 → 1.80–2.10 for H/C and 1.05–1.20 → 0.55–1.05 for O/C, indicating that highly saturated and oxidized compounds (e.g., sugars) leached earlier than less oxidized aliphatic and peptides. Nitrogen-bearing molecules in DBC350 also displayed a preferential release of saturated and highly oxidized structures, characterized by high H/N and O/N ratios (Figure S9a and Table S7). The leaching patterns were further analyzed across different *m*/*z* ranges (100–275, 275–450, 450–620, and 625–800) (Figure 3b). For DBC350, negative signals (both synchronous and asynchronous) in the *v*1/*v*2 of *m*/*z* 245–275/150–235 and 430–450/275–420 revealed sequential leaching patterns of *m*/*z* 245–275 → 150–235 and 430–450 → 275–420, indicating the prioritized release of larger molecules.

**Figure 3:**
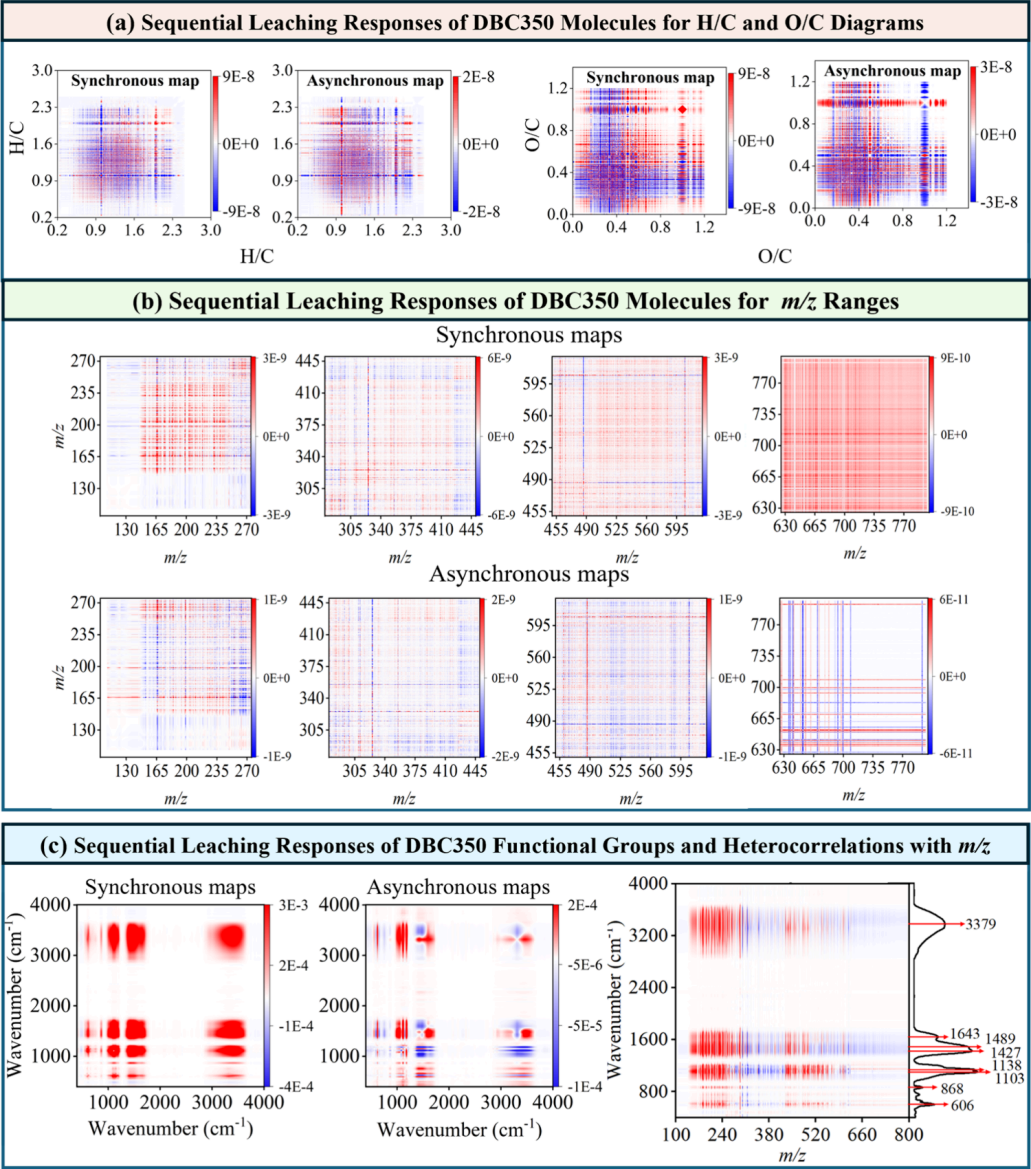
2D-FTICR-MS-COS maps of DBC350 molecules across different leaching cycles based on (a) H/C and O/C ratios, and (b) *m*/*z*; (c) 2D-FTIR-COS maps and the distribution of 2D-FT-ICR MS/FTIR-COS heterocorrelations of DBC350 across different leaching cycles.

Similar trends were observed in DBC450, where highly saturated (H/C: 2.00–2.20), oxidized (O/C: 0.55–0.70), and large (*m*/*z*: 500–625) molecules leached preferentially over unsaturated (H/C: 1.15–1.35), less oxidized (O/C: 0.45–0.55), and smaller (*m*/*z*: 460–500) counterparts (Figures S9 and S10, Table S7, and Note S2.1). This preferential leaching of larger, oxygen-rich species likely reflects stronger hydrogen bonding with water. DBC550 showed complex leaching behavior with no clear H/C trends (Figures S9 and S10 and Table S7), likely due to its lower aromaticity (Table S3) and greater molecular variability (Figure S2a). Unlike DBC350, DBC450 and DBC550 showed rapid leaching of unsaturated nitrogen structures (H/N: 23.0–39.0 → 2.5–7.5 → 39.0–43.0 for DBC450; H/N: 3.0–7.0 → 15.0–23.0 → 31.0–38.5 for DBC550) (Table S7), likely owing to their higher contents of oxygenated functional groups (Figure S6b), which enhanced hydrogen bonding with water. However, 2D-COS analysis of DBC molecules only provided a limited resolution for low-ratio regions (e.g., HUSLO molecules with H/C < 1.5, O/C < 0.5) due to signal overlap. Further refinement of 2D-COS signals will be necessary to reveal the leaching dynamics of unresolved HUSLO species.

### 3.4 Leaching Sequence of Functional Groups and Heterocorrelations with Molecules

2D-FTIR-COS analysis was performed to identify the dynamic leaching behavior of functional groups (Figure 3c and Figure S11), with detailed functional group analysis (Note S2.2) and valid signals in synchronous/asynchronous maps (Table S9). Following Noda’s rules,^62^ oxygen-rich aliphatic C–OH, carbohydrate/polysaccharides C–O, and amide/carboxylic C = O (1138, 1049, 1643 cm^–1^) dominated early stage leaching for DBC350, whereas aromatic C = C, condensed C–H, and heterocyclic N–H (1489, 1427, 868, 750, 3379 cm^–1^) structures became more prominent afterward (Table S9). In DBC450, heteroaromatic N–H structures (3317, 750 cm^–1^) leached faster than oxygen-rich carbohydrate/aromatic ether C–O, secondary alcoholic C–O, aliphatic C–OH, and carboxylic O–H (1041, 1095, 1146, 1200 cm^–1^), while aromatic and condensed C = C structures (868, 1481, 1431 cm^–1^) were released in the later cycles (Figure S11b and Table S9). The alternative releases of aromatic and aliphatic structures observed in DBC450 were also evident in DBC550 (Figure S11c), likely moderating their EDC fluctuations compared to DBC350 (Figure 1a). Overall, the late release of aromatic/condensed structures in DBC350, as well as the alternating releases of heterocyclic structures in DBC450 and DBC550, closely aligned with their 2D-FTIRMS-COS results.

Heterogeneous correlations between FTIR absorbances and normalized FTICR-MS peak intensities could link functional groups to specific DBC molecules (Figure 3c and Figures 13a and S14a). Positive correlations indicated consistent leaching behavior, where stronger heterocorrelation intensity suggested greater functional group contributions to specific DBC molecules (Note S2.3). In DBC350, aromatic and nitrogen-rich groups (i.e., 868, 1427, 1489, 1643, and 3379 cm^–1^) mainly exhibited positive correlations with the midweight compounds (*m*/*z* 275–400, 41.4%–47.2%), while oxygen-rich groups (i.e., 1103, and 1138 cm^–1^) predominantly positively correlated with higher molecular weights (*m*/*z* 450–625, 34.3%–38.4%) (Figure S12). Likewise, more macromolecules were linked to oxygen-rich functional groups than aromatic structures in DBC450 and DBC550 (Figures S13 and S14 and Note S2.3), aligning with the positive correlations of MW_w_ with both O/N_w_ and O/C_w_ (Figure 2d).

Overall, highly oxidized large molecules (*m*/*z* 450–625) containing carboxylic, alcoholic, polysaccharide-like, amide, and aromatic ether groups could be leached more rapidly in DBC350 and DBC450 due to more significant formation of hydrogen bonding with water. Aromatic structures showing smaller molecular sizes (*m*/*z* 275–450), potentially stemming from condensation of proteins,^9^ were leached in the later cycles (Figures S12b and S13b), which may serve as crucial drivers of the elevated EDC during sequential leaching. These smaller aromatic molecules may result in treatment challenges during conventional water treatment,^66^ warranting further attention in the environment. In comparison, DBC550 exhibited more complex leaching dynamics (Figure S14b), potentially caused by the higher leachability of aromatic C = C groups in HUSLO molecules.

### 3.5 Linking EDC to Specific DBC Molecules and Functional Groups

Spearman correlation analysis (*p* = 0.05) revealed nitrogen-bearing formulas constituted 75%, 76%, and 61% of EDC-related molecules in DBC350, DBC450, and DBC550, respectively, with N1 species predominating over N2/N3 (Figure 4a). This aligned with the strong correlation between EDC and N/C_w_ (Figure 2d), identifying nitrogen-rich moieties as the primary EDC drivers. The DBC350 and DBC450 shared 161 EDC-related formulas, while DBC550 exhibited only 30 overlapping formulas with DBC450 and 11 with DBC350, reflecting significant molecular compositional differences. A MANOVA (*p* < 0.0001) on molecular characteristics (H/N, O/N, AI_mod_, and MW)^38^ confirmed that EDC-related molecules in DBC350, DBC450, and DBC550 belonged to statistically distinct families. Despite this, EDC-correlated formulas across different pyrolysis temperatures were all predominantly enriched in HUSLO regions (O/C < 0.5 and H/C < 1.5; Figure 4a). A focused analysis of 2D-FT-ICR MS-COS maps for EDC-related molecules (Note S2.4, Figures S15–S17, and Table S10) provided clearer insights into their leaching dynamics, which were otherwise difficult to discern in broader maps encompassing all DBC molecules. It is noted that highly unsaturated and aromatic EDC-related compounds (low H/C, H/N) leached more slowly across DBC pyrolysis temperatures, potentially underscoring their critical role in promoting EDC during extended extraction cycles. Yet, *m*/*z*, O/N, and O/C exhibited more complicated changing sequences over increasing leaching time, which deviated from overall trends. This was possibly caused by the low oxygen contents of the EDC-related molecules, which reduced their hydrophilicity, making their leaching sequences less sensitive to changes in oxygen content.

**Figure 4:**
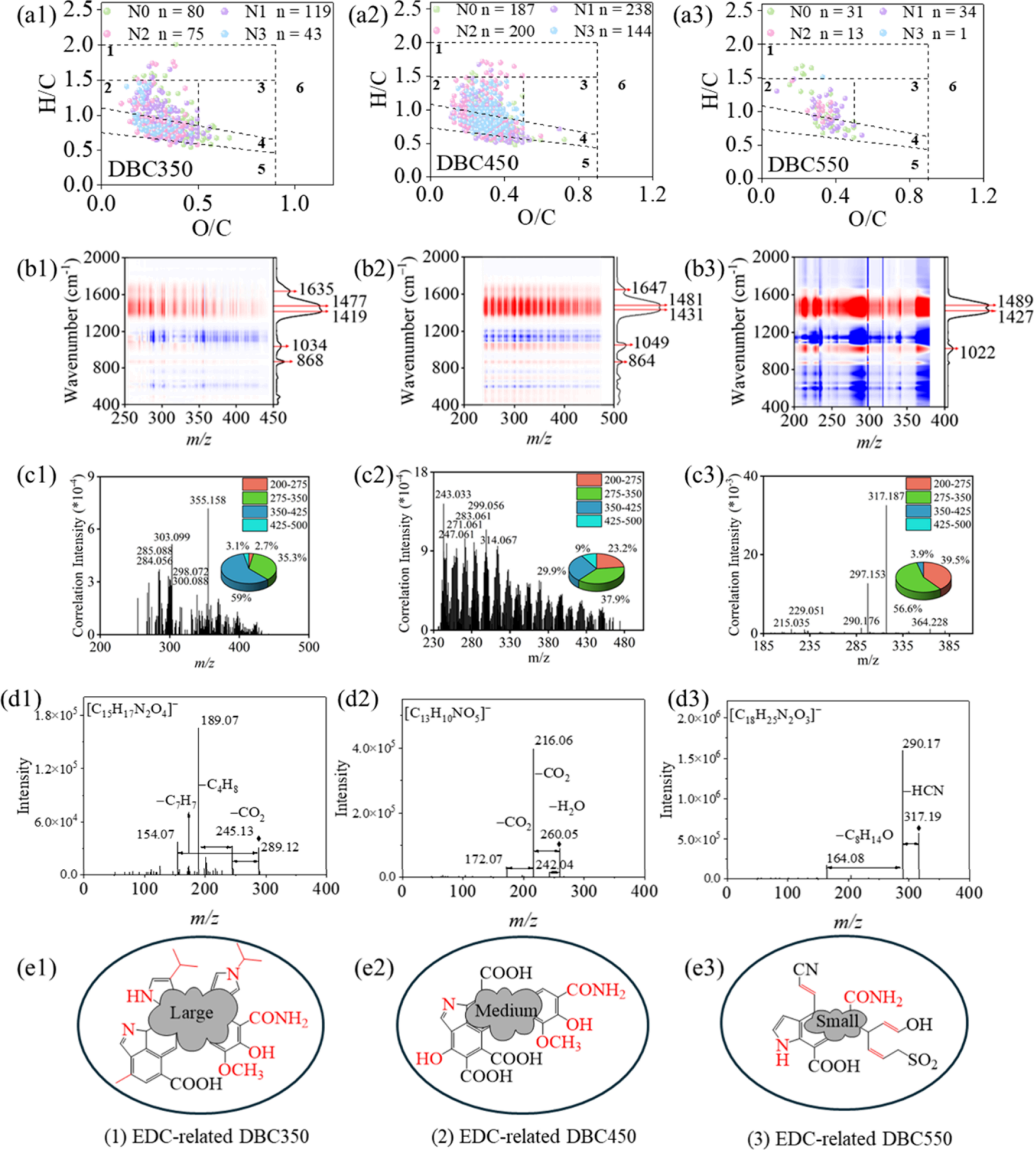
(a1)–(a3) Van Krevelen plots of EDC-related formulas in DBC350–DBC550, with regions categorized as 1. aliphatics and peptides, 2. HUSLO, 3. HUSHO, 4. AS, 5. CAS, 6. sugars; (b1)–(b3) 2D-FT-ICR MS/FTIR-COS heterocorrelation distributions of EDC-related molecules; (c1)–(c3) distribution and *m*/*z* range percentages of positive heterocorrelations between normalized intensities of EDC-related molecules and absorbances at ∼ 1482 cm^–1^; (d1)–(d3) MS/MS fragmentation patterns of the most intense nitrogen-bearing EDC-related molecules; (e1)–(e3) schematic illustrations of functional group features in EDC-related molecules, with potential electron-donating groups indicated in red.

To identify functional groups in EDC-related molecules, hetero 2D-COS was performed between their normalized FTICR-MS abundances and FTIR absorbances within the 2000–400 cm^–1^ region (Figure 4b). Strong positive heterocorrelations indicated that specific functional groups contributed significantly to the composition of EDC-related DBC molecules.^61^ The EDC-related molecules in DBC350 and DBC450 showed strong associations with FTIR peaks at ∼ 866 cm^–1^ (condensed aromatic C–H), ∼ 1042 cm^–1^ (aromatic ether C–O), ∼ 1425 cm^–1^ (lignin C = C/pyrrolic C–H/carboxylic O–H), ∼ 1479 cm^–1^ (aromatic/heteroaromatic C = C), and ∼ 1641 cm^–1^ (aromatic C = C/carboxyl C–O/amide C = O). The DBC550 exhibited significant correlations at 1022 cm^–1^ (polysaccharide-like C–O), 1427 cm^–1^, and 1489 cm^–1^. The shift from ∼ 1042 to 1022 cm^–1^ in EDC-related DBC550 likely resulted from thermal decomposition of oxygen-rich groups such as aromatic ether at 450–550 °C.^9^ The diminished heterocorrelations at 1641 cm^–1^ in EDC-related DBC550 species can be assigned to the destruction of carboxyl C–O and/or amide C = O functionalities.^55,73^ The FTIR absorbances at 1050–1200 cm^–1^ correlated negatively with EDC-related molecules, suggesting that these oxygen- and sulfur-rich functional groups may be electron-withdrawing. Elevated negative heterocorrelation intensities in this 1050–1200 cm^–1^ range for DBC550 indicated the increased abundance of such oxygen- and sulfur-rich functionalities. Notably, the strongest correlations for EDC-related DBC350, DBC450, and DBC550 molecules were observed at ∼ 1482 cm^–1^, suggesting that (hetero)­aromatic systems could be reliable determinants of EDC (Figure 4b). This pattern aligned with positive correlations of EDC with both AI_mod,w_ and SUVA_254_, as well as negative correlations with both H/C_w_ and H/N_w_ (Figures 1d and 2d). These (hetero)­aromatic structures contributed to the consistent slope in the linear relationships between EDC and SUVA_254_ across DBC samples from different leaching cycles and temperatures (Figure 1a).

Molecular distribution analysis (Figure 4c) showed that in DBC350, 59.0% of EDC-related (hetero) aromatic C = C groups (1477 cm^–1^) were correlated to molecules in the *m*/*z* 350–425, followed by 35.3% in *m*/*z* 275–350. In DBC450, these proportions decreased to 29.9% and 37.9%, respectively, with an increase in the lower 200–275 *m*/*z* range (23.2%). This shift indicated larger (hetero)­aromatic structures in DBC350, potentially contributing to its elevated EDC. In DBC550, most (hetero) aromatic C = C groups were linked to lower *m*/*z* 275–350 (56.6%) and 200–275 (39.5%), indicating fragmentation of aromatic structures at higher temperatures.^9^ Heterocorrelation maps of EDC-related molecular distributions for functional groups at ∼ 866, ∼ 1425, and ∼ 1641 cm^–1^ closely resembled those of the (hetero)­aromatic group at ∼ 1482 cm^–1^ in both heterocorrelation intensity distributions and molecular number percentages (Figures S18–S20). This similarity suggests that these groups may be structurally integrated within (hetero)­aromatic frameworks. In contrast, the aromatic ether (∼1042 cm^–1^) and polysaccharide-like (1022 cm^–1^) groups exhibited distinct heterocorrelation patterns, suggesting that these groups may be associated with different molecular entities. A single FTIR band may correspond to multiple functional groups. Complementary techniques, such as MS/MS fragmentation analysis, are crucial for further characterizing these structures involved in EDC.

### 3.6 Molecular Structures Driving Electron Donating Capacity

The MS/MS fragmentation patterns were further analyzed to identify molecular structures contributing to EDC. Considering the predominance (61%–76%) of nitrogen-bearing molecules in driving EDC, the MS/MS fragmentation patterns of the most intense nitrogen-bearing EDC-related molecules in DBC350, DBC450, and DBC550 were examined (Figure 4d). In DBC350 and DBC450, no nitrogen-containing neutral losses were observed, suggesting that nitrogen was stably incorporated into polyaromatic cores.^70^ In contrast, DBC550 exhibited a neutral loss of HCN from [C_18_H_25_N_2_O_3_]^−^, likely due to partial destruction of heteroaromatic nitrogen to hydrocyanic group at higher pyrolysis temperatures.^74,75^ Additional analysis of nitrogen-bearing EDC-related DBC350 molecules with high relative intensities (Figures S21–S23) revealed neutral losses such as C_4_H_8_N ([C_14_H_23_N_2_O_4_]^−^) and C_9_H_6_N for ([C_16_H_13_N_2_O_4_]^−^), indicating the presence of less stable unsaturated or heterocyclic nitrogen functionalities. Those nitrogen-bearing neutral losses were absent in DBC450, suggesting enhanced aromatic stabilization (Table S11). The absence of oxygen within these N-containing neutral losses suggests minimal contributions from O/N-substituted groups (e.g., nitryls), which readily fragment.^70,76^ Typical primary amide-related neutral losses (e.g., NH_3_, HNCO) were absent across EDC-related DBC molecules. This may be attributed to the substantial conversion of amide-N into heterocyclic N forms (e.g., pyrrolic, pyridinic, indolic) during pyrolysis.^45,70,77^

Previous studies have reported that amide functionalities became structurally insignificant in nitrogen-rich pyrogenic carbon formed above 350 °C,^11,44,45^ and heteroaromatic N was typically assumed in DBC.^8^ Recent FTIR and NMR analyses of in DBC also attributed the 1635 cm^–1^ band primarily to aromatic-conjugated carboxyl groups rather than amides.^55^ XPS and NMR results supported this transformation (Note S2.5 and Figures S24 and S25), with peak shifts from amide-N (N 1s 399.9 eV) and amide C = O (C 1s 288.6 eV) in the precursor to pyrrolic-N (N 1s ∼ 400.3 eV) and carboxyl-type COOH (C 1s ∼ 289.0 eV) in DBC, along with markedly reduced COO/NC = O signals (167–184 ppm) in DBC350 compared with food waste digestate. The potential occurrence of pyrrolic-N aligned with previous reports,^44,45,70^ and likely enhanced EDC due to its delocalized lone pair and strong electron-donor behavior relative to pyridinic, graphitic, or pyridone-like N.^78^ Pyrrolic and pyridinic moieties have been reported to contribute significantly to the electron-exchange capacity of pyrogenic carbon and exhibit higher electron-shuttling efficiency than quinone/phenol pairs.^72^ Nevertheless, potential contributions from residual amide groups to the EDC-related ∼ 1642 cm^–1^ band cannot be entirely excluded due to complex fragmentation pathways, overlapping N 1s signals (proteins-N at ∼ 399.9 eV and pyrrolic-N at ∼ 400.3 eV), and residual COO/NC = O resonances in ^13^C NMR (167–184 ppm) (Figures S24 and S25).

No neutral losses of H_2_O or CO were observed in the most intense nitrogen-bearing DBC350 species, and such losses were rare among other high-intensity EDC-related molecules (Figures S21–S23), indicating limited contribution from phenolic structures.^79^ In contrast, CO_2_ neutral loss was consistently observed in DBC350 and DBC450 molecules (Figure 4d and Figures S21–S23), suggesting a high abundance of carboxylic acid functionalities.^70^ Compared with DBC350, nitrogen-bearing EDC-related molecules in DBC450 showed more frequent CO_2_ losses, consistent with their higher oxygen content (Figure S6). This enhanced carboxylation likely reduced their electron-donating capacity relative to DBC350. In DBC550, CO_2_ loss was absent in several EDC-related species, particularly those with low aromaticity, likely due to cracking and decarboxylation of (hetero)­aromatic carboxyl groups at elevated pyrolysis temperatures.^9^ Nevertheless, CO_2_ loss persisted in highly aromatic and unsaturated species such as [C_13_H_9_O_4_]^−^ (AI_mod_ = 0.64) and [C_11_H_11_O_4_]^−^ (AI_mod_ = 0.44), indicating that carboxylated (hetero)­aromatic structures remained a consistent contributor to EDC. Neutral losses of alkyl and unsaturated hydrocarbon fragments (e.g., CH_2_, C_2_H_2_, CH, C_7_H_7_) in DBC350 and DBC450 implied alkylated heteroaromatic structures in enhancing EDC.^80^ The detection of SO_2_ loss in DBC550 (e.g., [C_8_H_9_O_3_S]^−^) suggested the presence of sulfonic acid groups, which may largely suppress EDC due to its strong electron-withdrawing effect. By integrating the above analysis, we summarized the dominant EDC-contributing structures (Figure 4e). In DBC350 and DBC450, pyrogenic nitrogen was embedded in heteroaromatic cores such as pyrrolic forms, along with the coexisting carboxylic, amide, methoxy, alkyl, and phenolic groups. Larger molecular size in DBC350 compounds supported the increased electron-donating functionalities, i.e., pyrrolic groups. These groups could stabilize radicals during reducing ABST^·+^ through π-conjugation and hydrogen bonding,^78^ thereby enhancing EDC. More extensive carboxyl substitution in DBC450 decreased its EDC relative to DBC350. In DBC550, thermal degradation transformed these structures into aliphatic C–OH and – CN groups,^9^ thereby reducing EDC via loss of aromaticity and increased presence of electron-withdrawing groups (Figure 4b).

## 4 Environmental Implications

Understanding the molecular structures that drive the EDC changes of DBC is critical for predicting downstream impacts on aquatic systems, including photoactivity and byproduct formation.^21,22,81^ This study presents the first integrated molecular, functional group, and structural characterization of DBC from nitrogen-rich pyrogenic carbon during sequential leaching across intermediate rainfall events, with a particular focus on their EDC-related components. We observed a delayed and sustained release of smaller unsaturated and aromatic nitrogen-bearing structures that could significantly enhance EDC, underscoring the need for long-term monitoring.^67^ Notably, 61–76% of EDC-related molecules contained nitrogen, predominantly in (hetero)­aromatic structures with various functionalities, thus shifting the traditional focus from phenolic moieties associated with EDC.^23,30^ These structural insights have important implications for assessing the emerging formation of nitrogenous byproducts, such as halopyrroles,^82,83^ which are more toxic than their carbonaceous counterparts.^82^ Moreover, the enrichment of N-bearing heteroaromatic structures over leaching time can enhance the generation of reactive species upon photolysis, with long-term effects on the fate of organic contaminants and metals in water and soil.^8^ Collectively, these observations highlight the need to focus on N-bearing functionalities in DBC. Understanding these transformations can improve predictions of DBC behavior under intermittent rainfall and support more effective strategies for water quality monitoring and risk management following wildfires or biochar applications.

Methodologically, this study introduces a novel integrative framework combining multiple advanced analytical techniques to elucidate the structural features of EDC-related DBC molecules. In addition to traditional approaches that correlate bulk FTICR-MS and FTIR signals, our method targets EDC-specific molecular signatures. A focused hetero 2D-COS analysis, linking normalized FTICR-MS intensities of EDC-related molecules with FTIR absorbance, enhanced functional group resolution and enabled more accurate identification of EDC-driving functionalities. Integration with automated MS/MS fragmentation and XPS further revealed the detailed patterns of nitrogen incorporation. Beyond improving characterization of complex EDC-related mixtures, this framework may provide a versatile platform for identifying structurally ambiguous environmental contaminants, such as fluorophores. Although precise elucidation of molecular structures remains limited, our findings lay the groundwork for future research using advanced separation techniques and high-resolution spectroscopic tools to better resolve EDC-associated molecular structures.

## Supplementary Material



## Notes

The materials and methods encompass spectroscopy (UV, fluorescence, FTIR, XPS, and NMR); solid-phase extraction and mass spectrometry. The results and discussion focus on the identification of functional groups; sequential leaching responses of DBC molecules, EDC-related molecules, and their associated functional groups. Figures present spectral analysis; molecular feature contributions; 2D-COS maps and heterocorrelation distributions; fragmentation patterns. Tables provide a summary of DOC concentrations and spectral parameters; molecular parameters; Spearman’s *r*; detailed leaching sequences of DBC molecules, functional groups, EDC-related molecules (PDF)

## References

[1] IPCC. Global warming of 1.5 °C, an IPCC special report on the impacts of global warming of 1.5°C above pre-industrial levels and related global greenhouse gas emission pathways, in the context of strengthening the global response to the threat of climate change, sustainable development, and efforts to eradicate poverty; World Meteorological Organization: Geneva, Switzerland, 2018.

[2] Lehmann J. (2007). A Handful
of Carbon. Nature.

[3] Woolf D., Amonette J. E., Street-Perrott F. A., Lehmann J., Joseph S. (2010). Sustainable
Biochar to Mitigate Global Climate Change. Nat.
Commun..

[4] Han M., Zhang J., Zhang L., Wang Z. (2023). Effect of Biochar Addition
on Crop Yield, Water and Nitrogen Use Efficiency: A Meta-Analysis. J. Clean. Prod..

[5] He M., Xu Z., Hou D., Gao B., Cao X., Ok Y. S., Rinklebe J., Bolan N. S., Tsang D. C. W. (2022). Waste-Derived
Biochar for Water Pollution Control and Sustainable Development. Nat. Rev. Earth Environ..

[6] Zhang X., Sun Y., Zhang Q., Tian W., Khan E., Tsang D. C. W. (2024). Leaching
Characteristics of Nutrients in Food Waste Digestate-Derived Biochar. Bioresour. Technol..

[7] Ding Y., Watanabe A., Jaffé R. (2014). Dissolved
Black Nitrogen (DBN) in
Freshwater Environments. Org. Geochem..

[8] Lian F., Zhang Y., Gu S., Han Y., Cao X., Wang Z., Xing B. (2021). Photochemical Transformation
and
Catalytic Activity of Dissolved Black Nitrogen Released from Environmental
Black Carbon. Environ. Sci. Technol..

[9] Zhang X., Xu Z., Sun Y., Mohanty S. K., Lei H., Khan E., Tsang D. C. W. (2025). Implications of Pyrolytic Gas Dynamic Evolution on
Dissolved Black Carbon Formed During Production of Biochar from Nitrogen-Rich
Feedstock. Environ. Sci. Technol..

[10] Adam D. (2022). Tonga Volcano
Eruption Created Puzzling Ripples in Earth’s Atmosphere. Nature.

[11] Bahureksa W., Young R. B., McKenna A. M., Chen H., Thorn K. A., Rosario-Ortiz F. L., Borch T. (2022). Nitrogen Enrichment during Soil Organic
Matter Burning and Molecular Evidence of Maillard Reactions. Environ. Sci. Technol..

[12] Jones M. W., Coppola A. I., Santín C., Dittmar T., Jaffé R., Doerr S. H., Quine T. A. (2020). Fires Prime
Terrestrial Organic Carbon
for Riverine Export to the Global Oceans. Nat.
Commun..

[13] Xu Y., Wang X., Ou Q., Zhou Z., van der
Hoek J. P., Liu G. (2024). Appearance of Recalcitrant Dissolved
Black Carbon and Dissolved Organic Sulfur in River Waters Following
Wildfire Events. Environ. Sci. Technol..

[14] Hohner A. K., Rhoades C. C., Wilkerson P., Rosario-Ortiz F. L. (2019). Wildfires
Alter Forest Watersheds and Threaten Drinking Water Quality. Acc. Chem. Res..

[15] Liu Y., Wang M., Yin S., Xie L., Qu X., Fu H., Shi Q., Zhou F., Xu F., Tao S., Zhu D. (2022). Comparing Photoactivities of Dissolved
Organic Matter Released from
Rice Straw-Pyrolyzed Biochar and Composted Rice Straw. Environ. Sci. Technol..

[16] Wan D., Wang J., Dionysiou D. D., Kong Y., Yao W., Selvinsimpson S., Chen Y. (2021). Photogeneration of Reactive Species
from Biochar-Derived Dissolved Black Carbon for the Degradation of
Amine and Phenolic Pollutants. Environ. Sci.
Technol..

[17] Li L.-P., Liu Y.-H., Ren D., Wang J.-J. (2022). Characteristics
and Chlorine Reactivity of Biochar-Derived Dissolved Organic Matter:
Effects of Feedstock Type and Pyrolysis Temperature. Water Res..

[18] Lopez A. M., Avila C. C. E., VanderRoest J. P., Roth H. K., Fendorf S., Borch T. (2024). Molecular Insights
and Impacts of Wildfire-Induced Soil Chemical
Changes. Nat. Rev. Earth Environ..

[19] Plewa, M. J. ; Wagner, E. D. ; Muellner, M. G. ; Hsu, K. M. ; Richardson, S. D. Comparative Mammalian Cell Toxicity of N-DBPs and C-DBPs. In Abstracts of Papers of the American Chemical Society; American Chemical Society: 1155 16th St, NW, Washington, DC 20036 USA, 2007; Vol. 233, pp 372–372.

[20] Berg S. M., Wammer K. H., Remucal C. K. (2023). Dissolved
Organic Matter Photoreactivity
Is Determined by Its Optical Properties, Redox Activity, and Molecular
Composition. Environ. Sci. Technol..

[21] Önnby L., Salhi E., McKay G., Rosario-Ortiz F. L., von Gunten U. (2018). Ozone and Chlorine Reactions with Dissolved Organic
Matter - Assessment of Oxidant-Reactive Moieties by Optical Measurements
and the Electron Donating Capacities. Water
Res..

[22] Rougé V., von Gunten U., Allard S. (2020). Efficiency of Pre-Oxidation of Natural
Organic Matter for the Mitigation of Disinfection Byproducts: Electron
Donating Capacity and UV Absorbance as Surrogate Parameters. Water Res..

[23] Zheng X., Liu Y., Fu H., Qu X., Yan M., Zhang S., Zhu D. (2019). Comparing Electron Donating/Accepting
Capacities (EDC/EAC) between
Crop Residue-Derived Dissolved Black Carbon and Standard Humic Substances. Sci. Total Environ..

[24] Cawley K. M., Hohner A. K., Podgorski D. C., Cooper W. T., Korak J. A., Rosario-Ortiz F. L. (2017). Molecular
and Spectroscopic Characterization of Water
Extractable Organic Matter from Thermally Altered Soils Reveal Insight
into Disinfection Byproduct Precursors. Environ.
Sci. Technol..

[25] Xu W., Walpen N., Keiluweit M., Kleber M., Sander M. (2021). Redox Properties
of Pyrogenic Dissolved Organic Matter (pyDOM) from Biomass-Derived
Chars. Environ. Sci. Technol..

[26] Jaffé R., Ding Y., Niggemann J., Vähätalo A. V., Stubbins A., Spencer R. G. M., Campbell J., Dittmar T. (2013). Global Charcoal
Mobilization from Soils via Dissolution and Riverine Transport to
the Oceans. Science.

[27] McDonough L. K., O’Carroll D. M., Meredith K., Andersen M. S., Brugger C., Huang H., Rutlidge H., Behnke M. I., Spencer R. G. M., McKenna A., Marjo C. E., Oudone P., Baker A. (2020). Changes in
Groundwater Dissolved Organic Matter Character in a Coastal Sand Aquifer
Due to Rainfall Recharge. Water Res..

[28] Lee M.-H., Ok Y. S., Hur J. (2018). Dynamic Variations
in Dissolved Organic
Matter and the Precursors of Disinfection By-Products Leached from
Biochars: Leaching Experiments Simulating Intermittent Rain Events. Environ. Pollut..

[29] Zhang R., Deng Z., Li J., Zhang Y., Wei Z., Cao H. (2022). Effect of Leaching
Time on Phytotoxicity of Dissolved Organic Matter
Derived from Black Carbon Based on Spectroscopy. Environ. Pollut..

[30] Aeschbacher M., Graf C., Schwarzenbach R. P., Sander M. (2012). Antioxidant Properties
of Humic Substances. Environ. Sci. Technol..

[31] Ou Q., Xu Y., Wang X., van der Hoek J. P., Yu G., Liu G. (2024). Dissolved
Black Carbon Facilitates the Photodegradation of Microplastics via
Molecular Weight-Dependent Generation of Reactive Intermediates. Environ. Sci. Technol..

[32] Yang P., Wang Y., Tian X., Cui Y., Jiang T., Liu G., Liu Y., Guo Y., Hu L., Shi J., Zhang Q., Yin Y., Cai Y., Jiang G. (2025). Heating-Induced
Redox Property Dynamics of Peat Soil Dissolved Organic Matter in a
Simulated Peat Fire: Electron Exchange Capacity and Molecular Characteristics. Environ. Sci. Technol..

[33] Mitch W. A., Richardson S. D., Zhang X., Gonsior M. (2023). High-Molecular-Weight
by-Products of Chlorine Disinfection. Nat. Water.

[34] Dong H., Cuthbertson A. A., Plewa M. J., Weisbrod C. R., McKenna A. M., Richardson S. D. (2023). Unravelling High-Molecular-Weight DBP Toxicity Drivers
in Chlorinated and Chloraminated Drinking Water: Effect-Directed Analysis
of Molecular Weight Fractions. Environ. Sci.
Technol..

[35] Gao Q., Pan Y., Zhou Y., Peng J., Kong Q., Cheng Y., Fu Q.-L., Yang X. (2025). Molecular Composition Difference
of Electron Donating Moieties between Natural Organic Matter and Effluent
Organic Matter Probed by Chlorine Dioxide. Water
Res..

[36] Sun Y., Xu Z., He M., Alessi D. S., Tsang D. C. W. (2024). Unlocking the
Solution-Phase Molecular Transformation of Biochar during Intensive
Rainfall Events: Implications for the Long-Term Carbon Cycle under
Climate Change. Sci. Total Environ..

[37] Zhang X., Kang J., Chu W., Zhao S., Shen J., Chen Z. (2020). Spectral and Mass Spectrometric
Characteristics of Different Molecular
Weight Fractions of Dissolved Organic Matter. Sep. Purif. Technol..

[38] Stubbins A., Lapierre J.-F., Berggren M., Prairie Y. T., Dittmar T., del Giorgio P. A. (2014). What’s in an EEM? Molecular
Signatures Associated
with Dissolved Organic Fluorescence in Boreal Canada. Environ. Sci. Technol..

[39] Zhu S., Yang P., Yin Y., Zhang S., Lv J., Tian S., Jiang T., Wang D. (2024). Influences of Wildfire
on the Soil Dissolved Organic Matter Characteristics and Its Electron-Donating
Capacity. Water Res..

[40] Leyva D., Usman Tariq M., Jaffé R., Saeed F., Fernandez-Lima F. (2023). Description
of Dissolved Organic Matter Transformational Networks at the Molecular
Level. Environ. Sci. Technol..

[41] Chen B., Zhang T., Bond T., Gan Y. (2015). Development of Quantitative
Structure Activity Relationship (QSAR) Model for Disinfection Byproduct
(DBP) Research: A Review of Methods and Resources. J. Hazard. Mater..

[42] Klein O. I., Kulikova N. A., Filimonov I. S., Koroleva O. V., Konstantinov A. I. (2018). Long-Term
Kinetics Study and Quantitative Characterization of the Antioxidant
Capacities of Humic and Humic-like Substances. J. Soils Sediments.

[43] Yang P., Jiang T., Cong Z., Liu G., Guo Y., Liu Y., Shi J., Hu L., Yin Y., Cai Y., Jiang G. (2022). Loss and Increase of the Electron
Exchange Capacity of Natural Organic
Matter during Its Reduction and Reoxidation: The Role of Quinone and
Nonquinone Moieties. Environ. Sci. Technol..

[44] Knicker H., Hilscher A., González-Vila F. J., Almendros G. (2008). A New Conceptual
Model for the Structural Properties of Char Produced during Vegetation
Fires. Org. Geochem..

[45] Knicker H. (2010). “Black
Nitrogen” – an Important Fraction in Determining the
Recalcitrance of Charcoal. Org. Geochem..

[46] He M., Zhu X., Dutta S., Khanal S. K., Lee K. T., Masek O., Tsang D. C. W. (2022). Catalytic
Co-Hydrothermal Carbonization of Food Waste
Digestate and Yard Waste for Energy Application and Nutrient Recovery. Bioresour. Technol..

[47] Li C., Li J., Pan L., Zhu X., Xie S., Yu G., Wang Y., Pan X., Zhu G., Angelidaki I. (2020). Treatment
of Digestate Residues for Energy Recovery and Biochar Production:
From Lab to Pilot-Scale Verification. J. Clean.
Prod..

[48] Zhang Z., Cui X., Qu X., Fu H., Tao S., Zhu D. (2024). Revealing
Molecular Structures of Nitrogen-Containing Compounds in Dissolved
Black Carbon Using Ultrahigh-Resolution Mass Spectrometry Combined
with Thermodynamic Calculations. Environ. Sci.
Technol..

[49] United States Environmental Protection Agency. SW-846 Test Method 1312: Synthetic Precipitation Leaching Procedure. https://www.epa.gov/hw-sw846/sw-846-test-method-1312-synthetic-precipitation-leaching-procedure (accessed 2023–08–07).

[50] Khan E., Subramania-Pillai S. (2007). Interferences Contributed by Leaching from Filters
on Measurements of Collective Organic Constituents. Water Res..

[51] Aeschbacher M., Sander M., Schwarzenbach R. P. (2010). Novel Electrochemical
Approach to
Assess the Redox Properties of Humic Substances. Environ. Sci. Technol..

[52] Walpen N., Houska J., Salhi E., Sander M., von Gunten U. (2020). Quantification
of the Electron Donating Capacity and UV Absorbance of Dissolved Organic
Matter during Ozonation of Secondary Wastewater Effluent by an Assay
and an Automated Analyzer. Water Res..

[53] Weishaar J. L., Aiken G. R., Bergamaschi B. A., Fram M. S., Fujii R., Mopper K. (2003). Evaluation of Specific
Ultraviolet Absorbance as an
Indicator of the Chemical Composition and Reactivity of Dissolved
Organic Carbon. Environ. Sci. Technol..

[54] Murphy K.
R., Stedmon C. A., Graeber D., Bro R. (2013). Fluorescence Spectroscopy
and Multi-Way Techniques. PARAFAC. Anal. Methods.

[55] Qu X., Fu H., Mao J., Ran Y., Zhang D., Zhu D. (2016). Chemical and
Structural Properties of Dissolved Black Carbon Released from Biochars. Carbon.

[56] Almendros G., Knicker H., González-Vila F. J. (2003). Rearrangement
of
Carbon and Nitrogen Forms in Peat after Progressive Thermal Oxidation
as Determined by Solid-State ^13^C- and ^15^N-NMR
Spectroscopy. Org. Geochem..

[57] Koch B. P., Dittmar T. (2016). From Mass to Structure:
An Aromaticity Index for High-Resolution
Mass Data of Natural Organic Matter. Rapid Commun.
Mass Spectrom..

[58] Koch B. P., Dittmar T. (2006). From Mass to Structure: An Aromaticity Index for High-Resolution
Mass Data of Natural Organic Matter. Rapid Commun.
Mass Spectrom..

[59] He W., Chen M., Park J.-E., Hur J. (2016). Molecular Diversity
of Riverine Alkaline-Extractable Sediment Organic Matter and Its Linkages
with Spectral Indicators and Molecular Size Distributions. Water Res..

[60] Abdulla H. A. N., Sleighter R. L., Hatcher P. G. (2013). Two Dimensional
Correlation Analysis
of Fourier Transform Ion Cyclotron Resonance Mass Spectra of Dissolved
Organic Matter: A New Graphical Analysis of Trends. Anal. Chem..

[61] Li T., Ruan M., Cao Y., Feng W., Song F., Bai Y., Zhao X., Wu F. (2024). Molecular-Level Insights into the
Temperature-Dependent Formation Dynamics and Mechanism of Water-Soluble
Dissolved Organic Carbon Derived from Biomass Pyrolysis Smoke. Water Res..

[62] Noda I. (1989). Two-Dimensional
Infrared Spectroscopy. J. Am. Chem. Soc..

[63] Noda I. (1986). Two-Dimensional
Infrared (2D IR) Spectroscopy of Synthetic and Biopolymers. Bull. Am. Phys. Soc..

[64] Song F., Li T., Hur J., Shi Q., Wu F., He W., Shi D., He C., Zhou L., Ruan M., Cao Y. (2023). Molecular-Level
Insights into the Heterogeneous Variations and Dynamic Formation Mechanism
of Leached Dissolved Organic Matter during the Photoaging of Polystyrene
Microplastics. Water Res..

[65] Wu W., Wang K., Liu J., So P.-K., Leung T.-F., Wong M., Zhao D. (2025). A High-Throughput
Integrated Nontargeted
Metabolomics and Lipidomics Workflow Using Microelution Enhanced Matrix
Removal-Lipid for Comparative Analysis of Human Maternal and Umbilical
Cord Blood Metabolomes. Anal. Chem..

[66] Zhang X., Shen J., Huo X., Li J., Zhou Y., Kang J., Chen Z., Chu W., Zhao S., Bi L., Xu X., Wang B. (2021). Variations of Disinfection Byproduct
Precursors through Conventional Drinking Water Treatment Processes
and a Real-Time Monitoring Method. Chemosphere.

[67] Arrieta J. M., Mayol E., Hansman R. L., Herndl G. J., Dittmar T., Duarte C. M. (2015). Dilution Limits
Dissolved Organic Carbon Utilization
in the Deep Ocean. Science.

[68] Wang M., Liu J., Peng L., Tian S., Yang C., Xu G., Wang D., Jiang T. (2021). Estimation of the Biogeochemical
Reactivities of Dissolved Organic Matter from Modified Biochars Using
Color. Sci. Total Environ..

[69] Song J., Li M., Zou C., Cao T., Fan X., Jiang B., Yu Z., Jia W., Peng P. (2022). Molecular Characterization of Nitrogen-Containing
Compounds in Humic-like Substances Emitted from Biomass Burning and
Coal Combustion. Environ. Sci. Technol..

[70] Wagner S., Dittmar T., Jaffé R. (2015). Molecular Characterization of Dissolved
Black Nitrogen via Electrospray Ionization Fourier Transform Ion Cyclotron
Resonance Mass Spectrometry. Org. Geochem..

[71] Zhu S., Huang X., Yang X., Peng P., Li Z., Jin C. (2020). Enhanced Transformation
of Cr­(VI) by Heterocyclic-N within Nitrogen-Doped
Biochar: Impact of Surface Modulatory Persistent Free Radicals (PFRs). Environ. Sci. Technol..

[72] Wu S., Wang D., Liu C., Fang G., Sun T.-R., Cui P., Yan H., Wang Y., Zhou D. (2021). Pyridinic- and Pyrrolic
Nitrogen in Pyrogenic Carbon Improves Electron Shuttling during Microbial
Fe­(III) Reduction. ACS Earth Space Chem..

[73] Wu S., You F., Boughton B., Liu Y., Nguyen T. A. H., Wykes J., Southam G., Robertson L. M., Chan T.-S., Lu Y.-R., Lutz A., Yu D., Yi Q., Saha N., Huang L. (2021). Chemodiversity of Dissolved Organic Matter and Its Molecular Changes
Driven by Rhizosphere Activities in Fe Ore Tailings Undergoing Eco-Engineered
Pedogenesis. Environ. Sci. Technol..

[74] Wu L.-N., Tian Z.-Y., Wang D., Zheng Z.-H., Jin K.-R., Liu B.-Z., Xie C., Xu Q., Wang Z.-D. (2022). Dinitriles
and Nitriles Are Common Intermediates of Pyrrole Pyrolysis. Combust. Flame.

[75] Giorgi G., Ponticelli F. (2005). Structural
Characterization and Regiochemical Differentiation
of α -Cyanoethylindole Isomers in the Gas Phase. J. Am. Soc. Mass Spectrom..

[76] LeClair J. P., Collett J. L., Mazzoleni L. R. (2012). Fragmentation
Analysis of Water-Soluble
Atmospheric Organic Matter Using Ultrahigh-Resolution FT-ICR Mass
Spectrometry. Environ. Sci. Technol..

[77] dos
Santos J. V., Goranov A. I., Fregolente L. G., Bisinoti M. C., Sun Z., Schmidt-Rohr K., Hatcher P. G. (2025). Deciphering the Chemistry of Condensed Aromatic “Black”
Carbon and Nitrogen in Amazonian Anthrosols. Environ. Sci. Technol..

[78] MacLean P. D., Chapman E. E., Dobrowolski S. L., Thompson A., Barclay L. R. C. (2008). Pyrroles
As Antioxidants: Solvent Effects and the Nature of the Attacking Radical
on Antioxidant Activities and Mechanisms of Pyrroles, Dipyrrinones,
and Bile Pigments. J. Org. Chem..

[79] Fang N., Yu S., Prior R. L. (2002). LC/MS/MS
Characterization of Phenolic Constituents
in Dried Plums. J. Agric. Food Chem..

[80] Assary R.
S., Brushett F. R., Curtiss L. A. (2014). Reduction Potential Predictions of
Some Aromatic Nitrogen-Containing Molecules. RSC Adv..

[81] Wang H., Zhou H., Ma J., Nie J., Yan S., Song W. (2020). Triplet Photochemistry of Dissolved Black Carbon and Its Effects
on the Photochemical Formation of Reactive Oxygen Species. Environ. Sci. Technol..

[82] Richardson S. D., Thruston A. D., Rav-Acha C., Groisman L., Popilevsky I., Juraev O., Glezer V., McKague A. B., Plewa M. J., Wagner E. D. (2003). Tribromopyrrole,
Brominated Acids, and Other Disinfection
Byproducts Produced by Disinfection of Drinking Water Rich in Bromide. Environ. Sci. Technol..

[83] Yang M., Zhang X. (2014). Halopyrroles: A New
Group of Highly Toxic Disinfection Byproducts
Formed in Chlorinated Saline Wastewater. Environ.
Sci. Technol..

