# Supplementary material for: Molecular Drivers of Electron-Donating Capacity in Dissolved Black Carbon from Nitrogen-Rich Pyrogenic Carbon
Source: Environ Sci Technol. 2025 Dec 10;60(1):1129–40. doi: 10.1021/acs.est.5c09050 (PMC12810247; doi:10.1021/acs.est.5c09050)
Supplement: Supplementary file 1 [file es5c09050_si_001.pdf]

Supporting information

Molecular Drivers of Electron-Donating Capacity in Dissolved Black Carbon from Nitrogen-Rich Pyrogenic Carbon

Xiaoxiao Zhang [a], Weijian Xu [a], Wenjing Tian [b], Eakalak Khan [c], Daniel C.W. Tsang [a,*]

[a] Department of Civil and Environmental Engineering, The Hong Kong University of Science and Technology, Hong Kong 999077, China

[b] Department of Civil and Environmental Engineering, The Hong Kong Polytechnic University, Hung Hom, Kowloon, Hong Kong 999077, China

[c] Civil and Environmental Engineering and Construction Department, University of Nevada, Las Vegas, Nevada 89154-4015, United States

This *Supporting Information* file has 53 pages, including 25 figures and 11 tables.

*Corresponding author: Daniel C.W. Tsang

Email: cedan@ust.hk

Table of Contents

Note S1. Materials and Methods

Note S1.1 Ultraviolet, Fluorescence, Fourier Infrared, X-ray Photoelectron Spectroscopy, and Nuclear Magnetic Resonance Spectroscopy Analyses

Note S1.2 Procedures of Solid Phase Extraction and Mass Spectrometry Analysis

Note S1.3 Tandem Mass Spectrometry Analysis Procedures and Parameter Settings

Note S2. Results and Discussion

Note S2.1 Sequential Leaching Responses of DBC Molecules

Note S2.2 DBC Functional Group Identification

Note S2.3 Functional Groups Related to DBC Molecules

Note S2.4 Sequential Leaching Responses of EDC-Related DBC Molecules

Note S2.5. Identification of Nitrogen Species in DBC by XPS and ^{13}C NMR

Figure Captions

Figure S1. Spectral properties of the 3 EEM-PARAFAC components identified in DBC samples. C1: ex/em 235, 345, 365/444 nm, terrestrial humic-like and/or N-heterocyclic fluorophores;[1,2] C2: 225, 340, 350/380 nm, soluble microbial byproduct-like;[3] C3: 230, 310/380 nm, microbial humic-like component. [4]

Figure S2. Van Krevelen diagrams and molecular feature contributions (histogram) of (a) DBC molecules (circular crossing markers represent average values). Regions in the van Krevelen diagram were divided into: 1. aliphatics and peptides, 2. highly unsaturated structures with low-oxygen (HUSLO), 3. highly unsaturated structures with high-oxygen (HUSHO), 4. aromatic structures (AS), 5. condensed aromatic structures (CAS), 6. sugars; (b) number percentages of seven molecular classes; (c) NOSC vs. (DBE–O)/C plots for DBC samples; (d) number percentages of unsaturated reduced (URC), saturated reduced (SRC), unsaturated oxidized (UOC), and saturated oxidized (SOC) compounds.

Figure S3. Van Krevelen diagrams and number contributions (histograms) of (a) DBC450 and (b) DBC550 molecules unique to each leaching cycle. Regions in the van Krevelen diagram were divided into: 1. aliphatics and peptides, 2. HUSLO, 3. HUSHO, 4. AS, 5. CAS, 6. sugars; NOSC vs. (DBE–O)/C plots for (c) DBC350, (d) DBC450, and (e) DBC550 molecules unique to different leaching cycles; (f) NOSC vs. (DBE–O)/C plots for molecules shared across leaching cycles.

Figure S4. (a) Number percentages of CHO, CHON, CHOS, and CHONS compounds across DBC samples; (b) corresponding percentages for compounds unique to each leaching cycle or shared among different leaching cycles.

Figure S5. H/N and O/N ratio distributions of N-bearing molecules in (a) DBC450 and (b) DBC550 samples; (c) number percentages of N0–N3 compounds in DBC samples; (d) number percentages of N0–N3 compounds unique to each leaching cycle or shared across different leaching cycles.

Figure S6. (a) Average H/N vs. H/C and O/N vs. O/C ratios for DBC350, DBC450, and DBC550 samples across leaching rounds; (b) relative spectral abundance of N0−N3 formulas unique to DBC350-2d, DBC450-2d, and DBC550-2d based on Venn analysis among them.

Figure S7. Correlations of H/N vs. H/C for (a1)–(a5) DBC350-2d–DBC350-30d, (b1)–(b5) DBC450-2d–DBC450-30d, and (c1)–(c5) DBC550-2d–DBC550-30d.

Figure S8. Correlations of O/N vs. O/C for (a1)–(a5) DBC350-2d–DBC350-30d, (b1)–(b5) DBC450-2d–DBC450-30d, and (c1)–(c5) DBC550-2d–DBC550-30d.

Figure S9. 2D-FTICR-MS-COS maps of (a) DBC350, (b) DBC450, and (c) DBC550 molecules across different leaching cycles based on H/C and O/C, and/or H/N and O/N ratios.

Figure S10. 2D-FTICR-MS-COS maps of (a) DBC450 and (b) DBC550 molecules across different leaching cycles based on *m/z*.

Figure S11. (a) FTIR spectra of DBC samples; 2D-FTIR-COS synchronous and asynchronous maps of (b) DBC450 and (c) DBC550 samples.

Figure 12. (a) Distribution and *m/z* range percentages of positive heterocorrelations between normalized intensities of molecules and absorbances of major functional groups in DBC350 samples; (b) illustration of the collaborative dynamic leaching mechanisms of DBC350 molecules and functional groups.

Figure S13. (a) 2D-FT-ICR MS/FTIR-COS heterocorrelation distributions of DBC450; (b) distribution and *m/z* range percentages of positive heterocorrelations between normalized intensities of molecules and absorbances of major functional groups in DBC450 samples; (c) illustration of the collaborative dynamic leaching mechanisms of DBC450 molecules and functional groups.

Figure S14. (a) 2D-FT-ICR MS/FTIR-COS heterocorrelation distributions of DBC550; (b)

distribution and *m/z* range percentages of positive heterocorrelations between normalized intensities of molecules and absorbances of major functional groups in DBC550 samples; (c) illustration of the collaborative dynamic leaching mechanisms of DBC550 molecules and functional groups.

Figure S15. 2D-FTICR-MS-COS maps of EDC-related (a) DBC350, (b) DBC450, and (c) DBC550 molecules based on H/C and O/C ratios.

Figure S16. 2D-FTICR-MS-COS maps of EDC-related (a) DBC350, (b) DBC450, and (c) DBC550 molecules based on H/N and O/N ratios.

Figure S17. 2D-FTICR-MS-COS maps of EDC-related (a) DBC350, (b) DBC450, and (c) DBC550 molecules based on *m/z*.

Figure S18. Distribution and *m/z* range percentages of positive heterocorrelations between normalized intensities of EDC-related molecules and FTIR absorbances of major functional groups in DBC350 samples. The representative heterocorrelation distribution at 1477 cm^{-1}, characterized by high intensity and shown in Figure 4c, is highlighted with virtual frames.

Figure S19. Distribution and *m/z* range percentages of positive heterocorrelations between normalized intensities of EDC-related molecules and FTIR absorbances of major functional groups in DBC450 samples. The representative heterocorrelation distribution at 1481 cm^{-1}, characterized by high intensity and shown in Figure 4c, is highlighted with virtual frames.

Figure S20. Distribution and *m/z* range percentages of positive heterocorrelations between normalized intensities of EDC-related molecules and FTIR absorbances of major functional groups in DBC350 samples. The representative heterocorrelation distribution at 1489 cm^{-1}, characterized by high intensity and shown in Figure 4c, is highlighted with virtual frames.

Figure S21. Observed neutral losses during fragmentation of six representative nitrogen-bearing EDC-related molecules with high intensities in DBC350.

Figure S22. Observed neutral losses during fragmentation of six representative nitrogen-bearing EDC-related molecules with high intensities in DBC450.

Figure S23. Observed neutral losses during fragmentation of six representative EDC-related molecules with high intensities in DBC550.

Figure S24. (a) N1s and (b) C1s XPS spectra of the day-2 DBC leachates and DFWD.

Figure S25. ^{13}C NMR spectra of the day-2 leachate of DBC350, food waste digestate (FWD), and biochar fabricated at 350 °C (BC350).

Table Captions

Table S1. The DOC concentrations and optical parameters of DBC samples

Table S2. Spearman's *r* between EDC and optical parameters (***: $p < 0.001$, **: $p < 0.01$, and *: $p < 0.05$)

Table S3. Intensity-weighted molecular parameters of DBC samples

Table S4. Intensity-weighted molecular parameters of S-bearing and S-free molecules

Table S5. Intensity-weighted molecular parameters of N-bearing and N-free molecules

Table S6. Spearman's *r* between EDC and molecular parameters ($n = 15$; ***: $p < 0.001$, **: $p < 0.01$, and *: $p < 0.05$)

Table S7. The primary sequential leaching responses of DBC molecules based on the generalized 2D–COS maps

Table S8. Main functional groups identified in FTIR spectra of DBC samples

Table S9. The primary sequential leaching responses of DBC functional groups based on the generalized 2D–COS maps

Table S10. The primary sequential leaching responses of EDC-related DBC molecules based on the generalized 2D–COS maps

Table S11. Intensity-weighted molecular parameters of EDC-related molecules

References

Note S1. Materials and Methods

Note S1.1 Ultraviolet, Fluorescence, Fourier Infrared, X-ray Photoelectron Spectroscopy, and Nuclear Magnetic Resonance Analyses

Specific ultraviolet (UV) absorbance at 254 nm ($SUVA_{254}$, L· mg^{-1}· m^{-1}), an indicator of aromaticity, was calculated as:[5]

$$SUVA_{254} = \frac{A_{254}}{I_L \times c_{\text{DOC}}} \tag{S1}$$

where A_{254} is the absorbance at 254 nm (Abs), which was recorded using a UV–vis spectrophotometer with a 1-cm quartz cuvette. I_L (0.01 m) is the path length of the optical cell in meter. c_{DOC} (mg·L^{-1}) is the dissolved organic carbon (DOC) concentration. The DOC concentration (mg·L^{-1}) was measured by a total organic carbon (TOC) analyzer (Shimadzu Corporation).

Three-dimensional excitation–emission matrix (EEM) fluorescence data of dissolved black carbon (DBC) samples were acquired using an FS5 Spectrofluorometer. Excitation and emission ranges were set at 200–450 nm and 300–550 nm, respectively, with step intervals of 5 nm (excitation) and 1 nm (emission). To minimize inner filter effects, samples were diluted with deionized water to achieve the $UV_{254} < 0.03$ and DOC < 1 mg·L^{-1}.[6] Raman calibration and blank subtraction were performed to reduce noise and enhance data reliability.[6] The humification index (HIX) was calculated as the ratio of fluorescence intensity at emission wavelengths 435–480 nm to 300–345 nm under 254 nm excitation.[7] Parallel factor analysis (PARAFAC) was employed using 2–7 component models with non-negativity constraints, and a three-component model was validated via split-half analysis (Figure S1).[8]

Fourier-transform infrared (FTIR) spectra of DBC samples were recorded using a Thermo Nicolet Nexus spectrometer. For the FTIR measurements, the DBC solutions were freeze-dried, then mixed with KBr powder for pellet preparation. Background spectra were collected prior to sample measurements. Each spectrum was averaged over multiple scans to enhance the signal-to-noise ratio, followed by baseline correction and smoothing using OMNIC software. Reproducibility and reliability were confirmed by consistent two-dimensional FTIR correlation spectroscopy (2D-FTIR-COS) patterns observed across replicate DBC samples.[9]

The nitrogen content and chemical states of freeze-dried DBC samples and dissolved food waste digestate (DFWD) were characterized using X-ray photoelectron spectroscopy (XPS) with Al Kα radiation (12 kV, 6 mA) under a base pressure of 5×10^{-1} Pa. Survey scans were performed with a pass energy of 150 eV and a step size of 1 eV. High-resolution spectra were acquired with a pass energy of 50 eV and a step size of 0.1 eV. Binding energies were calibrated against the C1s peak at 284.8 eV. The N1s XPS spectra were deconvoluted using a Shirley background and a Gaussian–Lorentzian fitting model (XPSPEAK41).[10,11] Major components such as pyrrolic-N species were deduced based on the previous studies.[11–15]

To elucidate nitrogen-associated structural changes, the quantitative ^{13}C solid-state nuclear magnetic resonance (NMR) analysis was performed on freeze-dried DBC350-2d, bulk biochar produced at 350 °C (BC350), and the food waste digestate. Spectra were collected on a Bruker Ascend 600 MHz spectrometer using a high-power proton decoupling pulse sequence, with a magic-angle spinning rate of 8 kHz, an acquisition time of 0.01 s, a recycle delay of 2 s, and 3072 scans. Major carbon groups were qualitatively classified according to broad chemical shift regions: alkyl C (0–45 ppm), O/N-alkyl C (45–90 ppm), aromatic C (90–148 ppm), O-aromatic C (148–167 ppm), carboxyl/amide C (167–184 ppm), and carbonyl C (184–220 ppm).[16]

Note S1.2 Procedures of Solid Phase Extraction and Mass Spectrometry Analysis

In the solid-phase extraction (SPE) procedure, Bond Elut PPL cartridges (6 mL, 1 g, Agilent Technologies, Folsom, CA) were preconditioned with two volumes of methanol and two volumes of 0.01 M HCl, respectively. DBC samples containing 0.6 mg DOC were acidified to pH 2 using 0.5 M H_2SO_4 and loaded onto the cartridges at a flow rate of < 5 mL min^{-1}. After loading, cartridges were rinsed with 12 mL of 0.01 M HCl to remove residual salts and dried under a gentle nitrogen stream for 5 min. DBC was eluted with two volumes of methanol. Each eluate was concentrated to ~1 mL under nitrogen gas before storage at −20 °C for analysis.

DBC extracts were introduced into a 15 T Fourier transform ion cyclotron resonance-mass spectrometry (FTICR-MS) coupled with a negative-mode electrospray ionization (ESI) source at 120 $\mu L \cdot h^{-1}$ for collecting *m/z* 100–800 data. The capillary entrance voltage and ion accumulation time were 4.0 kV and 0.08 s, respectively. FTICR-MS instrument calibration was conducted using a 10 mM sodium formate solution, followed by internal standard calibration with a homologous series of natural organic matter, ensuring mass assignment errors remained below 1 ppm.[17] For cases where *m/z* corresponded to multiple potential formulas, isotope patterns, the homologue rule, and the minimum heteroatom rule were adopted to determine the correct molecular formula.[9] Molecular parameters were calculated using following equations: the modified aromatic index (AI_{mod}) using $(1+C-0.5\times O-S-0.5\times(N+H))/(C-0.5\times O-N-S)$, the normal oxidation state of carbon (NOSC) using $(4\times C+H-2\times O-3\times N-2\times S))/C + 4$, and the double-bond equivalent (DBE) using $1+0.5\times(2\times C-H+N)$.[18–20]. Intensity-weighted molecular parameters (M_w) was calculated as $M_w = (\sum_i I_i * M_i)/\sum_i I_i$, where I_i and M_i are the relative abundance and molecular parameter value of FTICR-MS peak *i*, respectively.[21]

Note S1.3 Tandem Mass Spectrometry Analysis Procedures and Parameter Settings

For the tandem mass spectrometry (MS/MS) fragmentation analysis, 5 μL of DBC extract was injected into a UHPLC system coupled to an Orbitrap IQ-X Tribrid mass spectrometer, operating at 0.3 $mL \cdot min^{-1}$. The instrument ran in negative electrospray ionization mode using a data-dependent MS2 method. Full MS1 scans were acquired over *m/z* 100–800 at 120,000 resolution. MS2 spectra were triggered with an automatic gain control target of 1×10^5 and acquired using stepped higher-energy collisional dissociation (HCD) energies of 20%, 40%, and 60% at a 7500 resolution. Ion source parameters included a spray voltage of 2.4 kV, sheath gas flow of 40, auxiliary gas flow of 10 at 300 °C, and sweep gas flow of 2. The system was calibrated using Pierce™ FlexMix Ion Calibration Solutions (Thermo Fisher Scientific) prior to analysis. Data processing and compound annotation were conducted with Compound Discoverer v3.3 (Thermo Fisher Scientific), referencing local and online databases including mzCloud and ChemSpider.[22] Notably, only 23 molecular structures were identified through database searches, none of which showed a significant correlation with electron-donating capacity (EDC). Therefore, for the EDC-related molecules, we inferred their fragmentation patterns by considering commonly observed neutral losses (i.e., CO_2, H_2O) within a 5 ppm mass accuracy window.

Note S2.1 Sequential Leaching Responses of DBC Molecules

For DBC450, the leaching sequence followed: H/C: 2.00−2.20 → 1.15−1.35 → 0.45−0.95, and O/C: 0.55−0.70 → 0.45−0.55 (Figure S9 and Table S7), indicating that saturated and highly oxidized molecules leached first. Low-oxygen species (O/C < 0.50) exhibited complex signals, with no valid data extracted. Nitrogen-bearing DBC450 molecules displayed an alternating leaching pattern between saturated and unsaturated, as well as low-oxygen and high-oxygen structures (e.g., H/N: 23.0−39.0 → 2.5−7.5 → 39.0−43.0; O/N: 4.5−5.5 → 8.5−14.5 → 1.0−2.5). The divergence in leaching patterns between H/C and H/N corresponded with the low explanatory degree of H/N for H/C (12.1%–20.4%; $R^2 = 0.121–0.204$; Figure S7b).

Further analysis of the molecular leaching patterns revealed that DBC450 exhibited dominant positive synchronous signals in the *m/z* range 460−500/500−625, with negative asynchronous signals in the same ranges, suggesting a leaching sequence of *m/z* 500−625 → 460−500. Alternating signals in the *m/z* 375–450 range indicated a reduced size dependence for medium-weight molecules. In contrast, DBC550 showed no distinct synchronous or asynchronous signals at specific *m/z* intervals, indicating more complex hydrophilic and hydrophobic properties.

Note S2.2 DBC Functional Group Identification

Prominent functional groups correlated to different FTIR bands (Figures S11a) were identified based on previous studies.[23–25] A broad band at 3000–3600 cm^{-1} indicated O–H stretching (carboxylic/alcoholic/phenolic groups) and N–H vibrations (amines/heterocycles).[25] The peak near 2932 cm^{-1} was characteristic of aliphatic structures.[25] Signals around 1641 cm^{-1}, corresponded to aromatic C=C, carboxyl C–O, and amide C=O groups.[25] Peaks around 1482 cm^{-1} were attributed to aromatic C=C, while peaks around 1426 cm^{-1} can be attributed to lignin C=C, pyrrolic/ aliphatic C−H, and phenolic/ carboxylic C−O groups.[23,26–28] In the 1200–1000 cm^{-1} region, C–O stretching from polysaccharides, alcohols, ethers, and sulfur-containing compounds (C=S, S=O) were observed, with positions influenced by molecular surroundings.[23,29] For example, when C–O bond was directly attached to a benzene ring, as in aromatic ethers or phenols, the peaks shifted to lower wavenumbers (~1050 cm^{-1}).[30–32] Peaks around 866 cm^{-1} and 750 cm^{-1} were attributed to single aromatic hydrogen with 3–4 ring condensation/substitutions and aromatic ring with four adjacent hydrogens, respectively.[24,33,34] Peaks around 650 cm^{-1} indicated C−O and C−S stretching, associated with carbohydrates/alcohols and mercaptan/sulfur-rich compounds, respectively.[23,30,35]

After the first leaching cycle, DBC550 showed diminished FTIR peaks at ~3100–3600 cm^{-1} and 1647 cm^{-1}, indicating a lower retention of possible hydroxyl, amine, carboxyl, and amide groups.[23–25] In contrast, these functional groups remained pronounced in DBC350 and DBC450. These differences likely reflected variations in molecular aromaticity. In DBC550, these oxygen- and nitrogen-containing groups were more likely associated with aliphatic structures, whereas in DBC350 and DBC450, they were predominantly linked to aromatic frameworks (Table S3). Aromatic molecules, due to their hydrophobic nature, likely interact more strongly with the biochar matrix, leading to slower release, whereas aliphatic, oxygen-rich species, being more hydrophilic, were more water-soluble and leached in the early stages. However, individual FTIR peaks (e.g., 1647 cm^{-1}) may arise from multiple overlapping functional groups. Thus, integrating FTIR with complementary techniques such as FTICR-MS and 2D-COS is essential for more precise functional group identification.

Note S2.3 Functional Groups Related to DBC Molecules

Higher positive heterocorrelations between FTIR absorbances and normalized FTICR-MS peak intensities indicated that specific functional groups played a more significant role in shaping DBC molecular structures.[36] Notably, a single molecule can correlate strongly with multiple functional groups. For instance, in DBC350, $C_{17}H_{26}O_4$ (*m/z* 293.176) exhibited strong associations with 868, 1103, 1138, 1427, 1489, 1643, 3379 cm^{-1} (Figure S12), indicating the potential presence of diverse functional groups. In DBC450, $C_9H_{12}O_3S$ (m/z 199.044) and $C_{28}H_{24}N_2O_3$ (m/z 435.171) correlated strongly with secondary alcohol/ether C–O (1095 cm^{-1}), aliphatic C–OH (1146 cm^{-1}), and S-containing functional groups (650 cm^{-1}). In contrast, $C_{26}H_{37}N_3O_6$ (*m/z* 486.286) showed strong correlations with (hetero)aromatic C=C (1481 cm^{-1}), highlighting distinct functional group distributions. In DBC550, molecules such as $C_{17}H_{26}O_3$ (*m/z* 277.181) and $C_{36}H_{32}O_8$ (*m/z* 597.195) were strongly associated with oxygen-rich aliphatic groups (656, 1103, 1142, 1196 cm^{-1}). In contrast, $C_{26}H_{37}N_3O_6$ (*m/z* 486.286), $C_{19}H_{26}O_4$ (*m/z* 317.187), and $C_{12}H_{24}N_2O_5$ (*m/z* 311.169) showed strong correlations with both 1427 and 1466 cm^{-1}, suggesting (hetero)aromatic characteristics. These structural heterogeneity of DBC molecules and their diverse functional group associations likely played a major role in their sequential leaching behavior and evolving EDC profiles.

Regarding functional group molecular size distributions, in DBC450, FTIR peaks (652, 1041, 1095, 1146, 1196, 1427, and 1481 cm^{-1}) most corresponded to molecules spanning 150–800 *m/z*. Similar to DBC350, oxygen- and sulfur-rich functional groups (1095, 1146, 1196 cm^{-1}) were more linked to molecules with higher *m/z* 450–625 (42.3%–45.8%), while unsaturated aromatic or heterocyclic compounds (1041, 1427 and 1481 cm^{-1}) dominated medium *m/z* 275–450 (72.5%–74.6%). In DBC550, FTIR peaks (656, 1103, 1142, 1196, 1427, 1466 and 3317 cm^{-1}) primarily correlated with molecules in the *m/z* 275–450 range (45.6%–63.5%). More macromolecules (*m/z* > 450, 28.3%–33.9%) were linked to oxygen- and sulfur-rich functional groups (656 and 1050–1200 cm^{-1}) than aromatic structures (1427, 1466 and 3317 cm^{-1}, 7.9%–12.2%).

Note S2.4 Sequential Leaching Responses of EDC-Related DBC Molecules

The generalized synchronous maps showed only positive regions, indicating consistent increases over leaching, which can be attributed to the high consistency between Spearman ranking and 2D-COS analysis.[33,36,37] Asynchronous signals highlighted varied signals within even the same molecular classes, reflecting sequential leaching differences affecting by different structures related to hydrophilicity and hydrophobicity. Regardless of the pyrogenic temperature, compounds with high H/C leached faster and highly unsaturated EDC-related compounds (low H/C and H/N) contributed more at later sequences. The late-stage release of highly unsaturated EDC-DBC350/DBC450 aligned with bulk DBC leaching patterns (Figures 3 and S9, Table S7), while delayed leaching of unsaturated DBC550 molecules exposed previously unresolved dynamics in the overall 2D-COS maps (Figure S10, Table S7).

To examine the leaching sequence of molecular weights, sequential leaching dynamics of EDC-related DBC molecules were analyzed using 2D-FTICR MS-COS mapping of *m/z* (Figure S17). Synchronous maps displayed exclusively positive signals, whereas asynchronous maps revealed distinct leaching sequences. In the asynchronous map of EDC-related DBC350 molecules, negative cross-peaks at $v1/v2$ of *m/z* 350–450/310–350 range and positive cross-peaks in the *m/z* 280–350/250–280 range suggested sequential leaching orders of *m/z* 310−350 $\rightarrow$ 350−450 and *m/z* 280−350 $\rightarrow$ 250−280, indicating the prior release of medium-molecular-weight EDC-related molecules (*m/z* 280−350). In the asynchronous map of EDC-related DBC450 molecules, no distinct positive or negative signal ranges were observed, suggesting complex molecular weight dependencies. For EDC-related DBC550 molecules, negative asynchronous correlations in *m/z* 200–370/370–380 implied that larger molecules (*m/z* 370–380) leached earlier than smaller counterparts (*m/z* 200–370). The leaching sequence of molecular weights for EDC-related molecules deviated from the overall trends, indicating the influence of additional, more complex factors on *m/z* leaching dynamics.

Note S2.5 Identification of Nitrogen Species in DBC by XPS and ^{13}C NMR

The DFWD exhibited a dominant amide-N peak in the N1s spectrum at 399.9 eV (amide-N, 45%),[38] along with peaks at 398.8 eV (pyridinic-N, 29%) and 400.8 eV (graphitic-N, 26%) (Figure S24).[10,39] The dominance of amide-N was further confirmed by strong C1s peaks associated with amide C=O (288.6 eV) and C–N/C–O bonds (286.0 eV), consistent with its protein-rich origin.[9]

Pyrolysis substantially altered nitrogen speciation. In the N1s XPS spectra of DBC350–DBC550, the amide-N peak at ~399.9 eV shifted toward higher binding energy near ~400.3 eV (pyrrolic-N, 39%–64%),[10,39] indicating amide bond cleavage and the formation of heteroaromatic nitrogen structures.[11,12,14,16] This shift was accompanied by the loss of the C1s amide carbonyl peak at ~288.6 eV, reduction in C–N/C–O peak at ~ 286.0 eV,[40,41] and an enhancement of C–C/C=C peak (284.8 eV), consistent with peptide degradation and aromatic condensation. Meanwhile, a new peak near 289.0 eV appeared, characteristic of carboxyl/ester C=O groups,[42] reflecting partial oxidation of aromatic edges. Minor signals of amine/pyridone/pyridinic-N (399.2–399.4 eV), graphitic-N (401.1–401.2 eV), and N-oxides (403.1 eV) remained detectable.[15,38,43]

Solid-state ^{13}C NMR corroborated these structural changes (Figure S25). Marked decreases in alkyl C (0–45 ppm) and COO/NC=O (167–184 ppm) in DBC350 and BC350 indicate extensive decomposition of peptide-derived functionalities during carbonization, consistent with thermal release of CO_2, CO, NH_3, and HCNO below 350 °C.[9] Prior studies also reported that that nitrogen in pyrogenic carbon was predominantly incorporated into heteroaromatic forms with only trace residual amide content.[11,14–16,44]

Although amide-N was likely largely removed, a minor contribution cannot be entirely excluded due to overlapping N1s binding energies of amide- and pyrrolic-N (~400 eV), along with residual COO/NC=O resonances in the ^{13}C NMR (167–184 ppm; 45–90 ppm). Any remaining amide groups, if present, may still contribute to electron-donating characteristics of DBC.[45]

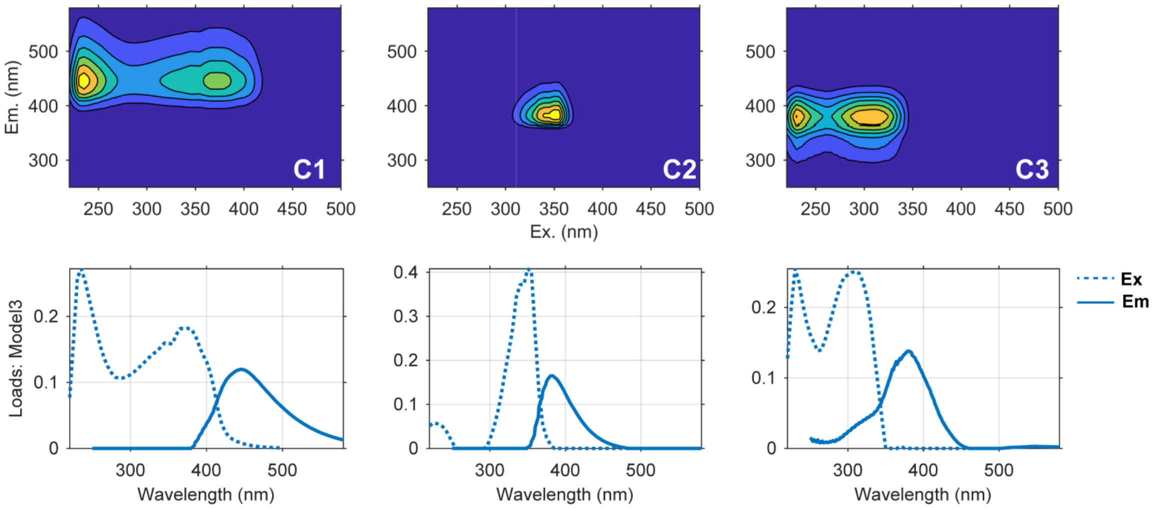

Figure S1. Spectral properties of the 3 EEM-PARAFAC components identified in DBC samples. C1: ex/em 235, 345, 365/444 nm, terrestrial humic-like and/or N-heterocyclic fluorophores;[1,2] C2: 225, 340, 350/380 nm, soluble microbial byproduct-like;[3] C3: 230, 310/380 nm, microbial humic-like component.[4]

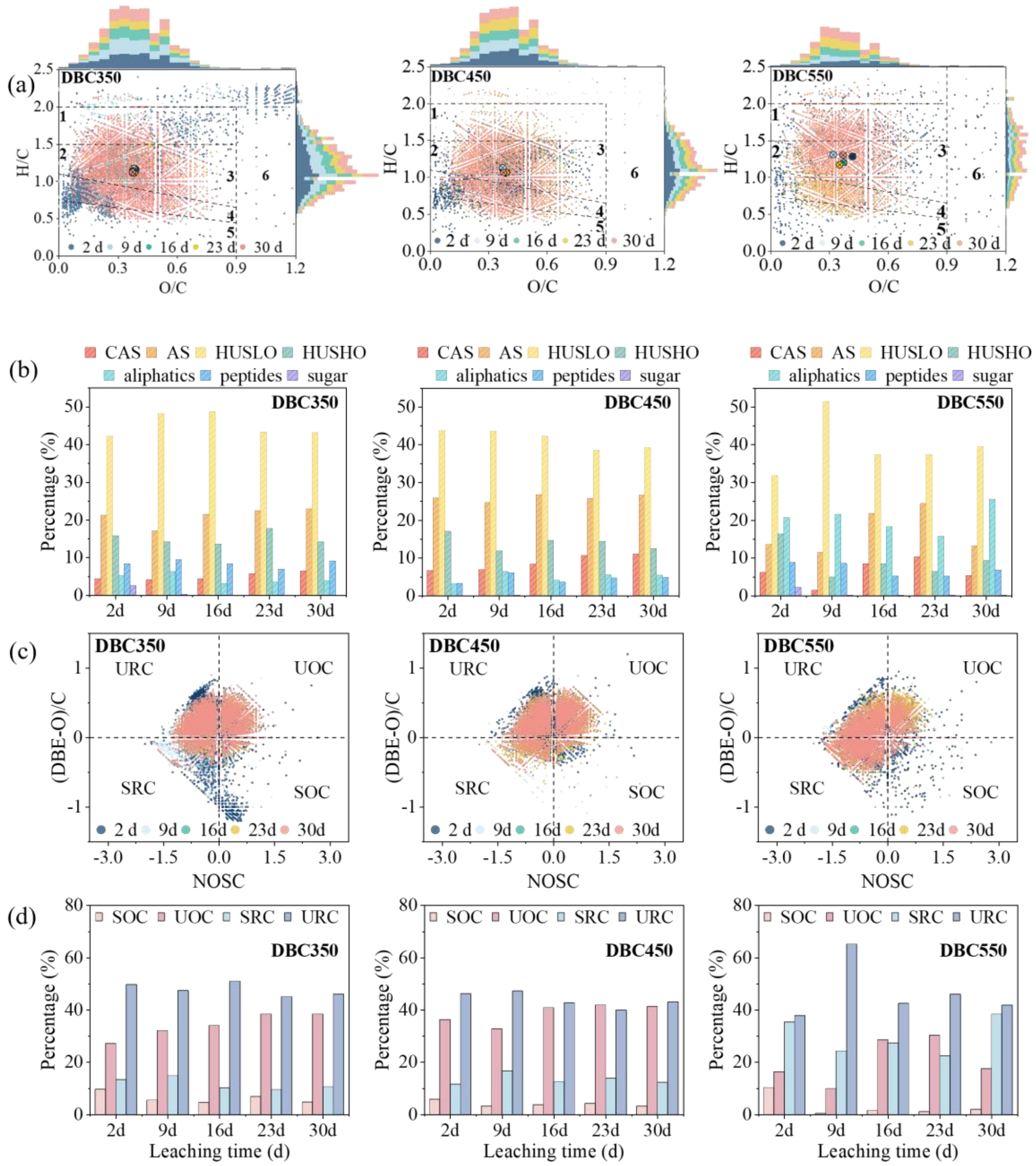

Figure S2. Van Krevelen diagrams and molecular feature contributions (histogram) of (a) DBC molecules (circular crossing markers represent average values). Regions in the van Krevelen diagram were divided into: 1. aliphatics and peptides, 2. highly unsaturated structures with low-oxygen (HUSLO), 3. highly unsaturated structures with high-oxygen (HUSHO), 4. aromatic structures (AS), 5. condensed aromatic structures (CAS), 6. sugars; (b) number percentages of seven molecular classes; (c) NOSC vs. (DBE–O)/C plots for DBC samples; (d) number percentages of unsaturated reduced (URC), saturated reduced (SRC), unsaturated oxidized (UOC), and saturated oxidized (SOC) compounds.

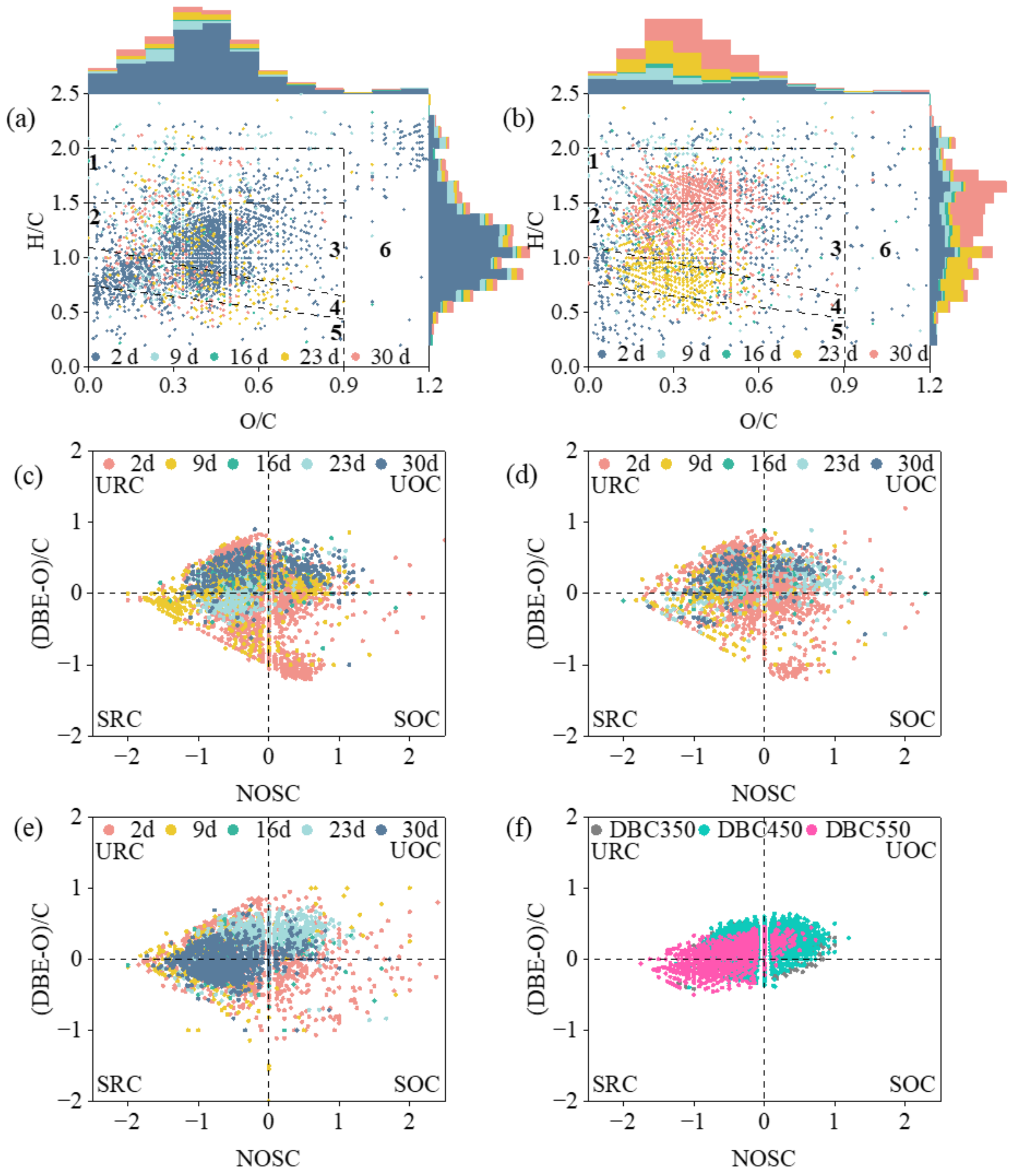

Figure S3. Van Krevelen diagrams and number contributions (histograms) of (a) DBC450 and (b) DBC550 molecules unique to each leaching cycle. Regions in the van Krevelen diagram were divided into: 1. aliphatics and peptides, 2. HUSLO, 3. HUSHO, 4. AS, 5. CAS, 6. sugars; NOSC vs. (DBE–O)/C plots for (c) DBC350, (d) DBC450, and (e) DBC550 molecules unique to different leaching cycles; (f) NOSC vs. (DBE–O)/C plots for molecules shared across leaching cycles.

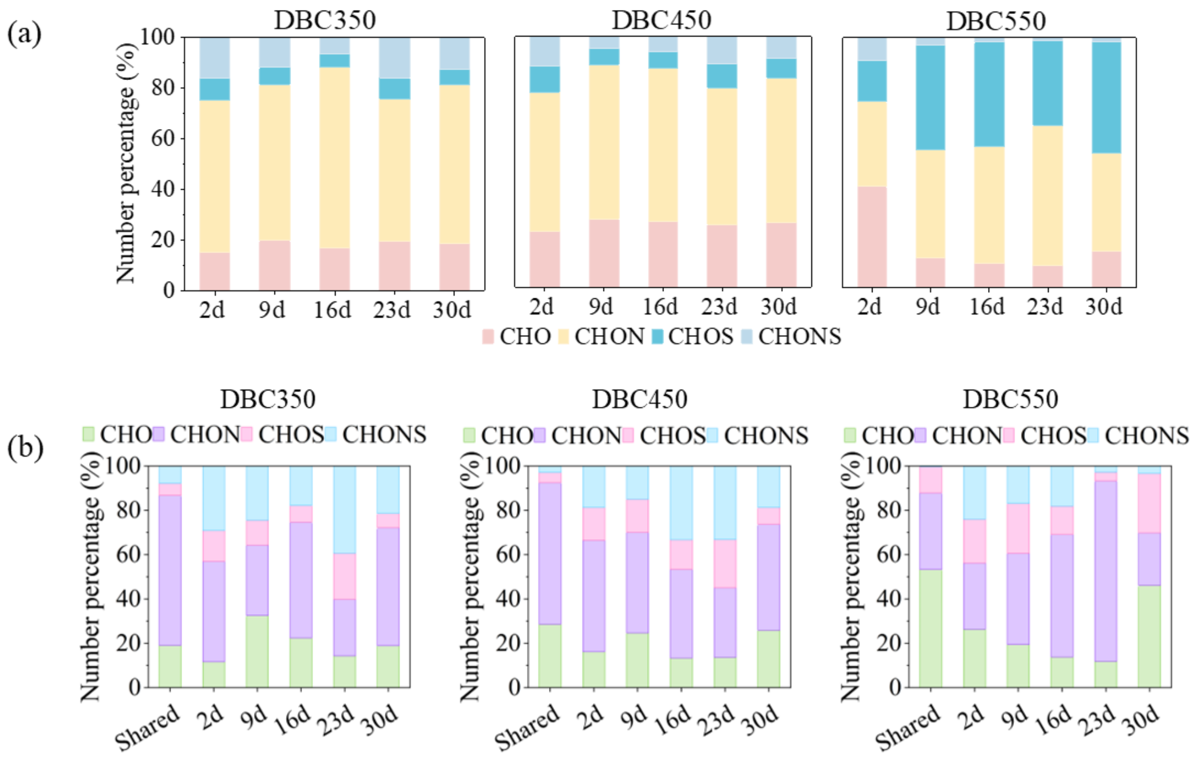

Figure S4. (a) Number percentages of CHO, CHON, CHOS, and CHONS compounds across DBC samples; (b) corresponding percentages for compounds unique to each leaching cycle or shared among different leaching cycles.

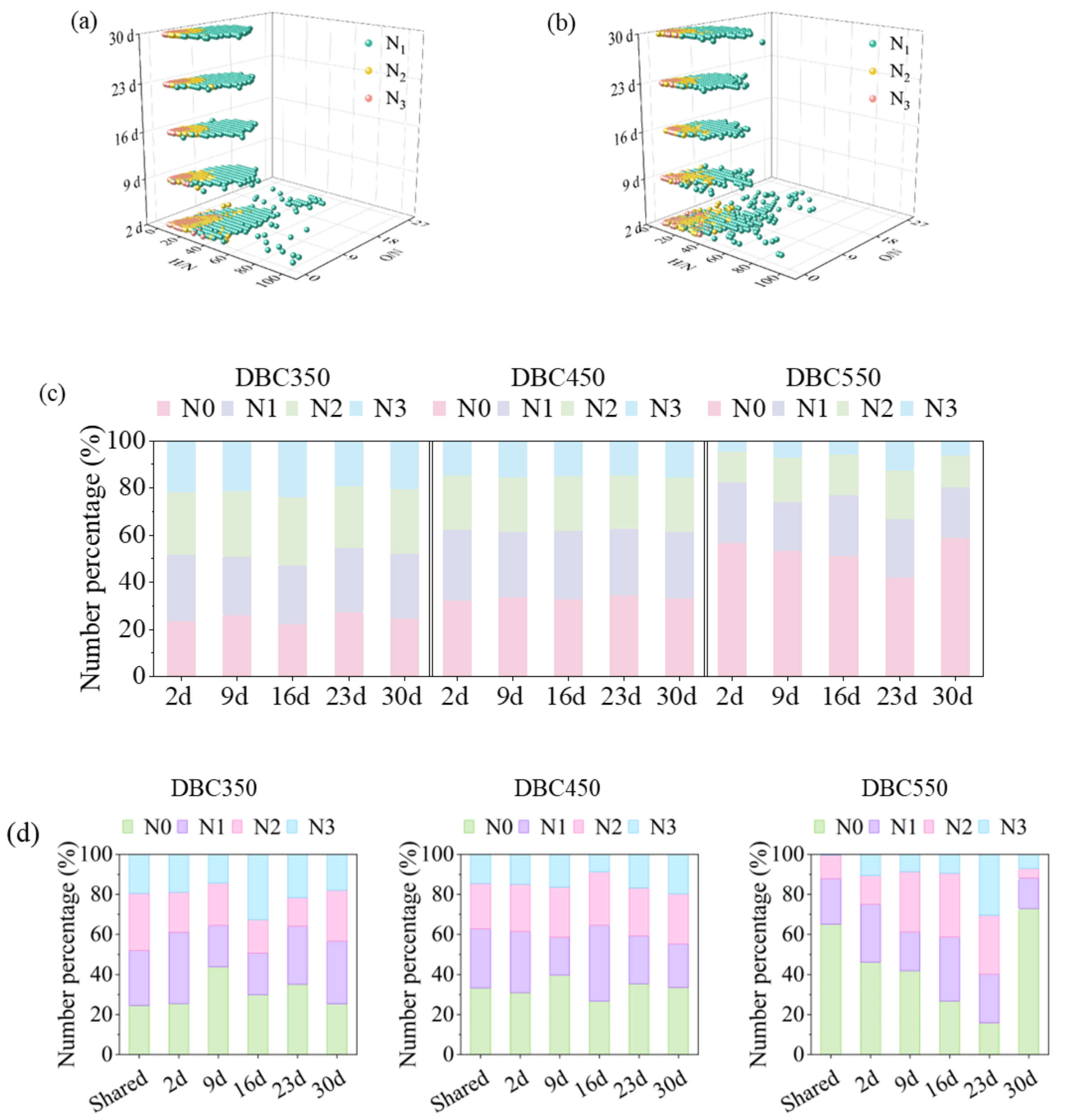

Figure S5. H/N and O/N ratio distributions of N-bearing molecules in (a) DBC450 and (b) DBC550 samples; (c) number percentages of N0–N3 compounds in DBC samples; (d) number percentages of N0–N3 compounds unique to each leaching cycle or shared across different leaching cycles.

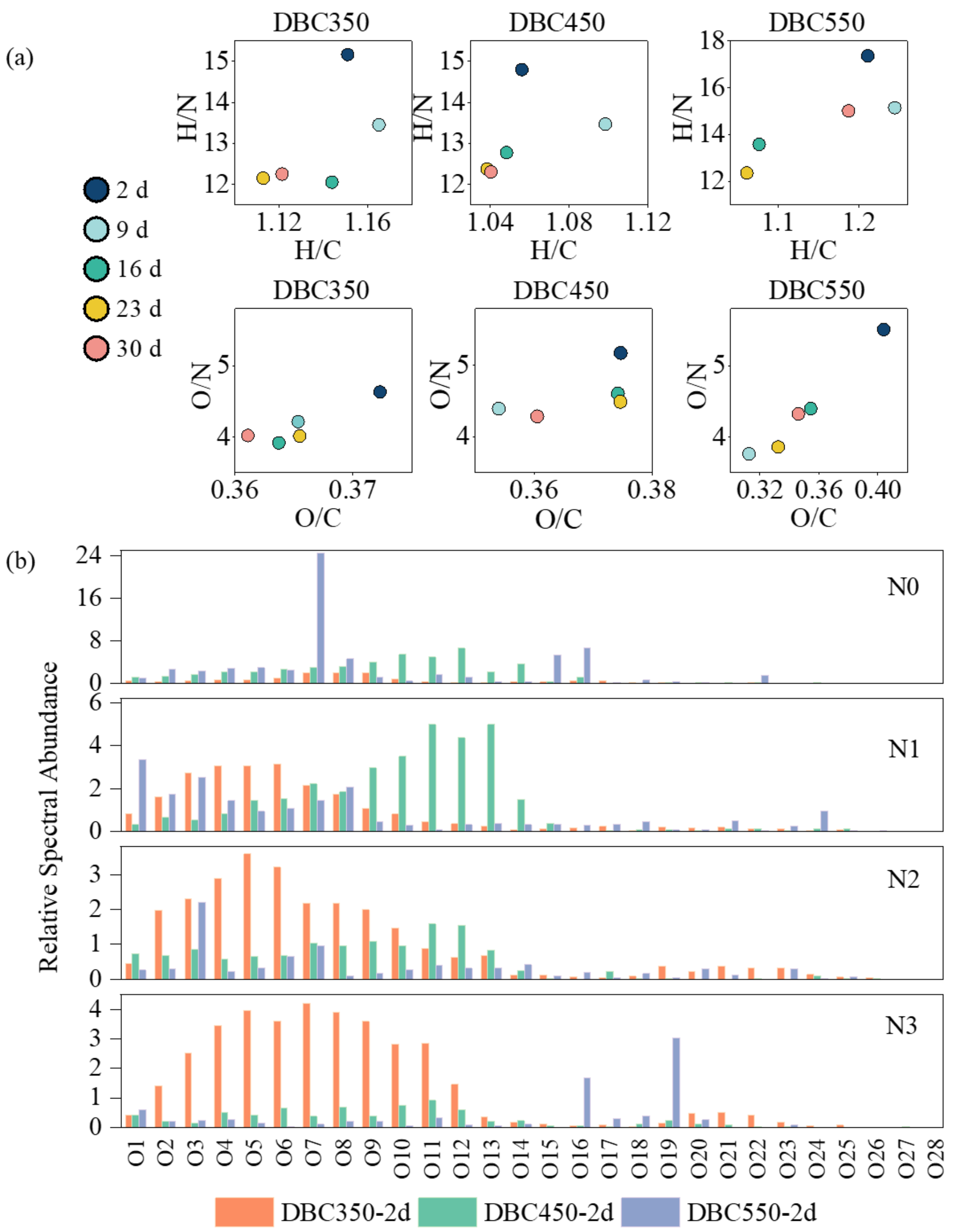

Figure S6. (a) Average H/N vs. H/C and O/N vs. O/C ratios for DBC350, DBC450, and DBC550 samples across leaching rounds; (b) relative spectral abundance of N0–N3 formulas unique to DBC350-2d, DBC450-2d, and DBC550-2d based on Venn analysis among them.

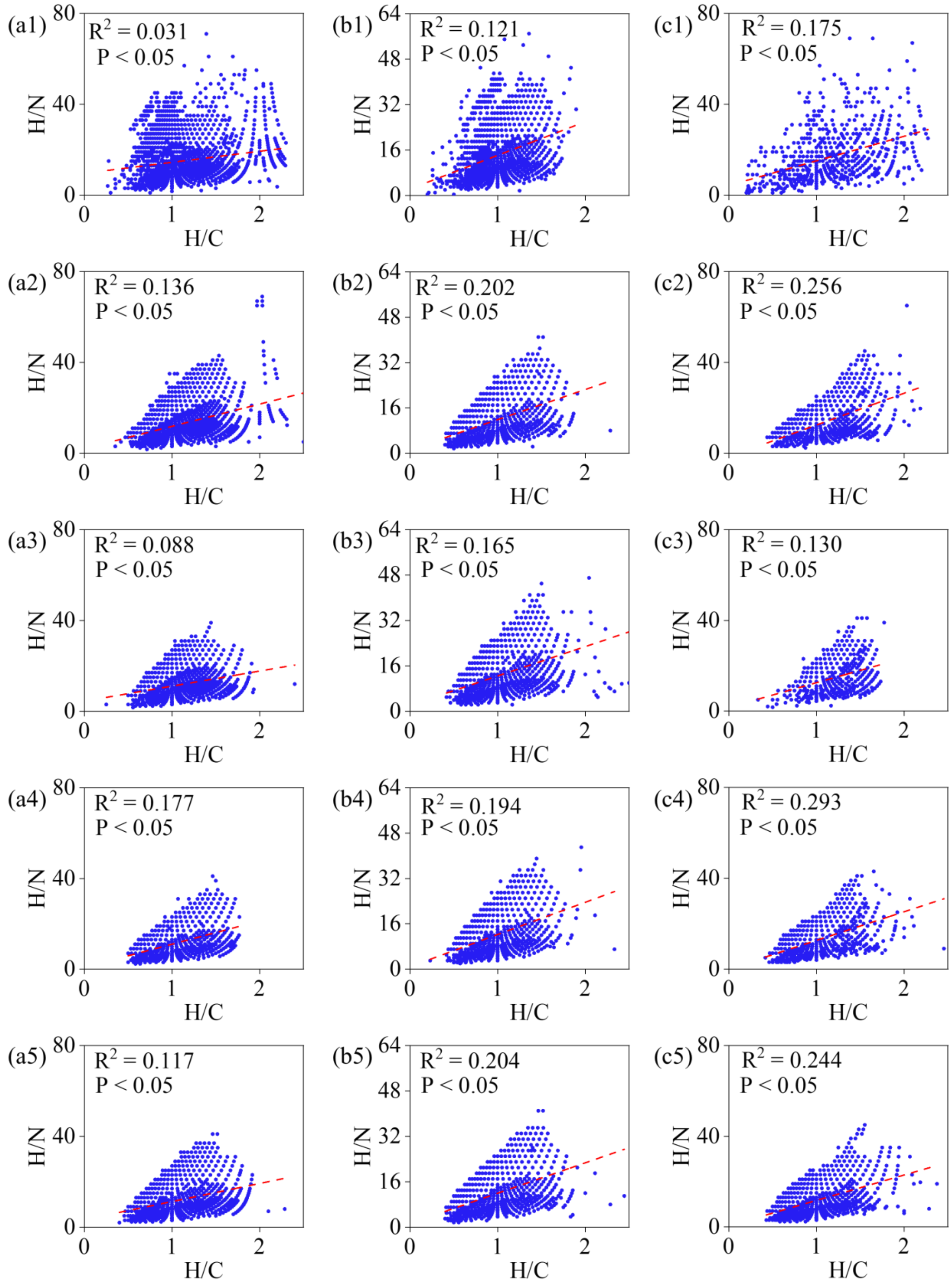

Figure S7. Correlations of H/N vs. H/C for (a1)–(a5) DBC350-2d–DBC350-30d, (b1)–(b5) DBC450-2d–DBC450-30d, and (c1)–(c5) DBC550-2d–DBC550-30d.

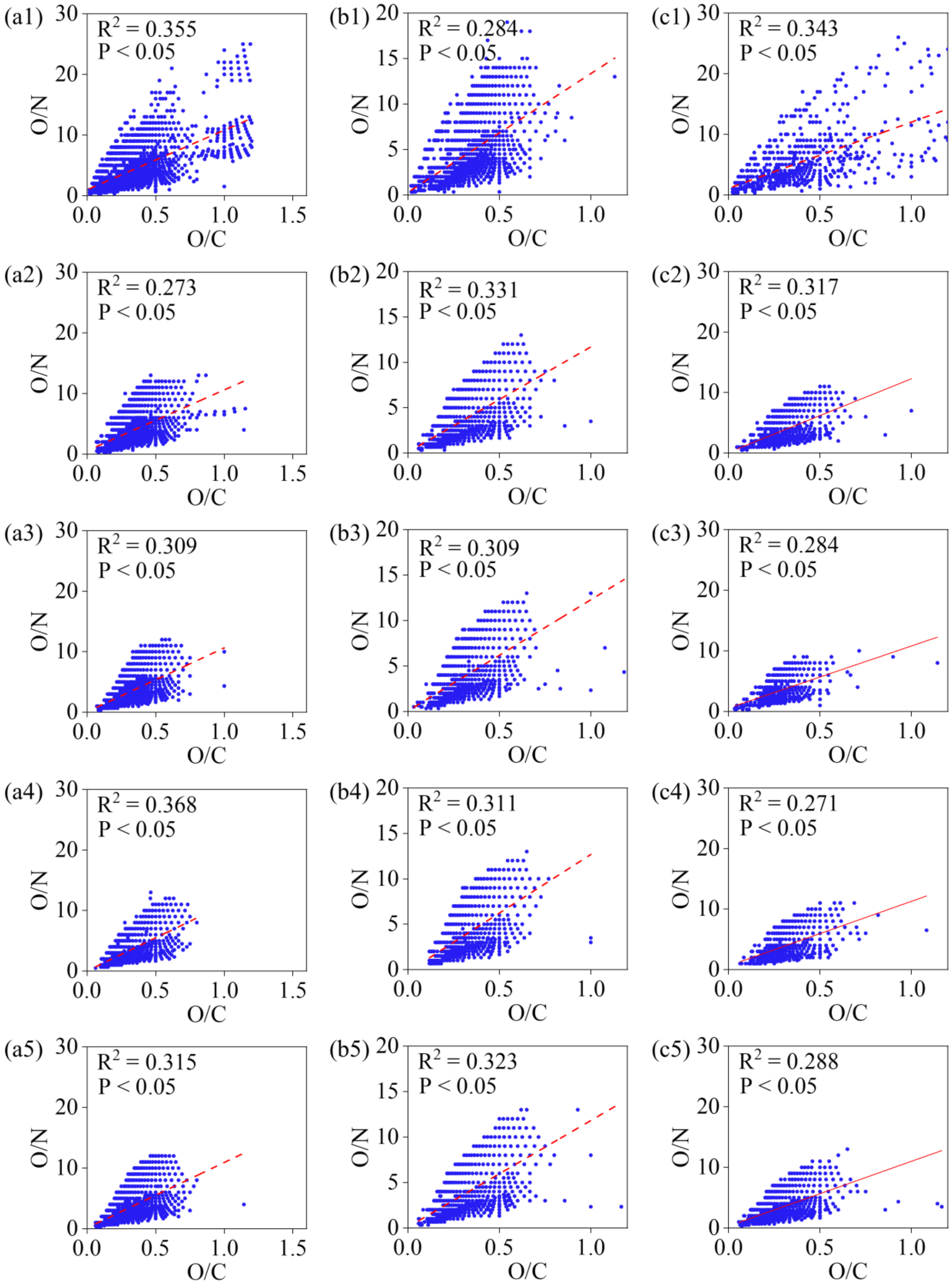

Figure S8. Correlations of O/N vs. O/C for (a1)–(a5) DBC350-2d–DBC350-30d, (b1)–(b5) DBC450-2d–DBC450-30d, and (c1)–(c5) DBC550-2d–DBC550-30d.

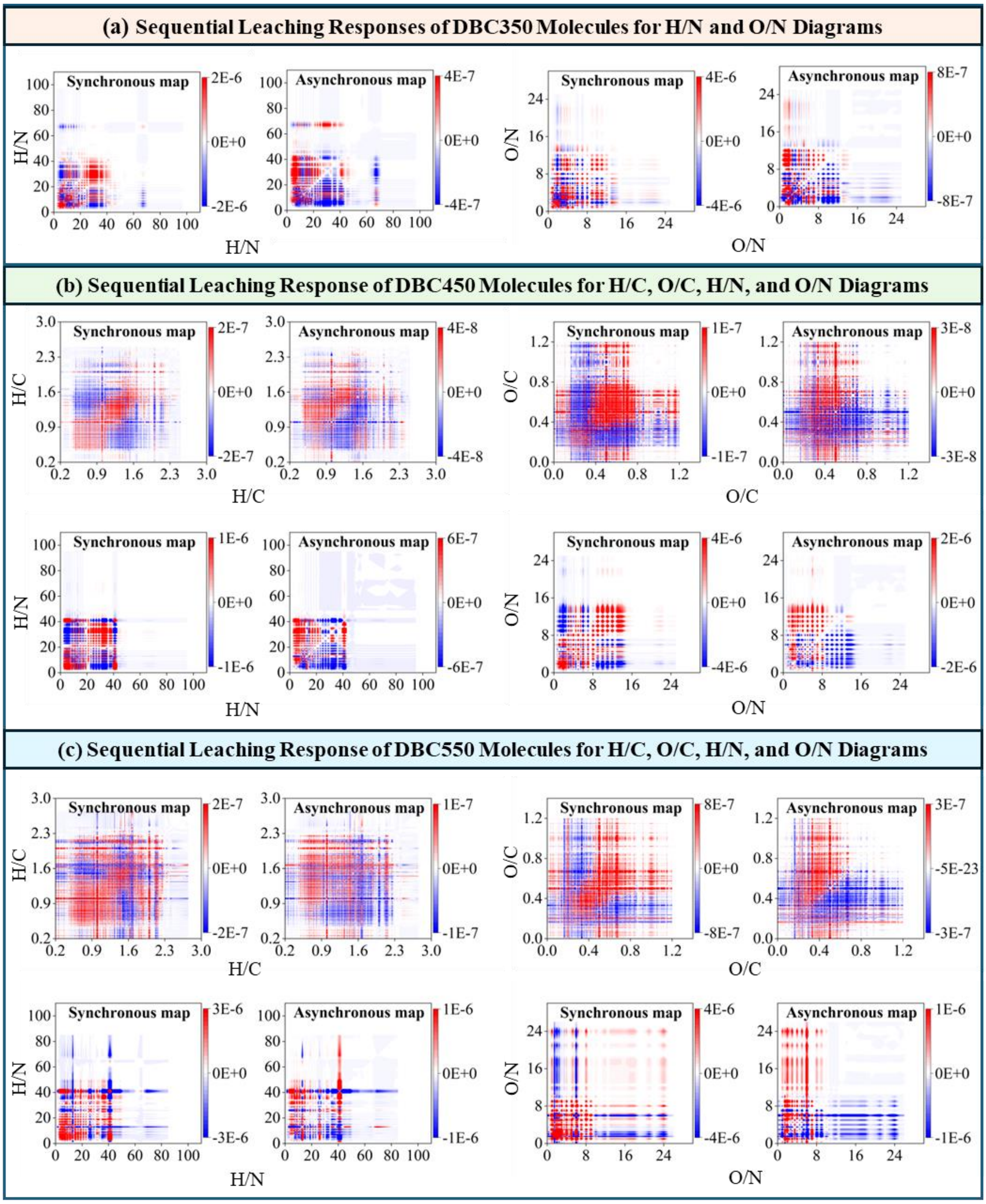

Figure S9. 2D-FTICR-MS-COS maps of (a) DBC350, (b) DBC450, and (c) DBC550 molecules across different leaching cycles based on H/C and O/C, and/or H/N and O/N ratios.

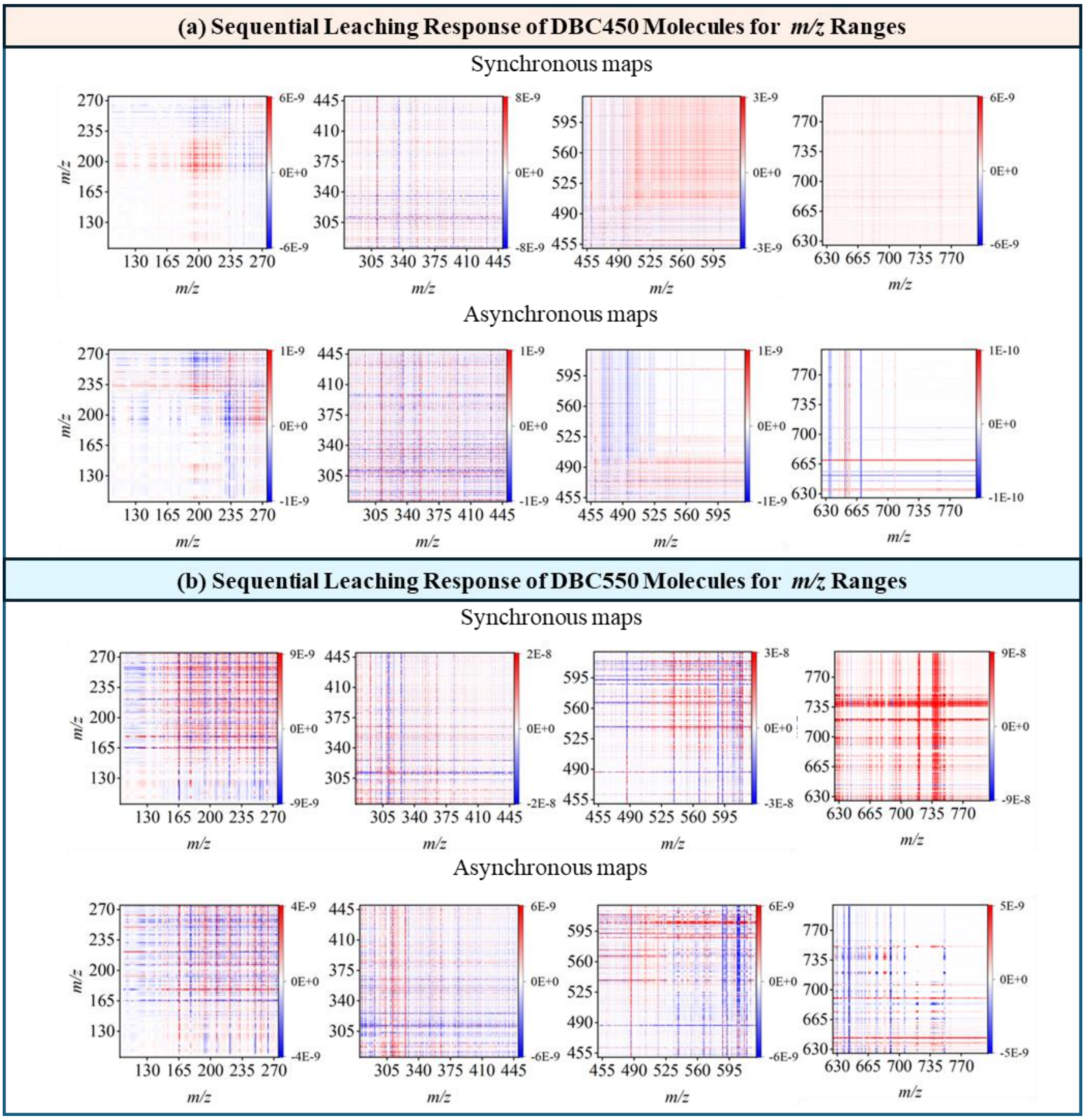

Figure S10. 2D-FTICR-MS-COS maps of (a) DBC450 and (b) DBC550 molecules across different leaching cycles based on *m/z*.

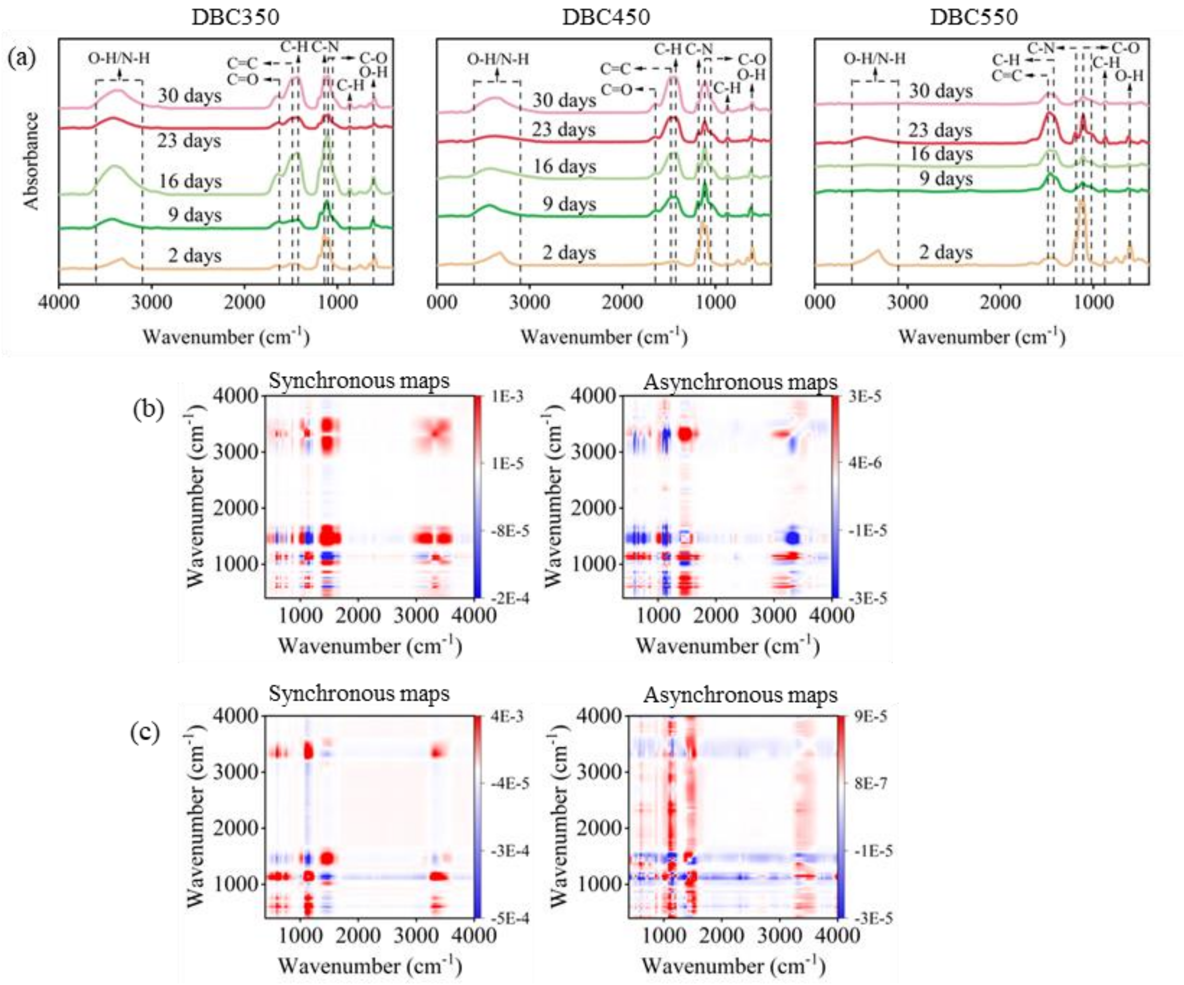

378

379 **Figure S11.** (a) FTIR spectra of DBC samples; 2D-FTIR-COS synchronous and asynchronous

380 maps of (b) DBC450 and (c) DBC550 samples.

381

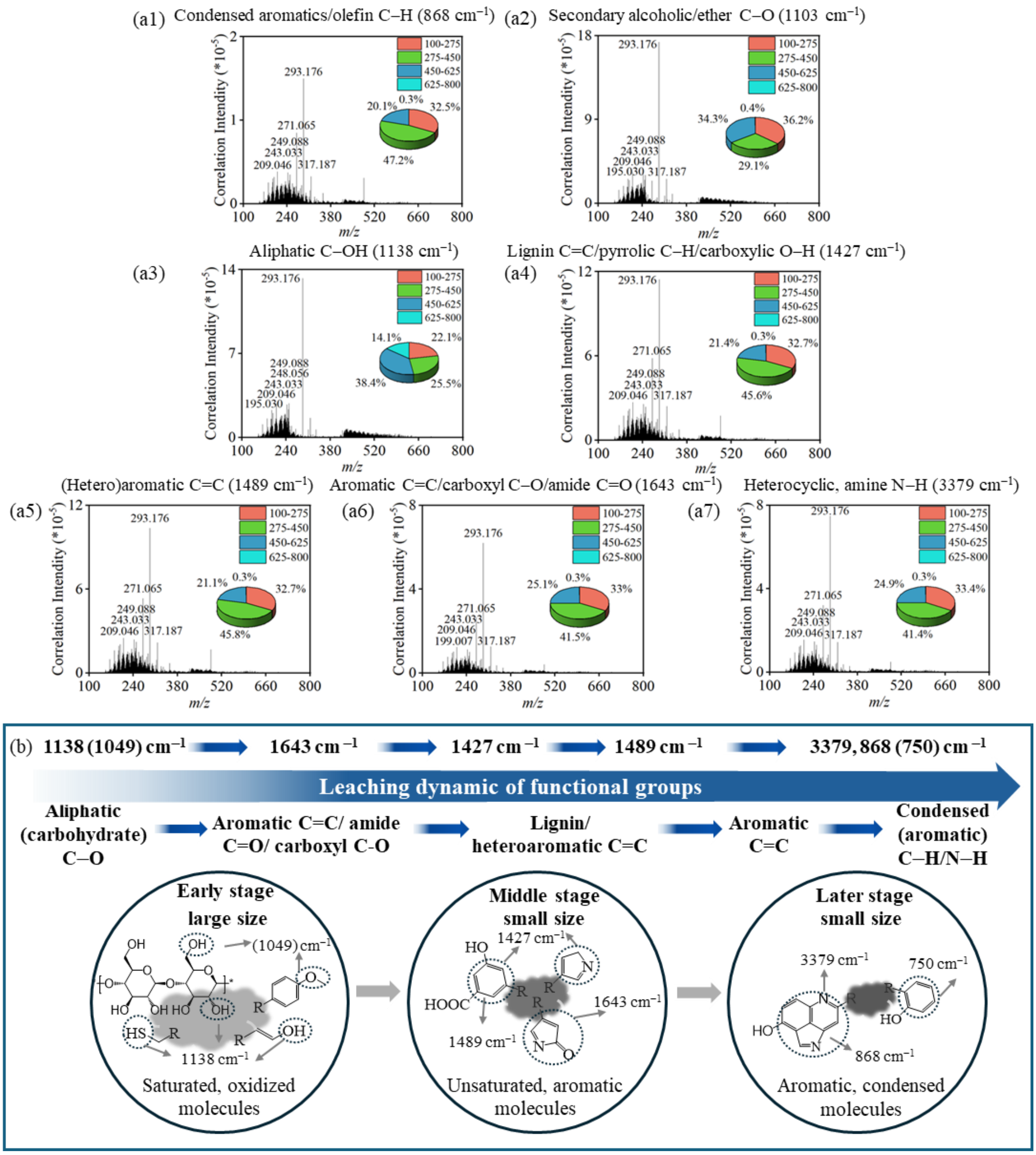

Figure 12. (a) Distribution and *m/z* range percentages of positive heterocorrelations between normalized intensities of molecules and absorbances of major functional groups in DBC350 samples; (b) illustration of the collaborative dynamic leaching mechanisms of DBC350 molecules and functional groups.

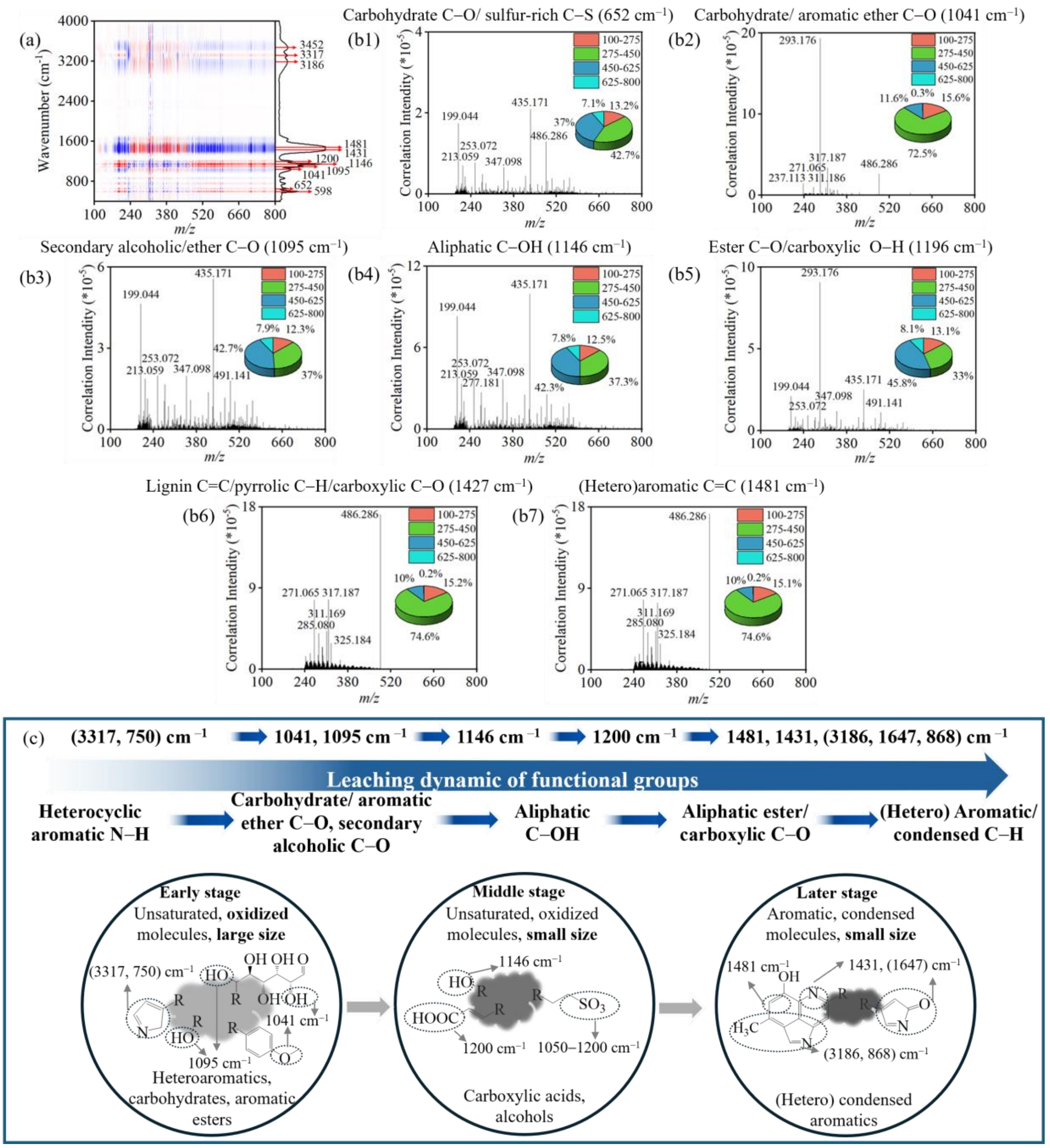

Figure S13. (a) 2D-FT-ICR MS/FTIR-COS heterocorrelation distributions of DBC450; (b) Distribution and *m/z* range percentages of positive heterocorrelations between normalized intensities of molecules and absorbances of major functional groups in DBC450 samples; (c) illustration of the collaborative dynamic leaching mechanisms of DBC450 molecules and functional groups.

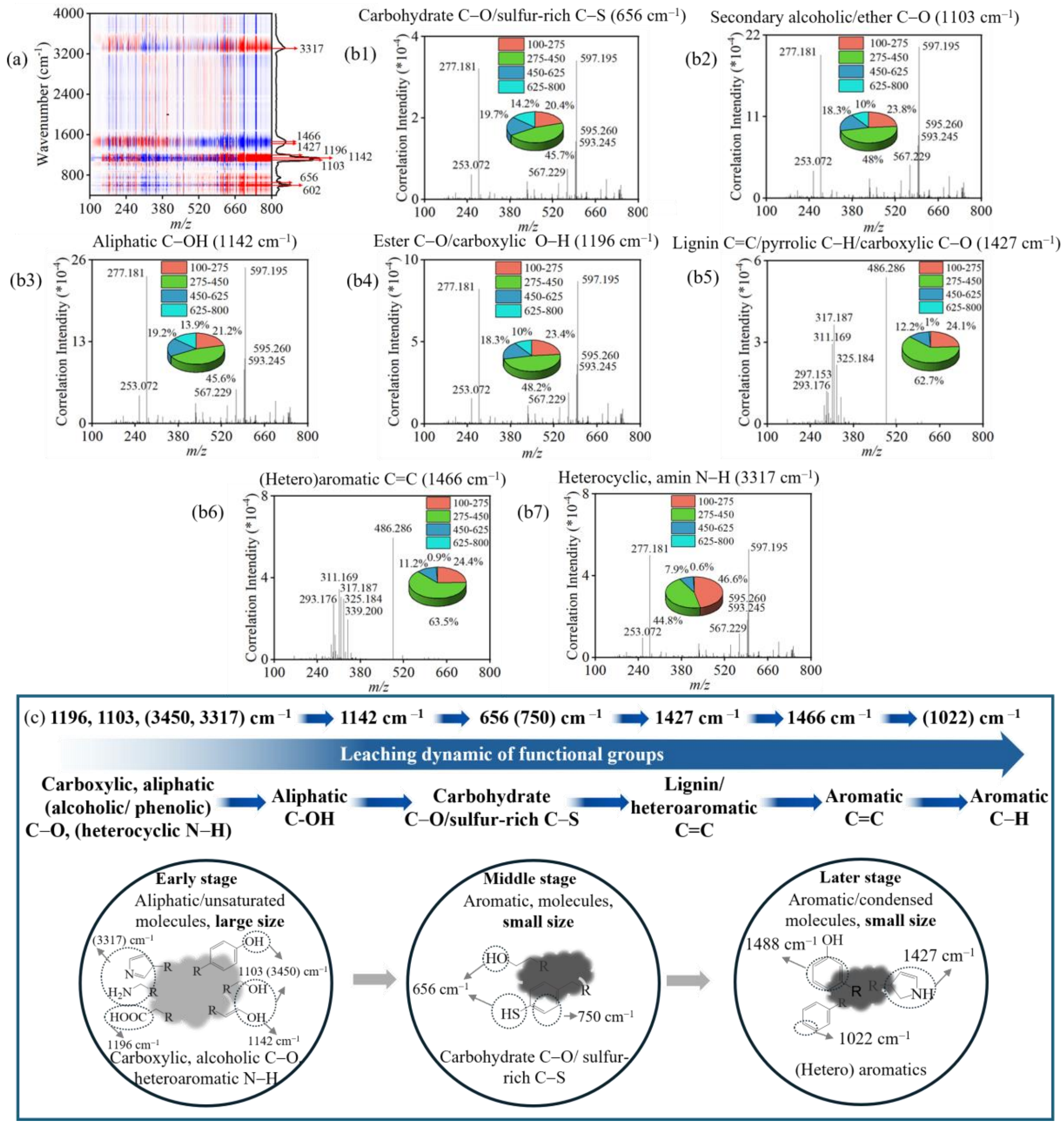

Figure S14. (a) 2D-FT-ICR MS/FTIR-COS heterocorrelation distributions of DBC550; (b) Distribution and *m/z* range percentages of positive heterocorrelations between normalized intensities of molecules and absorbances of major functional groups in DBC550 samples; (c) illustration of the collaborative dynamic leaching mechanisms of DBC550 molecules and functional groups.

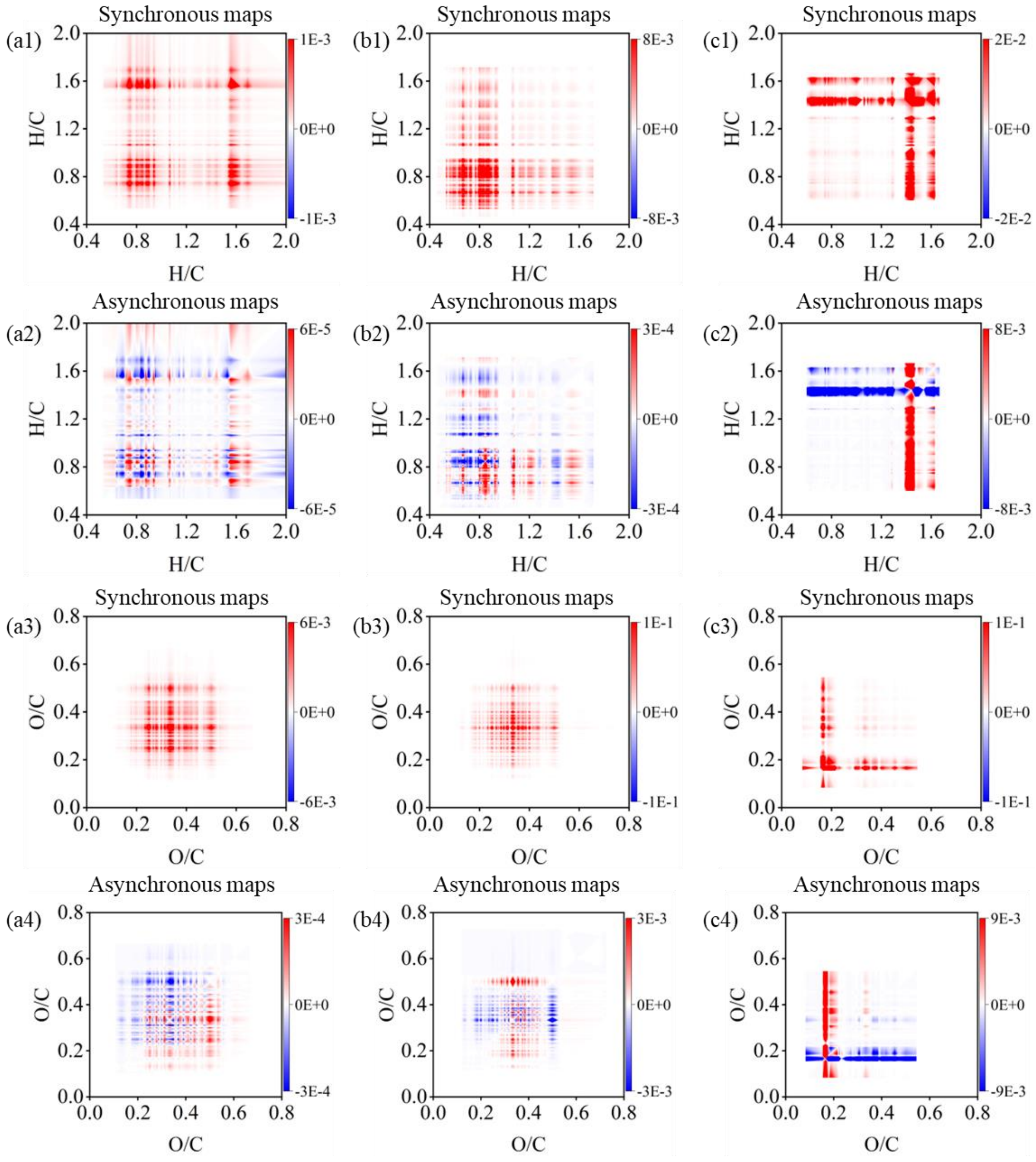

(a) EDC-related DBC350 (b) EDC-related DBC450 (c) EDC-related DBC550

Figure S15. 2D-FTICR-MS-COS maps of EDC-related (a) DBC350, (b) DBC450, and (c) DBC550 molecules based on H/C and O/C ratios.

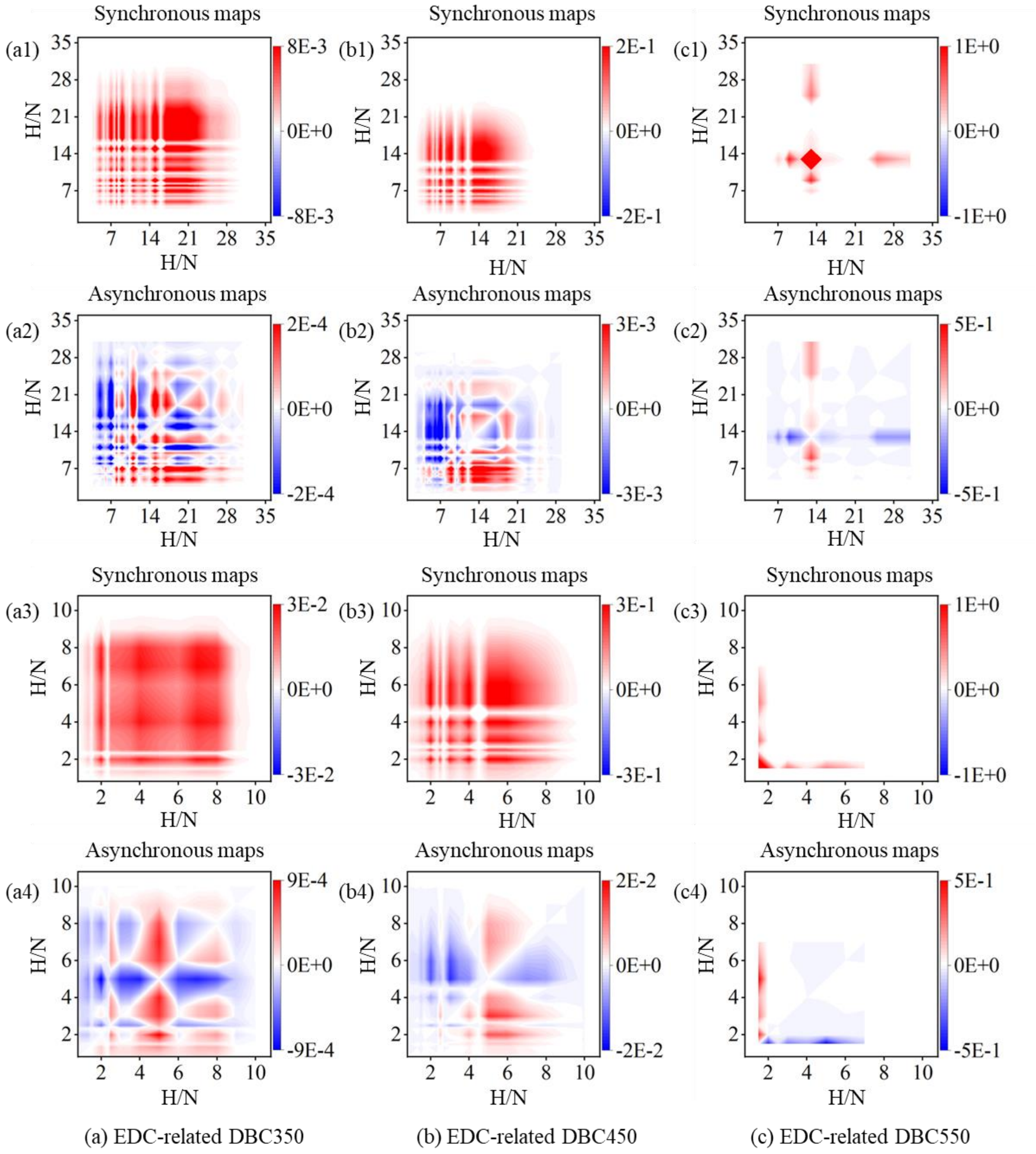

Figure S16. 2D-FTICR-MS-COS maps of EDC-related (a) DBC350, (b) DBC450, and (c) DBC550 molecules based on H/N and O/N ratios.

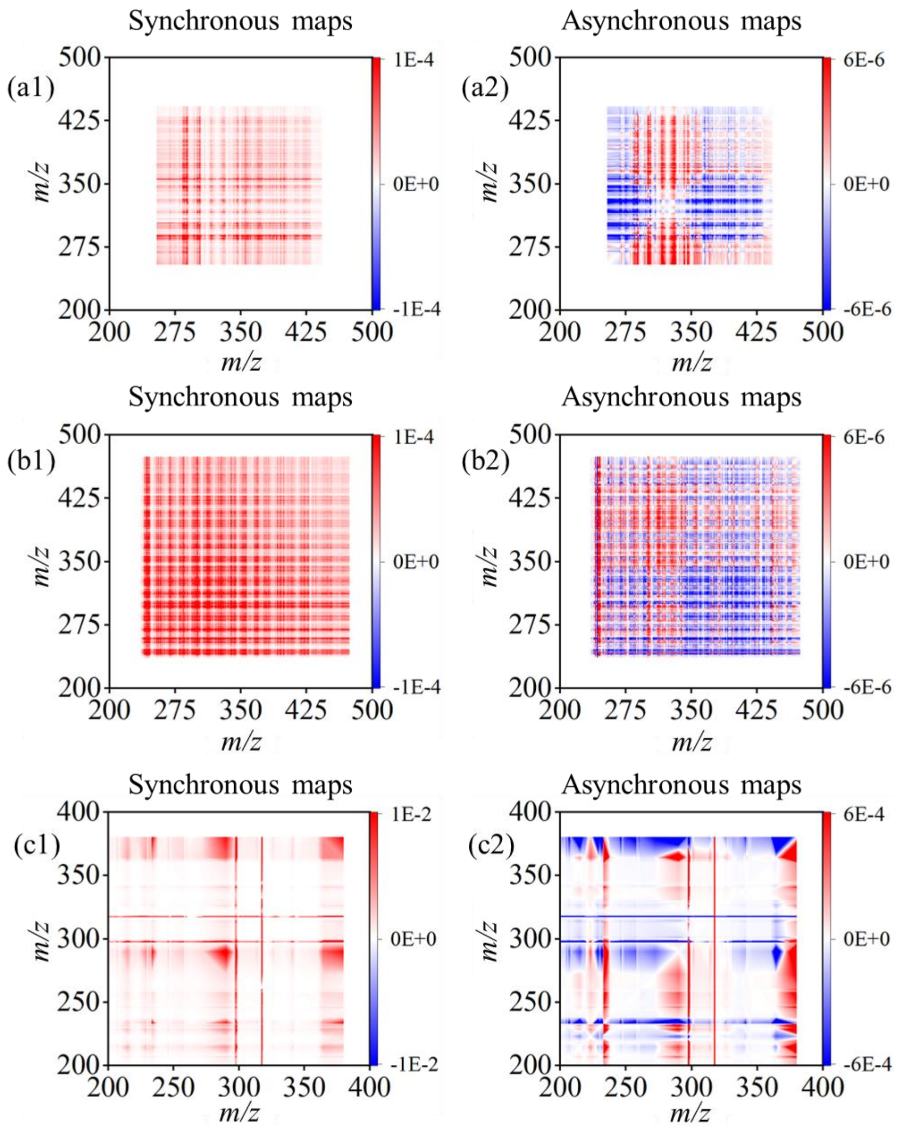

Figure S17. 2D-FTICR-MS-COS maps of EDC-related (a) DBC350, (b) DBC450, and (c) DBC550 molecules based on *m/z*.

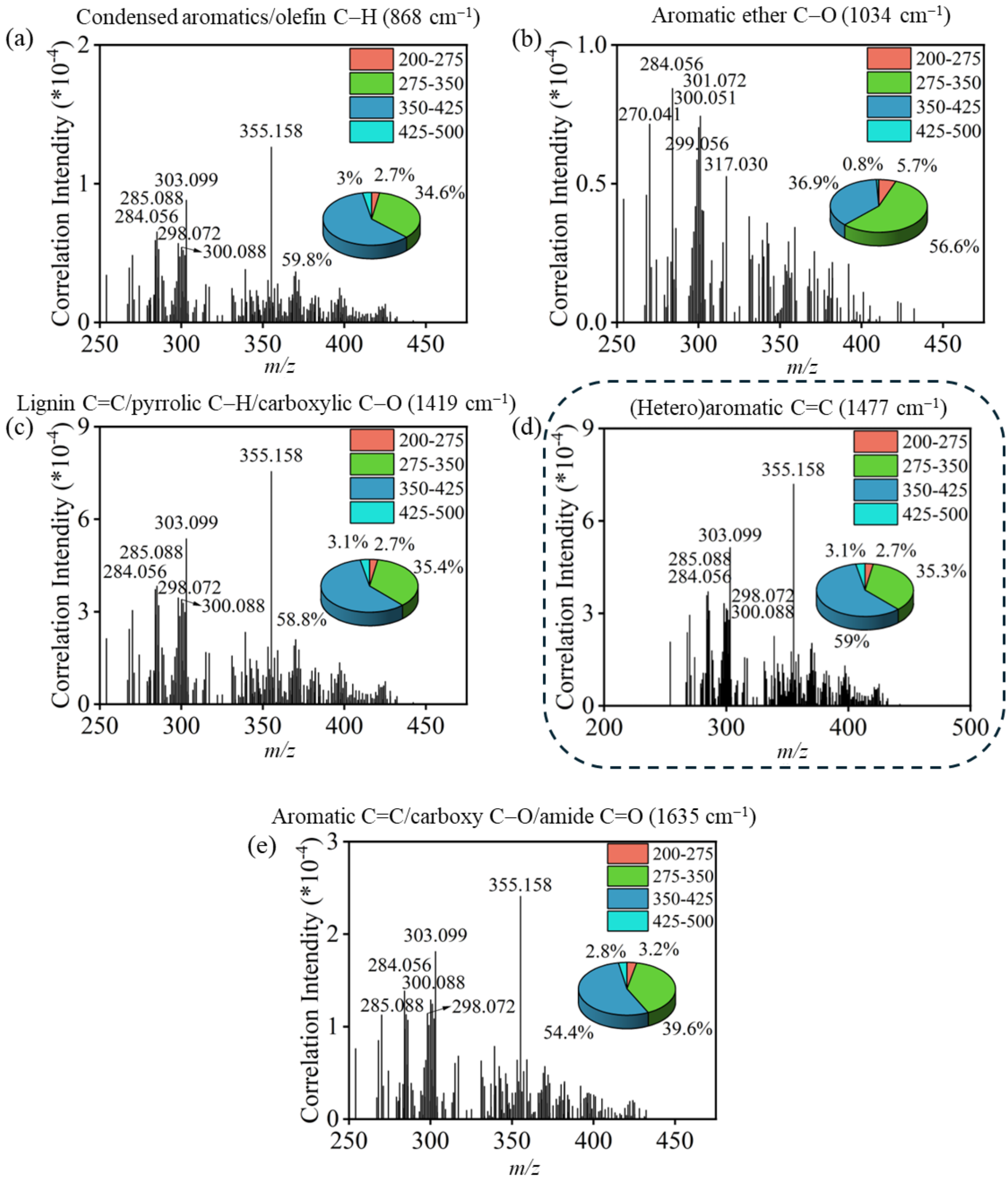

Figure S18. Distribution and *m/z* range percentages of positive heterocorrelations between normalized intensities of EDC-related molecules and FTIR absorbances of major functional groups in DBC350 samples. The representative heterocorrelation distribution at 1477 cm^{-1}, characterized by high intensity and shown in Figure 4c, is highlighted with virtual frames.

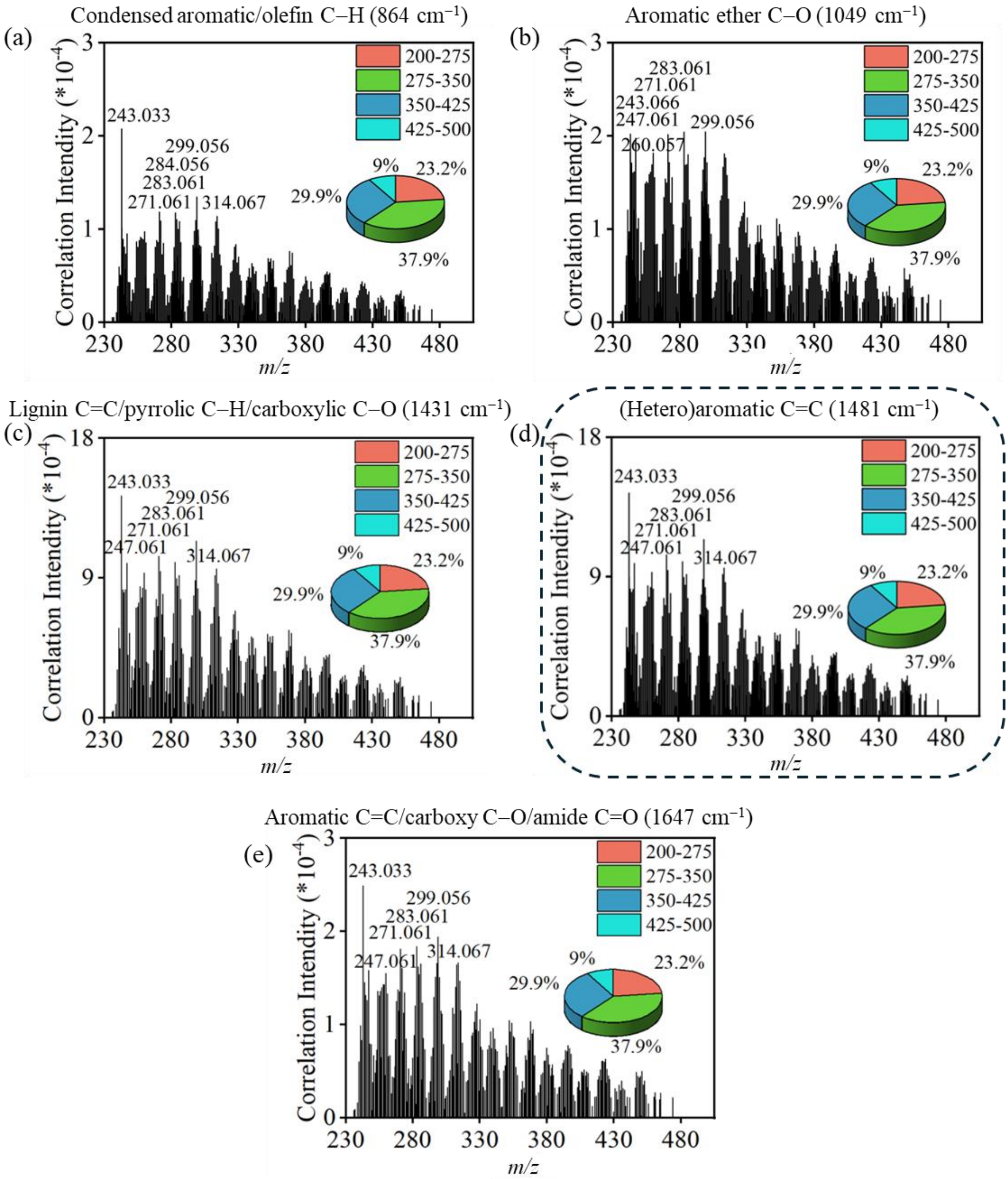

Figure S19. Distribution and *m/z* range percentages of positive heterocorrelations between normalized intensities of EDC-related molecules and FTIR absorbances of major functional groups in DBC450 samples. The representative heterocorrelation distribution at 1481 cm^{-1}, characterized by high intensity and shown in Figure 4c, is highlighted with virtual frames.

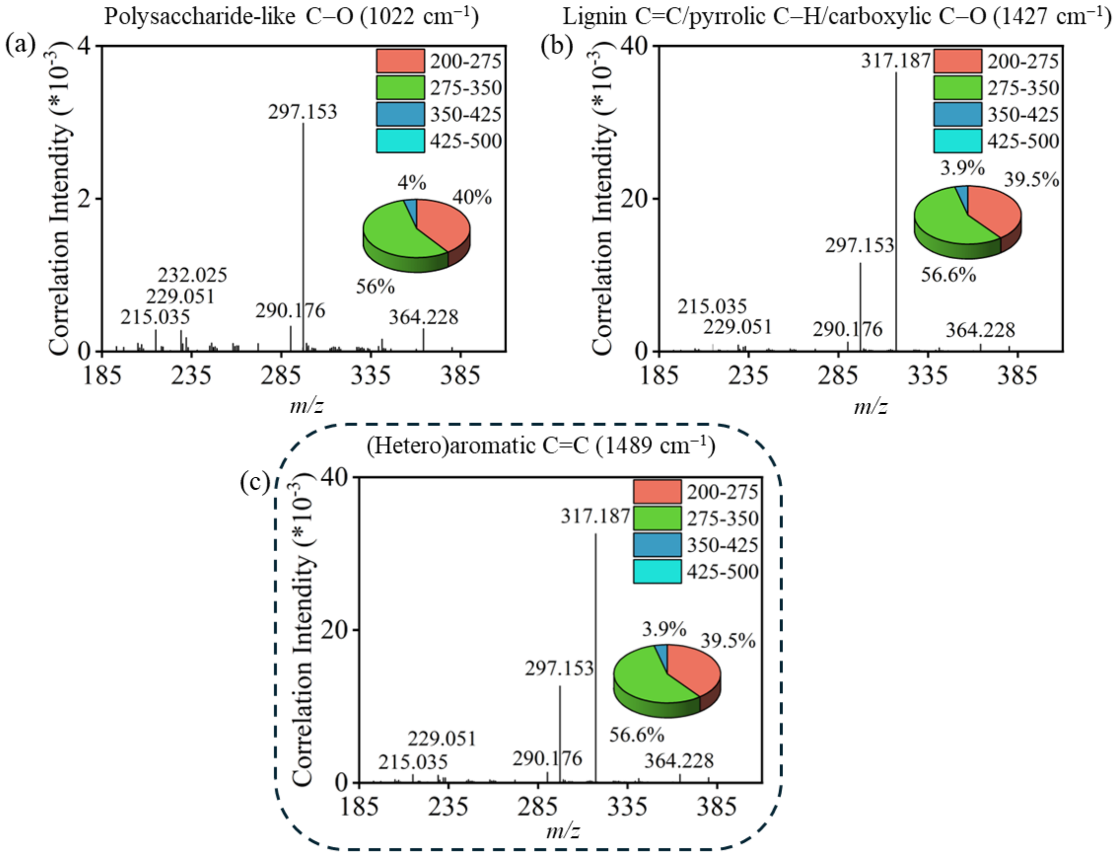

Figure S20. Distribution and *m/z* range percentages of positive heterocorrelations between normalized intensities of EDC-related molecules and FTIR absorbances of major functional groups in DBC350 samples. The representative heterocorrelation distribution at 1489 cm^{-1}, characterized by high intensity and shown in Figure 4c, is highlighted with virtual frames.

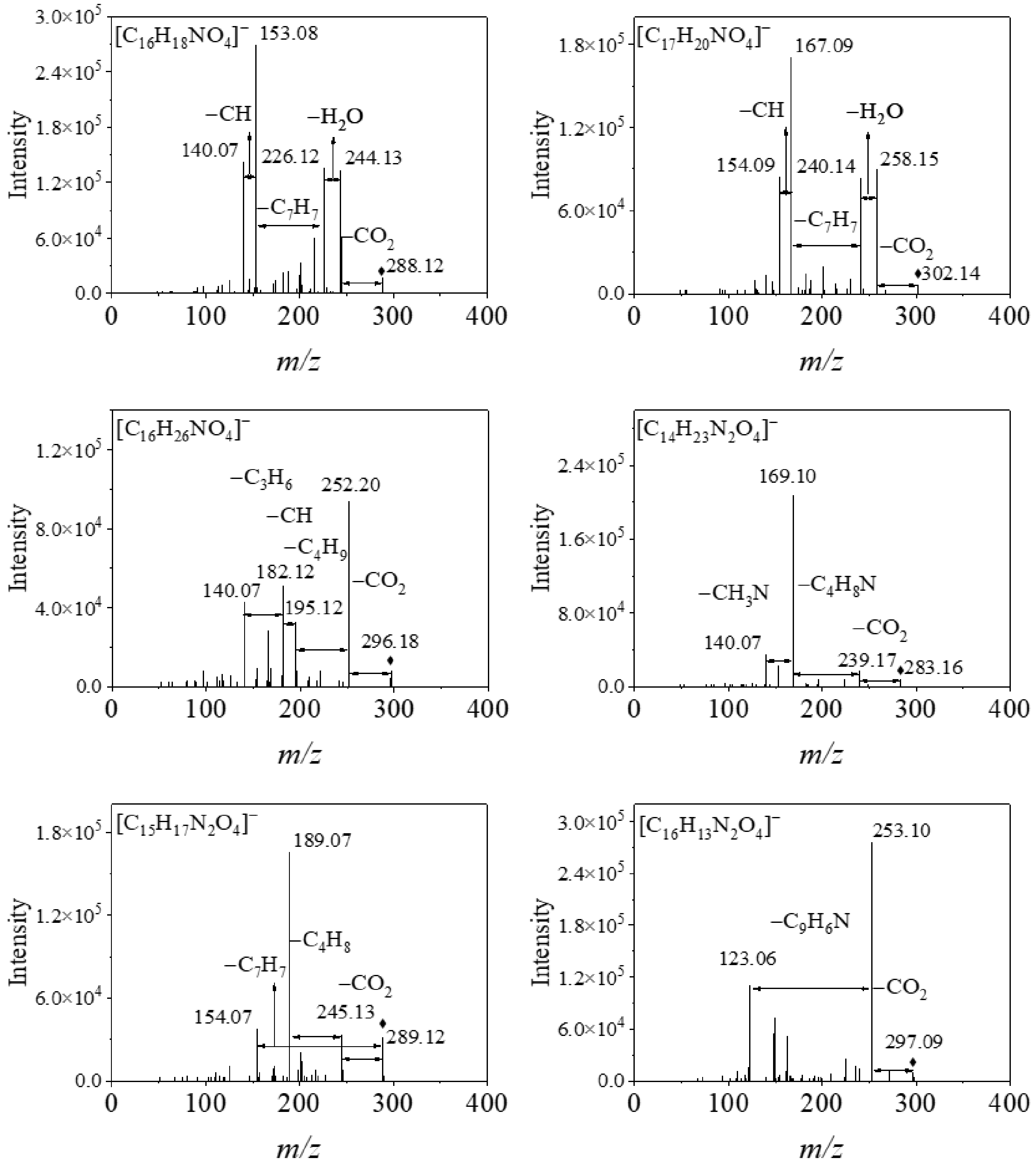

Figure S21. Observed neutral losses during fragmentation of six representative nitrogen-bearing EDC-related molecules with high intensities in DBC350.

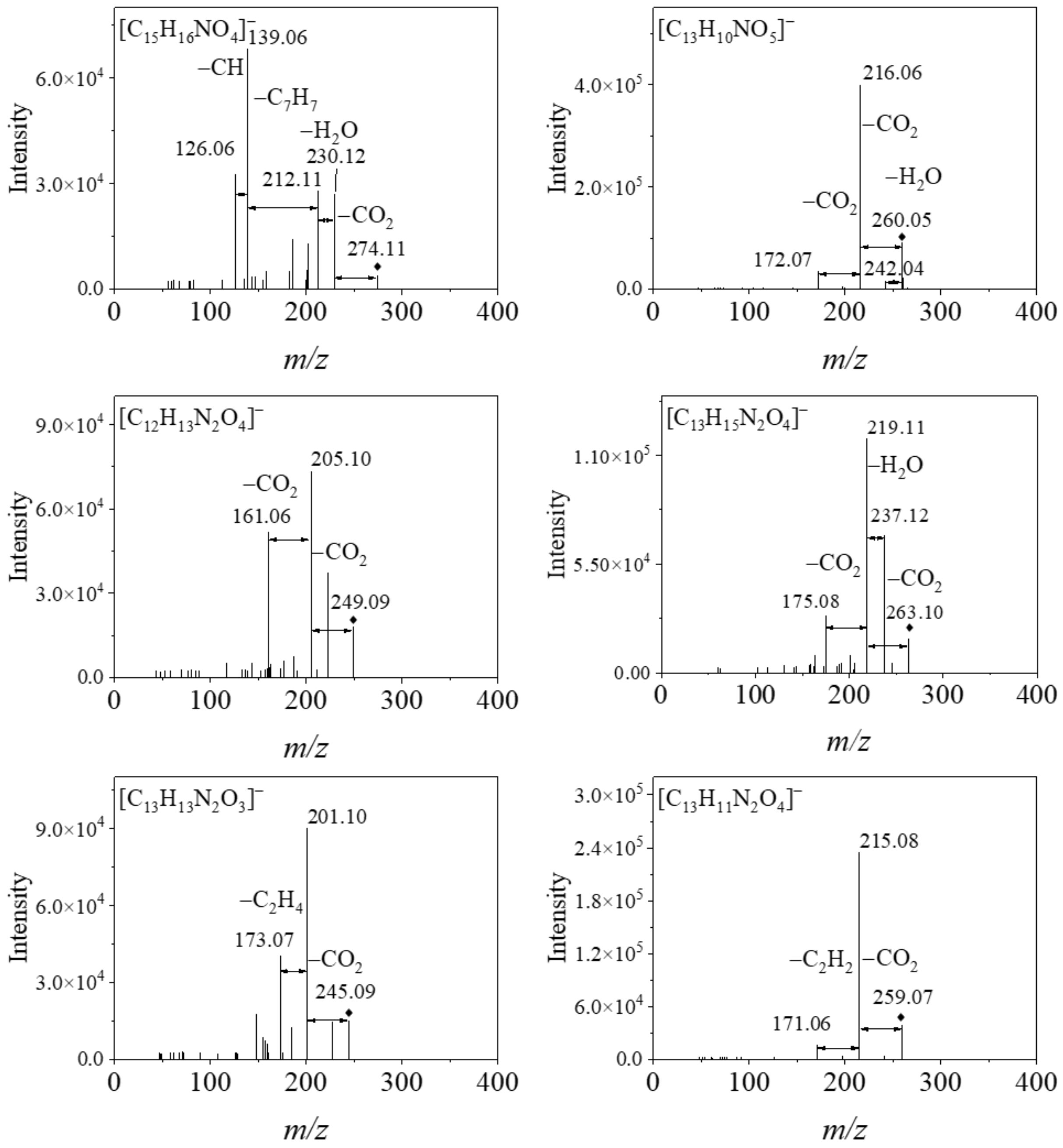

Figure S22. Observed neutral losses during fragmentation of six representative nitrogen-bearing EDC-related molecules with high intensities in DBC450.

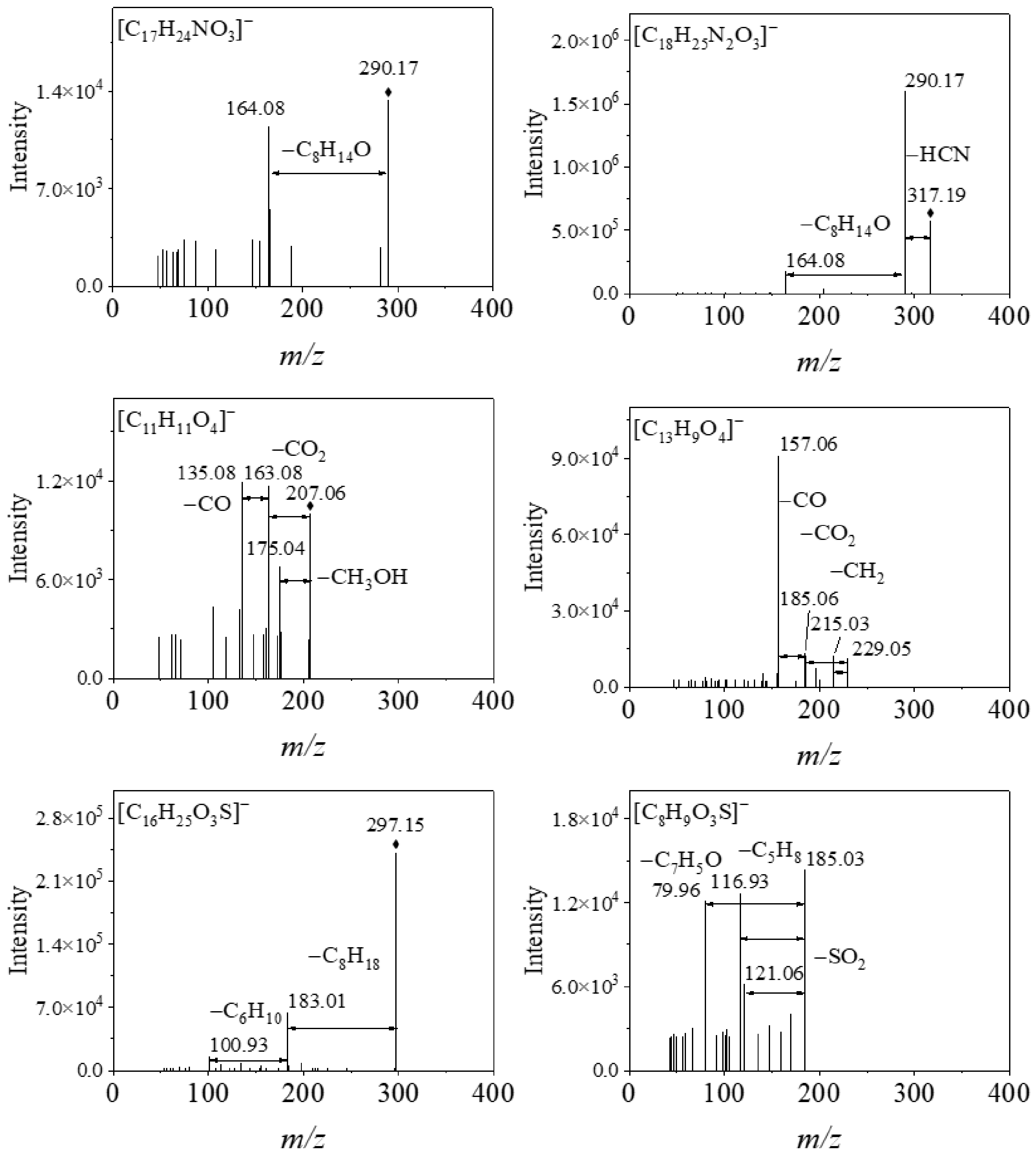

434 **Figure S23.** Observed neutral losses during fragmentation of six representative EDC-related
435 molecules with high intensities in DBC550.

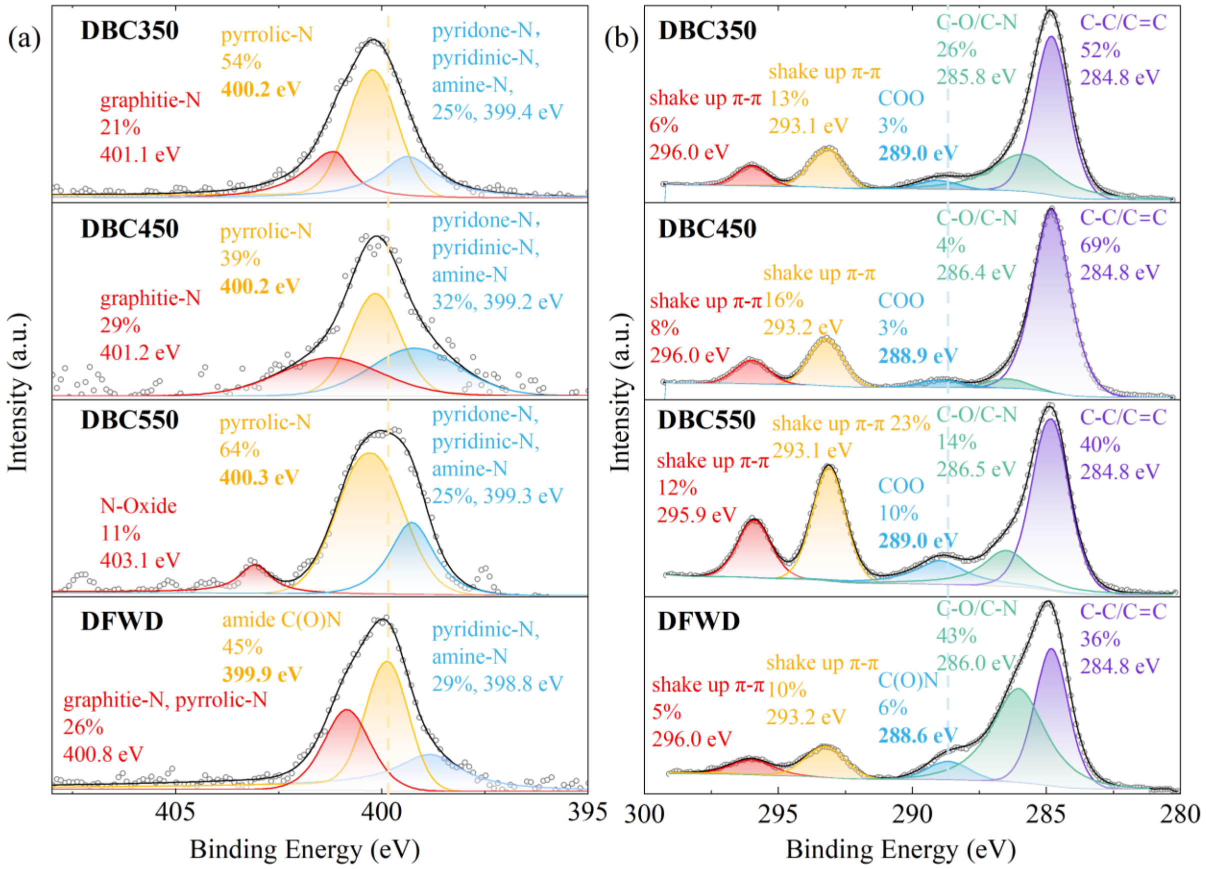

Figure S24. (a) N1s and (b) C1s XPS spectra of the day-2 DBC leachates and DFWD.

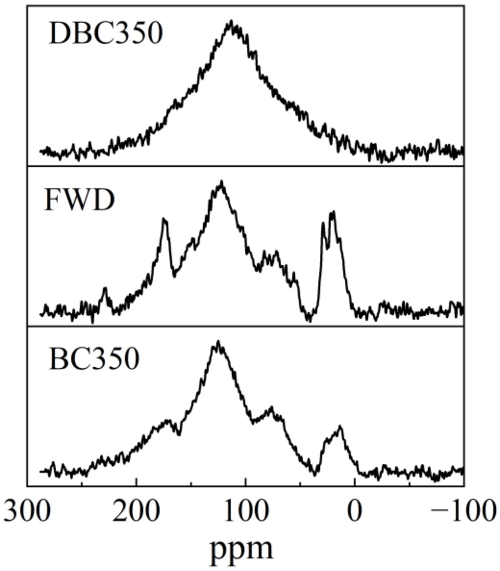

Figure S25. ^{13}C NMR spectra of the day-2 leachate of DBC350, food waste digestate (FWD), and biochar fabricated at 350 °C (BC350).

Table S1. The DOC concentrations and optical parameters of DBC samples

Samples	DOC $mg \cdot L^{-1}$	EDC $mmol_{e-} \cdot g_C^{-1}$	$SUVA_{254}$ $L \cdot mg^{-1} \cdot m^{-1}$	HIX
DBC350-2d	13.69 ± 0.09	1.63 ± 0.18	2.42 ± 0.11	3.31 ± 0.12
DBC350-9d	10.62 ± 0.03	3.12 ± 0.10	4.77 ± 0.31	4.61 ± 0.07
DBC350-16d	3.15 ± 0.37	7.91 ± 0.24	10.17 ± 1.30	4.75 ± 0.05
DBC350-23d	1.56 ± 0.33	15.90 ± 1.24	22.04 ± 1.83	4.66 ± 0.11
DBC350-30d	1.05 ± 0.11	19.87 ± 0.08	26.48 ± 0.85	4.33 ± 0.25
DBC450-2d	17.56 ± 0.24	0.66 ± 0.09	0.81 ± 0.04	3.77 ± 0.01
DBC450-9d	7.73 ± 0.25	1.27 ± 0.04	1.54 ± 0.06	4.06 ± 0.16
DBC450-16d	2.32 ± 0.24	3.24 ± 0.15	3.95 ± 0.30	4.18 ± 0.11
DBC450-23d	3.89 ± 0.27	1.71 ± 0.21	2.21 ± 0.10	3.30 ± 0.06
DBC450-30d	1.03 ± 0.28	3.96 ± 0.61	6.26 ± 0.61	4.17 ± 0.21
DBC550-2d	9.63 ± 0.22	0.26 ± 0.01	0.31 ± 0.06	1.78 ± 0.10
DBC550-9d	4.80 ± 0.64	0.57 ± 0.05	0.43 ± 0.06	1.20 ± 0.05
DBC550-16d	2.22 ± 0.69	1.28 ± 0.05	0.84 ± 0.03	1.28 ± 0.11
DBC550-23d	1.27 ± 0.00	1.43 ± 0.04	1.16 ± 0.07	0.89 ± 0.01
DBC550-30d	1.65 ± 0.14	1.28 ± 0.06	1.36 ± 0.34	1.34 ± 0.06

Table S2. Spearman's *r* between EDC and optical parameters (***: $p < 0.001$, **: $p < 0.01$, and *: $p < 0.05$)

Spearman's r ($n = 15$)	$SUVA_{254}$	Fmax1[a]	Fmax2[a]	Fmax3[a]	HIX
EDC	0.96***	0.84***	−0.86***	0.81***	0.74***
$SUVA_{254}$		0.91***	−0.91***	0.83***	0.83***
Fmax1[a]			−0.99***	0.89***	0.88***
Fmax2[a]				−0.92***	−0.83***
Fmax3[a]					0.61*

[a] Normalized intensity percentage of each EEM-PARAFAC component.

Table S3. Intensity-weighted molecular parameters of DBC samples

Time	O/C_w	H/C_w	N/C_w	H/N_w	O/N_w	DBE_w	$AI_{mod,w}$	MW_w
				DBC350				
2d	0.39	1.18	0.09	12.73	4.06	9.54	0.32	375.58
9d	0.37	1.22	0.08	12.48	3.89	8.63	0.30	356.68
16d	0.38	1.16	0.09	11.66	3.78	8.61	0.34	334.06
23d	0.38	1.18	0.09	11.91	3.79	8.49	0.32	336.98
30d	0.37	1.15	0.09	11.79	3.77	8.88	0.35	338.97
				DBC450				
2d	0.42	1.13	0.06	14.71	5.31	9.46	0.34	370.47
9d	0.35	1.21	0.05	13.83	4.49	8.37	0.33	342.90
16d	0.39	1.08	0.06	12.91	4.71	9.24	0.39	339.38
23d	0.39	1.08	0.06	13.15	4.76	9.32	0.39	344.58
30d	0.37	1.06	0.06	13.15	4.43	9.71	0.41	343.91
				DBC550				
2d	0.38	1.32	0.02	18.08	5.26	9.45	0.26	425.17
9d	0.26	1.55	0.02	16.21	3.27	5.31	0.17	334.20
16d	0.28	1.41	0.04	14.12	2.80	6.48	0.24	326.54
23d	0.30	1.33	0.05	18.01	3.80	7.10	0.39	327.12
30d	0.33	1.43	0.03	20.05	3.90	6.35	0.21	349.91

Table S4. Intensity-weighted molecular parameters of S-bearing and S-free molecules

DBC samples	O/C_w	H/C_w	H/N_w	O/N_w	DBE_w	$AI_{mod,w}$	MW_w
			S-bearing formulas				
DBC350-2d	0.40	1.23	15.99	4.81	9.28	0.25	396.96
DBC350-9d	0.36	1.41	17.34	4.77	5.63	0.17	333.21
DBC350-16d	0.40	1.27	11.98	4.40	5.95	0.23	296.77
DBC350-23d	0.40	1.32	14.70	5.21	6.32	0.20	340.87
DBC350-30d	0.39	1.25	11.72	4.48	6.62	0.24	317.96
DBC450-2d	0.39	1.19	17.59	5.77	9.03	0.29	370.38
DBC450-9d	0.33	1.49	14.94	4.08	5.21	0.19	335.85
DBC450-16d	0.37	1.27	13.49	5.33	6.29	0.25	309.47
DBC450-23d	0.36	1.38	12.64	5.32	5.75	0.21	317.90
DBC450-30d	0.37	1.28	11.99	5.06	6.33	0.26	309.30
DBC550-2d	0.29	1.24	23.82	4.61	12.83	0.31	478.59
DBC550-9d	0.20	1.70	30.43	4.16	3.94	0.09	354.67
DBC550-16d	0.26	1.60	19.67	3.50	4.33	0.11	327.23
DBC550-23d	0.34	1.02	11.00	3.33	11.57	0.45	386.65
DBC550-30d	0.33	1.60	19.53	3.82	4.03	0.11	319.80
			S-free formulas				
DBC350-2d	0.38	1.17	12.46	4.00	9.57	0.33	372.67
DBC350-9d	0.37	1.18	12.22	3.84	9.18	0.33	360.96
DBC350-16d	0.38	1.15	11.65	3.77	8.84	0.35	337.27
DBC350-23d	0.37	1.16	11.76	3.72	8.77	0.34	336.49
DBC350-30d	0.37	1.14	11.79	3.74	9.10	0.36	340.98
DBC450-2d	0.42	1.12	14.52	5.28	9.51	0.35	370.48
DBC450-9d	0.36	1.19	13.81	4.50	8.61	0.34	343.42
DBC450-16d	0.39	1.06	12.90	4.69	9.50	0.40	341.99
DBC450-23d	0.40	1.03	13.18	4.74	9.96	0.42	349.41
DBC450-30d	0.37	1.03	13.19	4.41	10.07	0.43	347.64
DBC550-2d	0.41	1.35	15.80	5.52	8.13	0.24	404.30
DBC550-9d	0.28	1.48	15.51	3.23	5.87	0.20	325.81
DBC550-16d	0.28	1.37	13.97	2.78	6.86	0.26	326.42
DBC550-23d	0.39	1.17	13.48	4.72	8.97	0.37	357.46
DBC550-30d	0.33	1.37	20.06	3.91	7.16	0.24	360.41

Table S5. Intensity-weighted molecular parameters of N-bearing and N-free molecules

DBC samples	O/C_w	H/C_w	H/N_w	O/N_w	DBE_w	$AI_{mod,w}$	MW_w
			N-bearing formulas				
DBC350-2d	0.38	1.18	12.73	4.06	9.88	0.32	383.71
DBC350-9d	0.36	1.19	12.48	3.89	9.33	0.32	367.70
DBC350-16d	0.37	1.16	11.66	3.78	9.06	0.34	346.57
DBC350-23d	0.37	1.18	11.91	3.79	8.74	0.32	339.82
DBC350-30d	0.36	1.15	11.79	3.77	9.20	0.35	346.69
DBC450-2d	0.39	1.10	14.71	5.31	10.36	0.37	386.96
DBC450-9d	0.35	1.12	13.83	4.49	9.77	0.38	359.37
DBC450-16d	0.37	1.05	12.91	4.71	10.00	0.42	349.77
DBC450-23d	0.37	1.04	13.15	4.76	10.25	0.43	354.79
DBC450-30d	0.35	1.04	13.15	4.43	10.29	0.43	351.82
DBC550-2d	0.37	1.16	18.08	5.26	12.43	0.34	447.16
DBC550-9d	0.28	1.41	16.21	3.27	7.30	0.22	354.83
DBC550-16d	0.25	1.32	14.12	2.80	7.94	0.29	336.95
DBC550-23d	0.39	1.29	12.72	3.79	8.09	0.31	367.72
DBC550-30d	0.27	1.32	20.05	3.90	8.20	0.28	365.89
			N-free formulas				
DBC350-2d	0.44	1.20	/	/	7.94	0.31	338.52
DBC350-9d	0.40	1.30	/	/	6.60	0.25	324.63
DBC350-16d	0.42	1.15	/	/	7.06	0.35	289.91
DBC350-23d	0.42	1.18	/	/	7.50	0.32	325.79
DBC350-30d	0.41	1.14	/	/	7.61	0.36	307.40
DBC450-2d	0.46	1.18	/	/	8.09	0.31	345.23
DBC450-9d	0.36	1.32	/	/	6.76	0.27	323.87
DBC450-16d	0.42	1.13	/	/	8.09	0.36	323.73
DBC450-23d	0.41	1.16	/	/	7.97	0.34	329.86
DBC450-30d	0.41	1.09	/	/	8.61	0.38	328.94
DBC550-2d	0.38	1.38	/	/	8.39	0.23	417.38
DBC550-9d	0.25	1.58	/	/	4.83	0.16	329.31
DBC550-16d	0.30	1.46	/	/	5.57	0.21	320.12
DBC550-23d	0.32	1.45	/	/	5.19	0.22	296.21
DBC550-30d	0.36	1.48	/	/	5.50	0.18	342.58

Table S6. Spearman's *r* between EDC and molecular parameters ($n = 15$; ***: $p < 0.001$, **: $p < 0.01$, and *: $p < 0.05$)

Spearman's *r*	O/C_w	H/C_w	N/C_w	H/N_w	O/N_w	DBE_w	$AI_{mod,w}$	MW_w
**EDC**	0.23	-0.57^{*}	0.83^{***}	-0.85^{***}	−0.31	0.21	0.53^{*}	−0.30
O/C_w		-0.73^{**}	0.54^{*}	−0.38	0.67^{**}	0.78^{***}	0.42	0.55^{*}
H/C_w			-0.60^{*}	0.55^{*}	−0.48	-0.77^{***}	-0.82^{***}	−0.21
N/C_w				-0.91^{***}	−0.11	0.43	0.43	0.01
H/N_w					0.27	−0.29	−0.34	0.17
O/N_w						0.65^{**}	0.35	0.75^{**}
DBE_w							0.54^{*}	0.64^{**}
$AI_{mod,w}$								−0.08

Table S7. The primary sequential leaching responses of DBC molecules based on the generalized 2D–COS maps

2D-COS analysis		Cross-region range ($v1/v2$)	Signals (Synchronous/Asynchronous)	Primary sequential temperature responses of signals
DBC350	H/C	1.80–2.10/2.10–2.30	(+/–)	H/C: 2.10–2.30 → 1.80–2.10
	O/C	0.55–1.05/1.05–1.20	(+/–)	O/C: 1.05–1.20 → 0.55–1.05
	H/N	21.5–33.5/35.0–37.5	(+/+)	H/N: 40.0–42.5 → 25.0–40.0 and 21.5–33.5 → 35.0–37.5
		25.0–40.0/40.0–42.5	(+/–)	
	O/N	6.0–7.0/10.0–12.0	(–/+)	O/N: 10.0–12.0 → 6.0–7.0
DBC450	H/C	1.15–1.35/2.00–2.20	(–/+)	H/C: 2.00–2.20 → 1.15–1.35 → 0.45–0.70, 0.80–0.95
		0.45–0.70/1.15–1.35	(–/+)	
		0.80–0.95/1.15–1.35	(–/+)	
	O/C	0.45–0.55/0.55–0.70	(+/–)	O/C: 0.55–0.70 → 0.45–0.55
	H/N	2.5–7.5/23.0–39.0	(–/+)	H/N: 23.0–39.0 → 2.5–7.5 → 39.0–43.0; 21.0–23.0 → 25.0–35.5
		2.5–7.5/39.0–43.0	(+/+)	
		21.0–23.0/25.0–35.5	(+/–)	
	O/N	1.0–2.5/8.5–14.5	(–/+)	O/N: 4.5–5.5 → 8.5–14.5 → 1.5–2.5
		4.5–5.5/8.5–14.5	(+/+)	
DBC550	H/C	/	/	H/C: /
	O/C	0.50–0.60/0.95–1.20	(+/+)	O/C: 0.50–0.60 → 0.95–1.20 and 0.37–0.47 → 0.48–0.50
		0.37–0.47/0.48–0.50	(+/+)	
	H/N	15.0–23.0/31.0–38.5	(+/+)	H/N: 3.0–7.0 → 15.0–23.0 → 31.0–38.5
		3.0–7.0/15.0–23.0	(+/+)	
	O/N	1.5–3.5/10.5–24.0	(–/+)	O/N: 4.0–5.5 → 10.5–24.0 → 1.5–3.5
		4.0–5.5/10.5–24.0	(+/+)	

Note: "+" and "–" represent the positive (red colors) and negative (blue colors) correlations, respectively, in the generalized synchronous and asynchronous 2D-FTICR-MS-COS maps (Figures 3a and S9).

Table S8. Main functional groups identified in FTIR spectra of DBC samples

Peak wavenumber (cm^{-1})	Functional groups	Possible products	References
3700–3000	O–H/ N–H	Phenolic, alcoholic, heterocyclic, amide-, amine- groups	[25,26,46,47]
2932	C–H	Aliphatic structures	[25]
1641	C=C/ C–O/ C=O	Aromatic/ carboxyl/ amide groups	[25]
1482	C=C	Aromatic, heteroatomic structures	[23,27,28]
1426	C=C/ C–H/ O–H	Lignin/ pyrrolic, alkane/ phenolic, carboxylic groups	[48,28,9,24]
1200	O–H/ C–O	Carboxylic/ aliphatic ester groups	[30,31]
1138	C–OH	Aliphatic C–OH	[49]
1100	C–O	Secondary alcoholic/ ether groups	[50]
1200–1050	C–S/ C=S/ S=O	Phenyl sulfide/ thiourea/ sulfoxide groups	[23]
1050	C–O	Carbohydrate and aromatic ether groups	[30–32]
1020	C–H/ C–O	Aromatic/ polysaccharide-like groups	[51,35,32]
866	C–H	Condensed, olefinic, alkene C–H	[24,34]
750	C–H	Aromatic ring with four adjacent hydrogens	[24,33]
650	C–O/ C–S	Carbohydrate, alcoholic/ mercaptan, sulfur-rich groups	[23,30,35]
≤ 600	Si/P/Cl	Inorganics	[24]

Table S9. The primary sequential leaching responses of DBC functional groups based on the generalized 2D–COS maps

Sample	Cross-region range ($v1/v2$, cm^{-1})	Signals (synchronous/ asynchronous)	Primary sequential temperature responses of wavenumbers (cm^{-1})	Primary sequential temperature responses of functional groups
DBC350	3450/3379, 1489, 1427, 868, 750	(+/+)	1138, 1049 → 1643 → 3450, 3150 → 1427 → 1489 → 3379, 868, 750	Aliphatic C−OH, carbohydrate C−O → Amide/ carboxylic C=O → Phenolic/alcoholic O−H, heterocyclic/ amine N−H → Heterocyclic aromatic C=C → Aromatic C=C → Heterocyclic/ amine N−H, condensed C−H, aromatic C−H
	3379/1643, 1489, 1427, 1138	(+/–)		
	3150/1049, 1138	(+/–)		
	3150/3379, 750	(+/+)		
	1643/3450, 3379, 3150, 1489, 1427, 868, 750	(+/+)		
	1643/1138, 1049	(+/–)		
	1489, 1427/ 868, 750	(+/+)		
	1427/3450, 3150	(+/–)		
	1489/ 1427, 1138, 1049	(+/–)		
DBC450	3317/1200, 1146, 1095	(+/+)	3317, 750 → 1041, 1095 → 1146 → 1200 → 868, 1481, 1431, 1647, 3186	Heterocyclic/ amine N−H, aromatic C−H → C−O in carbohydrate, polysaccharides → Aliphatic C−OH → Carboxylic O−H/ aliphatic ester C−O → Condensed C−H, aromatic C=C, carboxylic O−H/ phenolic O−H/ lignin C=C, amide/ carboxylic C=O, heterocyclic/ amine N−H
	3186/1041	(+/–)		
	1481, 1431/1041	(+/–)		
	1146, 1095/750	(+/–)		
	1095, 1041/868	(+/+)		
	3186/1200, 1146, 1095	(–/+)		
	1647/1200, 1146, 1095	(–/+)		
	1481, 1431/1095, 1200, 1146	(–/+)		
	1200/1041	(–/+)		
	1200, 1146, 1095/ 868	(–/–)		
	1146/1200	(+/+)		
	1041/750	(–/+)		
	1095/750	(+/–)		
DBC550	3450/3317	(+/+)	3450 → 3317 → 1196, 1103 → 1142 → 750, 656 → 1427 → 1466 → 1022	Alcoholic/ phenolic O−H → Heterocyclic/ amine N−H → Carboxylic O−H/ aliphatic ester C−O, secondary alcoholic C−O → Aliphatic C−OH → Aromatic ring with four adjacent hydrogen, carbohydrate C−O/ sulfur-rich C−S → Lignin, heteroaromatic C−H/ phenolic O−H → Aromatic C=C → Aromatic C−H, polysaccharide-like C−O groups
	3317/1142, 750	(+/+)		
	3317/1466, 1427	(–/–)		
	1466/1196, 1142, 1103	(–/+)		
	1466/1427	(+/–)		
	1427/1196, 1103	(–/+)		
	1196/1142	(+/+)		
	1142/1103	(+/–)		
	750, 656/1103, 1142	(+/–)		
	750, 656/1427	(–/–)		
	1022/1466, 1427	(+/–)		

Note: "+" and "–" represent the positive (red colors) and negative (blue colors) correlations, respectively, in the generalized synchronous and asynchronous 2D-FTIR-COS maps (Figures 3c and S11b–S11c).

Table S10. The primary sequential leaching responses of EDC-related DBC molecules based on the generalized 2D–COS maps

2D-COS analysis		Cross-region range ($v1/v2$)	Signals (Synchronous/ Asynchronous)	Primary sequential temperature responses of signals
DBC350	H/C	0.71–1.74/0.64–0.70	(+/+)	H/C: 0.71–1.74 → 0.64–0.70
	O/C	0.48–0.56/0.23–0.36	(+/+)	O/C: 0.48–0.56 → 0.23–0.36 → 0.11–0.23
		0.23–0.56/0.11–0.23	(+/+)	
	H/N	4.0–8.0/8.0–30.0	(+/–)	H/N: 10.5–11.5→ 14.5–15.5→ 16.5–26.5→ 4.0–8.0
		14.5–15.5/16.5–26.5	(+/+)	
		10.5–11.5/11.5–26.5	(+/+)	
	O/N	0.8–1.4/2.5–8.5	(+/–)	O/N: 4.5–5.5 → 5.5–8.5 → 3.0–3.5 → 0.8–1.4; 3.8–5.8, 6.5–8.5→ 1.6–2.2
		1.6–2.2/3.8–5.8, 6.5–8.5	(+/–)	
		3.0–3.5/3.5–8.5	(+/–)	
		4.5–5.5/5.5–8.5	(+/+)	
DBC450	H/C	0.83–1.23/0.53–0.73	(+/+)	H/C: 0.83–1.23/1.47–1.63 → 0.53–0.73
		1.47–1.63/0.53–1.06	(+/+)	
	O/C	0.27–0.44/0.17–0.27	(+/+)	O/C: 0.27–0.44 → 0.17–0.27 → 0.47–0.52
		0.47–0.52/0.17–0.47	(+/–)	
	H/N	4.5–7.5/8.0–21.0	(+/–)	H/N: 18.0–20.0 → 12.0–15.0 → 15.0–18.0 → 4.5–7.5
		12.0–15.0/15.0–18.0	(+/+)	
		12.0–15.0/18.0–20.0	(+/–)	
	O/N	1.5–2.5, 2.8–3.5/3.5–4.5, 5.0–9.0	(+/–)	O/N: 4.6–6.6 → 3.5–4.5 → 7.6–8.6 → 1.5–2.5, 2.8–3.5
		4.6–6.6/3.5–4.5, 6.0–9.0	(+/+)	
		3.5–4.5/ 7.6–8.6	(+/+)	
DBC550	H/C	0.60–1.28/1.38–1.63	(+/–)	H/C: 1.38–1.63 → 0.60–1.28
	O/C	0.25–0.54/0.15–0.22	(+/–)	O/C: 0.15–0.22 → 0.25–0.54; 0.15–0.35 → 0.08–0.15
		0.15–0.35/0.08–0.15	(+/+)	
	H/N	6.0–8.0, 9.0–11.0/11.0–15.0	(+/–)	H/N: 11.0–15.0 → 6.0–8.0, 9.0–11.0; 11.5–14.5 → 25.0–31.0, 14.5–18.5
		11.5–14.5/14.5–18.5	(+/+)	
		11.5–14.5/25.0–31.0	(+/+)	
	O/N	1.5–1.9/2.0–7.0	(+/+)	O/N: 1.5–1.9 → 2.0–7.0

Note: "+" and "–" represent the positive (red colors) and negative (blue colors) correlations, respectively, in the generalized synchronous and asynchronous 2D-FTICR-MS-COS maps (Figures S15–S16).

Table S11. Intensity-weighted molecular parameters of EDC-related molecules

	O/C_w	H/C_w	N/C_w	H/N_w	O/N_w	DBE_w	$AI_{mod,w}$	MW_w
				DBC350				
EDC-all	0.34	0.99	0.07	13.26	4.39	10.86	0.47	353.32
EDC-N0	0.40	0.98	0.00	/	/	10.32	0.46	356.46
EDC-N1	0.34	0.99	0.05	18.37	6.29	10.85	0.47	354.59
EDC-N2	0.32	0.99	0.12	8.66	2.78	11.01	0.48	345.40
EDC-N3	0.27	1.04	0.16	6.50	1.67	11.38	0.46	364.27
				DBC450				
EDC-all	0.33	0.92	0.08	10.95	3.93	10.80	0.52	324.65
EDC-N0	0.35	0.88	0.00	/	/	10.37	0.54	308.21
EDC-N1	0.33	0.90	0.06	15.52	5.70	11.00	0.53	327.64
EDC-N2	0.32	0.96	0.13	7.82	2.68	10.69	0.50	326.53
EDC-N3	0.33	0.94	0.18	5.33	1.85	11.54	0.51	350.45
				DBC550				
EDC-all	0.26	1.24	0.07	13.37	2.59	7.45	0.35	293.49
EDC-N0	0.32	1.14	0.00	/	/	6.86	0.40	262.47
EDC-N1	0.34	1.00	0.07	15.62	4.85	8.80	0.49	287.64
EDC-N2	0.18	1.40	0.11	12.41	1.60	7.23	0.27	314.75
EDC-N3	0.33	1.50	0.17	9.00	2.00	7.00	0.08	277.73

Rreferences

(1) Chen, S.; Du, Y.; Das, P.; Lamore, A. F.; Dimova, N. T.; Elliott, M.; Broadbent, E. N.; Roebuck., J. A.; Jaffé, R.; Lu, Y. Agricultural Land Use Changes Stream Dissolved Organic Matter via Altering Soil Inputs to Streams. *Sci. Total Environ.* **2021**, *796*, 148968.

(2) Cao, T.; Li, M.; Xu, C.; Song, J.; Fan, X.; Li, J.; Jia, W.; Peng, P. Technical Note: Chemical Composition and Source Identification of Fluorescent Components in Atmospheric Water-Soluble Brown Carbon by Excitation–Emission Matrix Spectroscopy with Parallel Factor Analysis – Potential Limitations and Applications. *Atmospheric Chem. Phys.* **2023**, *23* (4), 2613–2625.

(3) Zhang, X.; Kang, J.; Chu, W.; Zhao, S.; Shen, J.; Chen, Z. Spectral and Mass Spectrometric Characteristics of Different Molecular Weight Fractions of Dissolved Organic Matter. *Sep. Purif. Technol.* **2020**, *253*, 117390.

(4) Shutova, Y.; Baker, A.; Bridgeman, J.; Henderson, R. K. Spectroscopic Characterisation of Dissolved Organic Matter Changes in Drinking Water Treatment: From PARAFAC Analysis to Online Monitoring Wavelengths. *Water Res.* **2014**, *54*, 159–169.

(5) Weishaar, J. L.; Aiken, G. R.; Bergamaschi, B. A.; Fram, M. S.; Fujii, R.; Mopper, K. Evaluation of Specific Ultraviolet Absorbance as an Indicator of the Chemical Composition and Reactivity of Dissolved Organic Carbon. *Environ. Sci. Technol.* **2003**, *37* (20), 4702–4708.

(6) Chen, W.; Westerhoff, P.; Leenheer, J. A.; Booksh, K. Fluorescence Excitation−Emission Matrix Regional Integration to Quantify Spectra for Dissolved Organic Matter. *Environ. Sci. Technol.* **2003**, *37* (24), 5701–5710.

(7) Zsolnay, A.; Baigar, E.; Jimenez, M.; Steinweg, B.; Saccomandi, F. Differentiating with Fluorescence Spectroscopy the Sources of Dissolved Organic Matter in Soils Subjected to Drying. *Chemosphere* **1999**, *38* (1), 45–50.

(8) Murphy, K. R.; Stedmon, C. A.; Graeber, D.; Bro, R. Fluorescence Spectroscopy and Multi-Way Techniques. PARAFAC. *Anal. Methods* **2013**, *5* (23), 6557–6566.

(9) Zhang, X.; Xu, Z.; Sun, Y.; Mohanty, S. K.; Lei, H.; Khan, E.; Tsang, D. C. W. Implications of Pyrolytic Gas Dynamic Evolution on Dissolved Black Carbon Formed During Production of Biochar from Nitrogen-Rich Feedstock. *Environ. Sci. Technol.* **2025**, *59* (5), 2699–2710.

(10) Wu, S.; Wang, D.; Liu, C.; Fang, G.; Sun, T.-R.; Cui, P.; Yan, H.; Wang, Y.; Zhou, D. Pyridinic- and Pyrrolic Nitrogen in Pyrogenic Carbon Improves Electron Shuttling during Microbial Fe(III) Reduction. *ACS Earth Space Chem.* **2021**, *5* (4), 900–909.

(11) Heike Knicker. "Black Nitrogen" – an Important Fraction in Determining the Recalcitrance of Charcoal. *Org. Geochem.* **2010**, *41* (9), 947–950.

(12) Knicker, H.; Hilscher, A.; González-Vila, F. J.; Almendros, G. A New Conceptual Model for the Structural Properties of Char Produced during Vegetation Fires. *Org. Geochem.* **2008**, *39* (8), 935–939.

(13) Wagner, S.; Dittmar, T.; Jaffé, R. Molecular Characterization of Dissolved Black Nitrogen via Electrospray Ionization Fourier Transform Ion Cyclotron Resonance Mass Spectrometry. *Org. Geochem.* **2015**, *79*, 21–30.

(14) Bahureksa, W.; Young, R. B.; McKenna, A. M.; Chen, H.; Thorn, K. A.; Rosario-Ortiz, F. L.; Borch, T. Nitrogen Enrichment during Soil Organic Matter Burning and Molecular Evidence of Maillard Reactions. *Environ. Sci. Technol.* **2022**, *56* (7), 4597–4609.

(15) Lian, F.; Zhang, Y.; Gu, S.; Han, Y.; Cao, X.; Wang, Z.; Xing, B. Photochemical Transformation and Catalytic Activity of Dissolved Black Nitrogen Released from Environmental Black Carbon. *Environ. Sci. Technol.* **2021**, *55* (9), 6476–6484.

(16) Qu, X.; Fu, H.; Mao, J.; Ran, Y.; Zhang, D.; Zhu, D. Chemical and Structural Properties of Dissolved Black Carbon Released from Biochars. *Carbon* **2016**, *96*, 759–767.

(17) Osterholz, H.; Niggemann, J.; Giebel, H.-A.; Simon, M.; Dittmar, T. Inefficient

Microbial Production of Refractory Dissolved Organic Matter in the Ocean. *Nat. Commun.* **2015**, *6* (1), 7422.

(18) Koch, B. P.; Dittmar, T. From Mass to Structure: An Aromaticity Index for High-Resolution Mass Data of Natural Organic Matter. *Rapid Commun. Mass Spectrom.* **2016**, *30* (1), 250–250.

(19) Koch, B. P.; Dittmar, T. From Mass to Structure: An Aromaticity Index for High-Resolution Mass Data of Natural Organic Matter. *Rapid Commun. Mass Spectrom.* **2006**, *20* (5), 926–932.

(20) Zhang, X.; Han, J.; Zhang, X.; Shen, J.; Chen, Z.; Chu, W.; Kang, J.; Zhao, S.; Zhou, Y. Application of Fourier Transform Ion Cyclotron Resonance Mass Spectrometry to Characterize Natural Organic Matter. *Chemosphere* **2020**, *260*, 127458.

(21) Lv, J.; Zhang, S.; Wang, S.; Luo, L.; Cao, D.; Christie, P. Molecular-Scale Investigation with ESI-FT-ICR-MS on Fractionation of Dissolved Organic Matter Induced by Adsorption on Iron Oxyhydroxides. *Environ. Sci. Technol.* **2016**, *50* (5), 2328–2336.

(22) Wu, W.; Wang, K.; Liu, J.; So, P.-K.; Leung, T.-F.; Wong, M.; Zhao, D. A High-Throughput Integrated Nontargeted Metabolomics and Lipidomics Workflow Using Microelution Enhanced Matrix Removal-Lipid for Comparative Analysis of Human Maternal and Umbilical Cord Blood Metabolomes. *Anal. Chem.* **2025**, *97* (5), 2629–2638.

(23) Bellamy, L. *The Infra-Red Spectra of Complex Molecules*; Springer Science & Business Media, 2013.

(24) Nair, R. R.; Mondal, M. M.; Weichgrebe, D. Biochar from Co-Pyrolysis of Urban Organic Wastes—Investigation of Carbon Sink Potential Using ATR-FTIR and TGA. *Biomass Convers. Biorefinery* **2022**, *12* (10), 4729–4743.

(25) Wu, S.; You, F.; Boughton, B.; Liu, Y.; Nguyen, T. A. H.; Wykes, J.; Southam, G.; Robertson, L. M.; Chan, T.-S.; Lu, Y.-R.; Lutz, A.; Yu, D.; Yi, Q.; Saha, N.; Huang, L. Chemodiversity of Dissolved Organic Matter and Its Molecular Changes Driven by Rhizosphere Activities in Fe Ore Tailings Undergoing Eco-Engineered Pedogenesis. *Environ. Sci. Technol.* **2021**, *55* (19), 13045–13060.

(26) Keiluweit, M.; Nico, P. S.; Johnson, M. G.; Kleber, M. Dynamic Molecular Structure of Plant Biomass-Derived Black Carbon (Biochar). *Environ. Sci. Technol.* **2010**, *44* (4), 1247–1253.

(27) Rawat, P.; Bharati, P.; Gautam, A.; Kumar, M.; Singh, R.; Prakash; Ram, A.; Gautam, S.; Darwari, A.; Mishra, A.; Singh, R. N. Design and Synthesis of Pyrazole, Pyrazolone and 1,3,4-Oxadiazole Derivatives Having Pyrrole Motif as a Source of New Antimicrobial and Anticancer Agents. *J. Mol. Struct.* **2023**, *1272*, 134087.

(28) Zhou, C.; Zheng, H.; Chen, Y.; Mao, G.; Deng, G.-J. Modular Synthesis of Tetrasubstituted Pyrroles through a Four-Component Cyclization Strategy Using Ammonium Salt as the Nitrogen Source. *J. Org. Chem.* **2023**, *88* (3), 1533–1544.

(29) Parikh, S. J.; Goyne, K. W.; Margenot, A. J.; Mukome, F. N. D.; Calderón, F. J. Soil Chemical Insights Provided through Vibrational Spectroscopy. In *Advances in Agronomy*; Elsevier, 2014; Vol. 126, pp 1–148.

(30) Abdulla, H. A. N.; Minor, E. C.; Dias, R. F.; Hatcher, P. G. Changes in the Compound Classes of Dissolved Organic Matter along an Estuarine Transect: A Study Using FTIR and ^{13}C NMR. *Geochim. Cosmochim. Acta* **2010**, *74* (13), 3815–3838.

(31) Duarte, R. M. B. O.; Santos, E. B. H.; Pio, C. A.; Duarte, A. C. Comparison of Structural Features of Water-Soluble Organic Matter from Atmospheric Aerosols with Those of Aquatic Humic Substances. *Atmos. Environ.* **2007**, *41* (37), 8100–8113.

(32) Smith, B. The C-O Bond III: Ethers By a Knockout. **2017**, *32*, 22–26.

(33) Song, F.; Li, T.; Wu, F.; Leung, K. M. Y.; Hur, J.; Zhou, L.; Bai, Y.; Zhao, X.; He, W.; Ruan, M. Temperature-Dependent Molecular Evolution of Biochar-Derived Dissolved

Black Carbon and Its Interaction Mechanism with Polyvinyl Chloride Microplastics. *Environ. Sci. Technol.* **2023**, *57* (18), 7285–7297.

(34) Chen, W.; Habibul, N.; Liu, X.-Y.; Sheng, G.-P.; Yu, H.-Q. FTIR and Synchronous Fluorescence Heterospectral Two-Dimensional Correlation Analyses on the Binding Characteristics of Copper onto Dissolved Organic Matter. *Environ. Sci. Technol.* **2015**, *49* (4), 2052–2058.

(35) Smith, B. The C-O Bond, Part I: Introduction and the Infrared Spectroscopy of Alcohols. **2017**, *32*, 14–21.

(36) Li, T.; Ruan, M.; Cao, Y.; Feng, W.; Song, F.; Bai, Y.; Zhao, X.; Wu, F. Molecular-Level Insights into the Temperature-Dependent Formation Dynamics and Mechanism of Water-Soluble Dissolved Organic Carbon Derived from Biomass Pyrolysis Smoke. *Water Res.* **2024**, *252*, 121176.

(37) Sleighter, R. L.; Cory, R. M.; Kaplan, L. A.; Abdulla, H. A. N.; Hatcher, P. G. A Coupled Geochemical and Biogeochemical Approach to Characterize the Bioreactivity of Dissolved Organic Matter from a Headwater Stream. J. Geophys. Res. Biogeosciences 2014, 119 (8), 1520–1537.

(38) dos Santos, J. V.; Goranov, A. I.; Fregolente, L. G.; Bisinoti, M. C.; Sun, Z.; Schmidt-Rohr, K.; Hatcher, P. G. Deciphering the Chemistry of Condensed Aromatic "Black" Carbon and Nitrogen in Amazonian Anthrosols. *Environ. Sci. Technol.* **2025**, *59* (32), 17047–17058.

(39) Jansen, R. J. J.; van Bekkum, H. XPS of Nitrogen-Containing Functional Groups on Activated Carbon. *Carbon* **1995**, *33* (8), 1021–1027.

(40) Singh, B.; Fang, Y.; Cowie, B. C. C.; Thomsen, L. NEXAFS and XPS Characterisation of Carbon Functional Groups of Fresh and Aged Biochars. *Org. Geochem.* **2014**, *77*, 1–10.

(41) Cecchet, F.; Pilling, M.; Hevesi, L.; Schergna, S.; Wong, J. K. Y.; Clarkson, G. J.; Leigh, D. A.; Rudolf, P. Grafting of Benzylic Amide Macrocycles onto Acid-Terminated Self-Assembled Monolayers Studied by XPS, RAIRS, and Contact Angle Measurements. *J. Phys. Chem. B* **2003**, *107* (39), 10863–10872.

(42) Rück-Braun, K.; Petersen, M. Å.; Michalik, F.; Hebert, A.; Przyrembel, D.; Weber, C.; Ahmed, S. A.; Kowarik, S.; Weinelt, M. Formation of Carboxy- and Amide-Terminated Alkyl Monolayers on Silicon(111) Investigated by ATR-FTIR, XPS, and X-Ray Scattering: Construction of Photoswitchable Surfaces. *Langmuir* **2013**, *29* (37), 11758–11769.

(43) Zhang, X.; Liu, Z.; Khan, H. I. U. haq; Barati, B.; Parakhonskiy, B.; Skirtach, A. G.; Rousseau, D. P. L.; Van Hulle, S. Synthesis, Characterization, and Comparison of N-Modified Biochar with Different Nitrogen Sources for Bisphenol A Adsorption. *Biomass Convers. Biorefinery* **2025**, *15* (3), 3517–3532.

(44) Almendros, G.; Knicker, H.; González-Vila, F. J. Rearrangement of Carbon and Nitrogen Forms in Peat after Progressive Thermal Oxidation as Determined by Solid-State ^{13}C- and ^{15}N-NMR Spectroscopy. *Org. Geochem.* **2003**, *34* (11), 1559–1568.

(45) Mateev, E.; Angelov, B.; Kondeva-Burdina, M.; Valkova, I.; Georgieva, M.; Zlatkov, A. DESIGN, Synthesis, Biological Evaluation and Molecular Docking of Pyrrole-Based Compounds as Antioxidant and Mao-B Inhibitory Agents. *Farmacia* **2022**, *70,* 344–354.

(46) Bakshi, S.; Banik, C.; Laird, D. A.; Smith, R.; Brown, R. C. Enhancing Biochar as Scaffolding for Slow Release of Nitrogen Fertilizer. *ACS Sustain. Chem. Eng.* **2021**, *9* (24), 8222–8231.

(47) Noguchi, T.; Sugiura, M. Analysis of Flash-Induced FTIR Difference Spectra of the S-State Cycle in the Photosynthetic Water-Oxidizing Complex by Uniform ^{15}N and ^{13}C Isotope Labeling. *Biochemistry* **2003**, *42* (20), 6035–6042.

(48) Guo, X.-J.; He, X.-S.; Li, C.-W.; Li, N.-X. The Binding Properties of Copper and

Lead onto Compost-Derived DOM Using Fourier-Transform Infrared, UV–Vis and Fluorescence Spectra Combined with Two-Dimensional Correlation Analysis. *J. Hazard. Mater.* **2019**, *365*, 457–466.

(49) Yu, G.-H.; Wu, M.-J.; Wei, G.-R.; Luo, Y.-H.; Ran, W.; Wang, B.-R.; Zhang, J.; Shen, Q.-R. Binding of Organic Ligands with Al(III) in Dissolved Organic Matter from Soil: Implications for Soil Organic Carbon Storage. *Environ. Sci. Technol.* **2012**, *46* (11), 6102–6109.

(50) D'Orazio, V.; Senesi, N. Spectroscopic Properties of Humic Acids Isolated from the Rhizosphere and Bulk Soil Compartments and Fractionated by Size-Exclusion Chromatography. *Soil Biol. Biochem.* **2009**, *41* (9), 1775–1781.

(51) Jung, M. R.; Horgen, F. D.; Orski, S. V.; Rodriguez C., V.; Beers, K. L.; Balazs, G. H.; Jones, T. T.; Work, T. M.; Brignac, K. C.; Royer, S.-J.; Hyrenbach, K. D.; Jensen, B. A.; Lynch, J. M. Validation of ATR FT-IR to Identify Polymers of Plastic Marine Debris, Including Those Ingested by Marine Organisms. *Mar. Pollut. Bull.* **2018**, *127*, 704–716.
